# A measure of reliability convergence to select and optimize cognitive tasks for individual differences research

**DOI:** 10.1038/s44271-024-00114-4

**Published:** 2024-07-04

**Authors:** Jan Kadlec, Catherine R. Walsh, Uri Sadé, Ariel Amir, Jesse Rissman, Michal Ramot

**Affiliations:** 1 Department of Brain Sciences, Weizmann Institute of Science, Rehovot, Israel; 2 Department of Psychology, University of California, Los Angeles, CA USA; 3 Faculty of Physics, Weizmann Institute of Science, Rehovot, Israel; 4 Department of Psychiatry and Biobehavioral Sciences, University of California, Los Angeles, CA USA; 5 Present Address: Section on Functional Imaging Methods, National Institute of Mental Health, Bethesda, MD USA

**Keywords:** Cognitive neuroscience, Human behaviour

## Abstract

Surging interest in individual differences has faced setbacks in light of recent replication crises in psychology, for example in brain-wide association studies exploring brain-behavior correlations. A crucial component of replicability for individual differences studies, which is often assumed but not directly tested, is the reliability of the measures we use. Here, we evaluate the reliability of different cognitive tasks on a dataset with over 250 participants, who each completed a multi-day task battery. We show how reliability improves as a function of number of trials, and describe the convergence of the reliability curves for the different tasks, allowing us to score tasks according to their suitability for studies of individual differences. We further show the effect on reliability of measuring over multiple time points, with tasks assessing different cognitive domains being differentially affected. Data collected over more than one session may be required to achieve trait-like stability.

## Abstract (web summary)

Reliability of cognitive task measures improves as a function of number of trials. Because of differences in reliability convergence, tasks differ in suitability as estimates of individual differences. To achieve traitlike stability in measures, data must be combined across sessions.

## Introduction

One of the longstanding mysteries in cognitive science is what drives the profound variation in behavior that can be observed on almost any task, even within the typically developing population^1–4^. The search for the neural underpinnings of these individual differences, i.e., the neural variance between individuals that explains the behavioral variance, has been one of the key motivating forces behind cognitive neuroscience research in the last couple of decades^5–9^. Such brain-behavior association studies have demonstrated links between individual neural activation levels during tasks^10–12^ and behavioral performance, as well as between resting state brain networks and behavior^13–15^. Behavioral data on cognitive tasks are also increasingly being used for applications like predicting risk taking^16^, adherence to public health measures^17^, academic achievement^18,19^, externalizing problems in children^20^, and diagnosis of diseases such as Alzheimer’s Disease^21,22^.

This fascination with individual differences, which are found in animals as well as humans^23–25^, is driven not only by the deepest philosophical questions regarding individuality and our conception of the self but also by more practical considerations. Understanding individual differences will increase the utility of personalized biomarkers and treatments, which form the basis of precision medicine, and could revolutionize diagnosis and care for many psychiatric disorders^26–29^. Yet this endeavor of linking brain and behavior has recently come under fire^30^, most notably through its occasionally problematic implementation in brain-wide association studies which carry out data-driven, whole brain searches for such correlations. Criticism of the approach has centered on the small effect sizes, which can require thousands of participants in order to reveal a reproducible association. Similar problems are faced in most other fields utilizing behavior to predict outcome or infer diagnoses, for example in the study of schizophrenia^31^, depression^32,33^, or psychopathology^34^.

For neuroimaging data, recent publications offer a variety of paths forward. One suggestion was that we must either settle for such very small effect sizes across very large populations (as in genome-wide association studies), or abandon the study of individual differences altogether, focusing instead on within-participant studies^35^. Others have highlighted the advantages of moving to more multivariate neural measures, especially when considering complex behaviors^36–38^, or the need to increase the reliability of the neural data^39,40^. Yet the effects of the reliability of the behavioral measurement, the oft-ignored second variable in this brain-behavior relationship, have garnered far less attention, although a few recent papers have begun to address this gap^41–48^.

Focusing on functional magnetic resonance imaging (fMRI) and taking a step back from this discussion, we must first consider what exactly it is we are measuring with brain-behavior correlations. In many cases, these correlations are calculated between a neural state and a behavior that are not measured simultaneously. This is always the case when the behavior is compared to resting state measures, but it is often also true when the neural state is derived from task-based measures^14,39,49,50^. Given the constraints of fMRI scanning, behavioral measures are usually collected outside the scanner, sometimes on different days than the scan itself. The underlying assumption for any such brain-behavior correlation to be meaningful must therefore be that we are measuring stable, trait-like behaviors. This is also true for individual differences studies which attempt to link cognitive tasks with diagnoses or other stable traits such as personality profiles.

Many researchers administering cognitive tasks do not explicitly verify whether the behaviors they are measuring indeed converge to a stable mean at the individual level, i.e., whether internal consistency is high. If performance on a task was stable at the individual level, then we would expect that when averaging over a sufficient number of trials, we would reach a stable estimate of participants’ ability, resulting in high split-halves or test-retest reliability. However, when obtained, test-retest reliability measures for most tasks are modest at best (~0.6 reliability^51–61^). Split-halves reliability (comparing a random half of the data to the other half) could be affected by momentary, non-specific fluctuations in a non-measured variable such as attention, arousal, and motivation. Test-retest reliability (comparing scores across different testing times) would be even more sensitive to such state changes, for instance in attention, arousal, motivation, etc. While this might be considered measurement error, such variables could interact directly with the ability being tested, especially in complex cognitive tasks. For example, attention could be considered an inherent process involved in memory tasks, directly influencing one’s ability to encode the stimuli, rather than an independent variable. This would mean that any fluctuations in measured scores could be a true change in abilities (e.g., ability to remember the stimuli) rather than measurement error, making these two difficult to separate (for instance see discussion in Lord and Novick^62^). For test-retest reliability measures, there could also be additional longitudinal change in the true measured ability, especially as time between measurements increases.

Examining how estimates of split-halves or test-retest reliabilities converge as more trials are added will allow us to test whether the low reliability scores reported for cognitive tasks are a function of insufficient data being used to estimate individual proficiencies, or whether they are the result of true change in individual abilities. This question of convergence is crucial not only for its impact on correlational analyses such as the relationship between brain and behavior, but also in itself. Practically, the rate at which performance at the individual level on a particular task converges, affects the amount of data we must collect in order to reliably estimate trait-like abilities at the individual level. Beyond that though, the rate of convergence adds information regarding the nature of the ability being measured, and the utility of the task for separating individuals^60^. Although more data enhances reliability, it must be balanced against available time and resources. The convergence rate directly influences what this trade-off function looks like, and making it explicit should aid researchers in optimizing their data collection strategies.

Lastly, in order to examine trait-like stability of a cognitive ability, measures must be repeated across days, as has been stressed in previous studies^63–68^. While some studies have tried to estimate transient change over days and model true trait-like performance^69,70^, it remains unclear whether changes across measurement times differentially affect distinct tasks or cognitive domains^70^ and how this affects the ceiling of test-retest reliability for different tasks over different time frames. It is also unknown whether such changes across days fluctuate around a stable mean, i.e., whether test-retest reliability will improve if averaged over several different sessions / days. If this is indeed the case, it is an open question as to how many sessions would be needed for a reliable estimate of the true ceiling of test-retest reliability.

To address these questions, we collected data from more than 250 participants on a battery of 12 commonly used cognitive tasks, along with two new tasks we created, yielding a total of 21 different behavioral measures (some tasks produced multiple measures) spanning multiple cognitive domains. This allowed us to directly compare different tasks in the same individuals. To increase the number of trials for each task while minimizing the effect of learning across trials, we used multiple alternate forms of most tasks. For each behavioral measure, we tested the reliability using a permutation-based split-halves analysis. In order to directly test the effect of time on repeated measures (with alternate forms), we included data on a task for which multiple forms were collected at different time scales. To test how averaging across days affects convergence, we examine an additional dataset on one of our new tasks, in which data were collected for each participant over six different testing days.

We aimed to evaluate whether all tasks tested would eventually converge to stable performance at the individual level with enough trials, and to characterize the rate of convergence between tasks. Our approach involved reformulating the Spearman-Brown prophecy to define a convergence coefficient $C$, allowing us to directly compare convergence across different tasks in the same individuals. We further defined this convergence coefficient analytically using only the mean and variance of the distribution for measures that are binary at the single trial level. We validated the analytical model using both extensive simulations and real behavioral data. Additionally, we tested the model on increasingly smaller subsamples of both the simulated and the real-world data to anticipate potential error arising from small sample sizes to predict reliability even with minimal data.

In order to improve the quality of behavioral data and behavioral tasks used by the community, we have developed an easy-to-use online tool based on the analytical model, which can calculate reliability given data from any existing dataset including small pilot experiments (https://jankawis.github.io/reliability-web-app/). Our hope is that this will facilitate better planning and design of behavioral tasks by providing researchers an estimate of the number of trials necessary to reach any desired reliability level. This tool can be used to inform study design before committing to larger and more expensive studies.

## Methods

### Real-world behavioral tasks

Since both the tool and the derived formula are as general as possible, and in order to make this tool widely applicable, we tested it on known tasks across many domains to cover a wide spread of cognitive measures. Included tasks are detailed in Table 1 (including average time to completion and number of participants with one and more forms), and measures are summarized below. For some of the tasks, we examine multiple measures which reflect different aspects of performance (e.g., inhibitory control vs. sustained attention). In order to acquire a sufficient number of trials per participant to estimate the true reliability of each task and correlations between tasks, we either administered the task multiple times (SCAP, CCMT), administered extended task versions that included additional trials with novel stimuli (GFMT, Emotion Matching and Emotion Labeling) or administered additional versions of the task with novel stimuli (MST, PGNG, RISE, PIM, FMP, CFMT, VET). Additionally, we provide detailed descriptions of the tasks and measures with examples and references at https://jankawis.github.io/battery_of_tasks_WIS_UCLA/intro.html. Throughout the text and figures, if not otherwise specified, we use the name or acronym for a given task to denote participants’ overall accuracy on that task (e.g., PGNG); additional task performance measures, when used, are denoted with the name/acronym plus additional specification (e.g., PGNG PCTT; PGNG PCIT), see Supplementary Table 2. for list of all abbreviations.

**Table 1:** Table summarizing all tasks in our battery (first column) and the primary cognitive constructs (second column) that each is thought to estimate

Task	Construct	Time [min]	Number of participants with	
			1 form	2 or more forms
Car Matching Test^79^	object perception	3	250	173
Cambridge Car Memory Test (CCMT)^75^	object memory	7	243	154
Vanderbilt Expertise Test (VET)^129^	object memory	7	119	106
Mnemonic Similarity Task (MST)^77^	object memory	11	231	84
Relational and Item-Specific Encoding (RISE)^80^	object/associative memory	7	242	109
**Personal Identity Memory Task (PIM)**	face memory/ associative memory	35	249	152
**Face Memory/Perception Task (FMP)**	face memory/perception	41	223	119
Glasgow Face Matching Test (GFMT)^53^	face perception	4, 8	238	157
Cambridge Face Memory Test (CFMT)^2,81,82^	face memory/perception	7	234	58
Emotion Matching^83^	social cognition	3	249	161
Emotion Labeling^83^	social cognition	6	242	166
Spatial Working Memory Capacity (SCAP)^71^	working memory	12	247	162
N-Back^74^	working memory	10	164	93
Parametric Go/No Go (PGNG)^51,73^	working memory, attention	14	237	152

Bolded items reflect the two custom tasks developed for this dataset. The third column details the mean time to complete a single form of this task (in the case of GFMT, it represents the time needed to complete the short and long versions of GFMT, respectively). The last two columns then detail how many participants completed at least one form of the task (left) or two or more forms (right; indicates how many participants completed all available forms).

Working memory:

- Spatial Working Memory Capacity (SCAP)^71^: Cowan’s K^72^ (maximum across loads), and overall accuracy.
- Parametric Go/No Go Task (PGNG)^51,73^: percent correct to target (PCTT), percent correct to inhibitory trials (PCIT), calculated across levels, and overall accuracy.
- Fractal N-Back^74^: d’ for 2-back, and overall accuracy calculated across levels.

Object memory:

- Cambridge Car Memory Test (CCMT)^75^: overall accuracy.
- Vanderbilt Expertise Test (VET; collapsed across birds, leaves, and planes subtests)^76^: overall accuracy.
- Mnemonic Similarity Task (MST)^77^: recognition accuracy score (REC), lure discrimination index (LDI) and overall accuracy. Note that here we used the traditional version of the MST; a new version (oMST^78^), which we did not test, is now available.

Object (car) perception:

- Car Matching Test^79^: overall accuracy.

Object/associative memory:

- Relational and Item-Specific Encoding (RISE)^80^: overall accuracy on relational blocks. Note that we didn’t include the second form in the analyses due to its poor reliability (*R* = 0.26).

Face memory:

- Personal Identity Memory Task (PIM): overall accuracy on multiple choice attribute recollection test (PIM MC); face recognition accuracy scaled by confidence (PIM recog).
- Face Memory/Perception Task (FMP): overall accuracy

Face perception and memory:

- Glasgow Face Matching Test (GFMT)^53^: overall accuracy.
- Cambridge Face Memory Test (CFMT): overall accuracy; data included from original^2^, Australian^81^ and Female^82^ test forms.

Social cognition:

- Emotion Labeling and Emotion Matching Tasks^83^: overall accuracy.

### Data collection

This study was not pre-registered. All data were collected online and participants were recruited using the online platform Prolific (www.prolific.co). All Demographic data, including age and sex, were provided by Prolific. The initial data collection (one form of all tasks) was conducted over 3-4 days and took participants on average 193 min to complete. 298 participants started the first day of the experiment. Out of them, a total number of *N* = 257 (131 female, 120 male, 6 not stated, mean age: 29.8 ± 7.7) finished all tasks of the first experimental day and were included in our analyses. Of those, 244 completed the full 3-day battery. Participants were paid at a rate of $9.50/h, plus an additional $9 if they finished all the tasks within the week. Eight months after successfully completing the full battery (see below for exclusion criteria per task), we invited participants back to increase the power of our analysis and investigate reliability. At this stage we introduced two additional tasks to the battery—the Vanderbilt Expertise Task (birds, leaves and planes subscales) and a visual N-Back task with fractal stimuli, which included 0-back, 1-back and 2-back conditions. A total of *N* = 183 returned and did some portion of the tasks (see Table 1 for counts of participants per task), consisting of an additional 185 min of data collection on average. 89 participants successfully completed at least one form of all tasks. Of these, 41 participants successfully completed all forms.

To address the question of the number of timepoints needed to average over to achieve a stable reliability estimate, we used data collected for a separate longitudinal experiment focused on the FMP task. A more complete analysis of this dataset will be published separately. In this experiment, six different FMP forms were administered across six sessions with the following temporal design: days 1 and 2, 3 and 4, 5 and 6 were 24-72 h apart; days 2 and 3 were two weeks apart and days 4 and 5 were four weeks apart. 271 participants successfully finished the first day out of which 206 (76 female, 129 male, 1 not stated, mean age: 25.9 ± 8.2) successfully completed the whole six days. Participants were compensated at a rate of £8/h, plus an additional £10 if they finished all six forms (distributed as £2, £4, £4 bonus after finishing two, four, and all six forms respectively).

This study was approved by Weizmann and UCLA institutional review boards.

### Inclusion/exclusion criteria

We chose to exclude participants on a per-task basis, based on a combination of their accuracy, reaction time (RT) and individual trial responses. We assessed the following criteria and excluded participants from a task if two or more of the following criteria were met:

- Average RT was 2 standard deviations (SD) faster than the group mean.
- Standard deviation of RT was less than 2 SD below the standard deviation of the group.
- Average sequence length of a single response (i.e., repeatedly indicating the same response across consecutive trials) was 2 SD greater than the group mean sequence length.

If a participant’s accuracy was greater than 0.5 SD below the mean, they were included, regardless of their RT and individual trial responses. The only exception to this was for the MST, where if the standard deviation of the RT was less than 2 SD below the SD of the group, they were excluded regardless of performance, as this pattern of responses suggested that the task was being performed by a script/bot rather than a human.

After excluding participants based on accuracy, RT and individual trial responses, we additionally excluded participants based on the following criteria, which would indicate that they were not paying attention during the tasks:

- CFMT: Four or more incorrect trials in Stage 1 (i.e., Stage 1 score less than 83%). For participants with three incorrect trials in Stage 1, data were excluded if performance on the other stages indicated a lack of attention rather than a valid measure of poor performance.
- FMP: Accuracy below chance (50%) in Face-Matching trials.
- PGNG: Accuracy less than 3 SD below the mean for the two target identification stages. If accuracy was less than 2 SD below the mean, performance on the rest of the task was evaluated to determine whether low accuracy was because of a genuine lower performance or lack of attention.
- N-Back: Accuracy less than 3 SD below the mean for the 1-back blocks. If accuracy was less than 2 SD below the mean, performance on the rest of the task was evaluated to determine whether low accuracy was because of a genuine lower performance or lack of attention.
- VET tasks: Incorrect or missing responses on 2 out of 3 catch trials.

When comparing test-retest reliability and split-halves reliability on pooled data, we implemented another exclusion criteria. To ensure that the effect is not driven by outliers, we removed all participants from the analyses whose difference in score between the two sessions was more than 2 SD from the mean differences. This allowed us to remove a small number of participants (3-12 participants, no more than 8% of the total sample size for each task) that were exceptionally good or bad on one day and not on the other – a pattern suggestive of inconsistent data quality. In the case of the longitudinal FMP dataset, we performed this exclusion on score differences between every comparison and removed the union of all excluded participants, in total 23 out of 206 participants were removed.

#### Reliability calculations

**Fig. 1:**
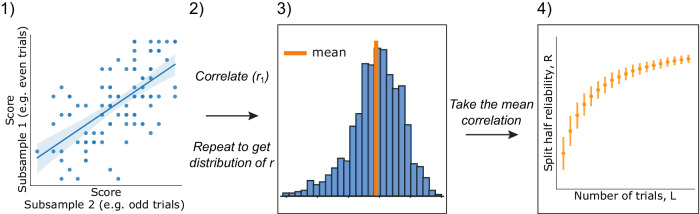
Reliability calculation. 1) For each of *N* participants, we take two random, non-overlapping samples of *L* trials (up to a maximum of half the total trials in a task) and then calculate the score for each sample, creating two vectors of length *N*. We compute the Pearson correlation of these vectors of subsampled scores across participants to get the correlation, *r*_*1*_. 2) We repeat this process 1000 times to get a distribution of correlations. 3) We take the mean of this distribution, *R*, to create one point in the *RL* plot. 4) We repeat for other values of *L* to create the full *RL* curve, errors are standard deviations of the distribution of reliabilities.

Figure 1 shows our methodology in calculating the reliability for different numbers of trials. We first split participants’ data into two smaller, non-overlapping batches of *L* trials, calculate the scores from those batches in each split per participant, and correlate these two measures across participants using Pearson’s correlation. This correlation is an indication of the consistency of the relationship between participants on this task when averaging across *L* trials to estimate performance per participant. We then repeat this calculation 1000 times to eliminate noise coming from a specific split selection, giving us a distribution of reliabilities for this behavioral measure at this sample size. We tested different numbers of samples ranging from 100 to 10,000 and chose 1,000 as that was sufficient for stable results without being too computationally costly. The mean of this distribution, which is equivalent to Cronbach’s alpha^84–86^, is used to create reliability vs number of trials (*RL*) curves for each task. Those curves are theoretically bound between -1 (perfect negative correlation) and 1 (perfect correlation), where a reliability of 0 describes a random dataset with no internal consistency in the order of participants’ scores across different subsamples of data (for instance, a participant might do very well in one subset, and poorly in a different subset), while a reliability of 1 denotes a perfectly reliable dataset where the relationship between participants is consistently maintained across all data subsets.

When comparing test-retest reliability between sessions to estimate the effect of time on reliability, we sample *L* non-overlapping trials per participant from session 1 and another *L* non-overlapping trials per participant from session 2, compute the measure of interest and calculate Pearson correlation *R* between these two vectors, between the two sessions. To test whether differences between the test-retest and split-halves curves were significant, we carried out simulations which randomly split trials into two groups for a permutation test, see Supplementary Methods for further details. We further removed two tasks (GFMT and Car Matching) in which there was a significant difference between the means of the first and second session, making them difficult to directly compare in the test-retest analysis. To test the difference, we employed a two-tailed *t* test, with t_300_ = 3.12, *p* = 0.00199, Cohen’s d = 0.36, CI = [0.02, 0.07], for GFMT and t_326_ = –3.16, *p* = 0.0017, Cohen’s d = 0.35, CI = [–0.07, –0.02] for Car Matching (see section Comparing test-retest reliability with split-halves reliability in SI Methods for all the comparisons).

In order to determine whether random data would show spurious but reliable correlations given a large enough number of trials, we simulated a set of random data. We randomly generated zeros and ones for *N* = 100 participants with *L* = 250 trials, and calculated reliability as we did for the real data. As different simulations gave reliability values that ranged between –0.1 and 0.1, we tested whether there was any consistent reliability in the random data by repeating the simulation 100 times and averaging the reliability curves. We then used a student’s *t* test to test whether this averaged reliability curve was significantly different from zero, and it was not (two-tailed *t* test, t_99_ = 1.17, *p* = 0.25, Cohen’s d = 0.12, CI = [–0.01, 0.02]).

**Fig. 2:**
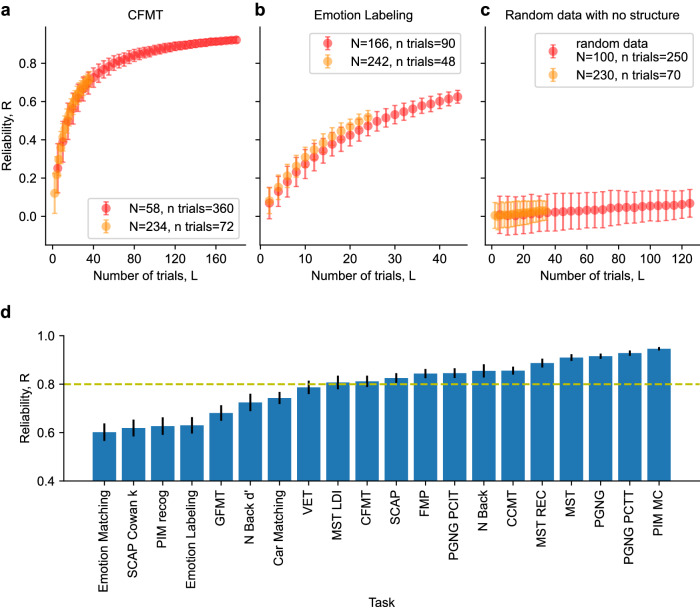
Dataset reliability. **a–c** *RL* reliability curves showing convergence for selected tasks as a function of number of trials. Since only a subset of the participants completed all versions of the tasks, we show here both samples with a larger *N* and fewer trials *L* (light orange), as well as smaller *N* with larger *L* (light red). **a** Cambridge Face Memory Test (CFMT); (**b**) Emotion Labeling task; (**c**) randomly generated data showing no internal consistency. **d** Barplot of reliabilities of one full form calculated using permutation based split-half reliability using all trials across two forms. Dotted line represents *R* = 0.8 (common benchmark of good reliability). SCAP Spatial Working Memory Capacity, PIM Personal Identity Memory, GFMT Glasgow Face Matching Task, VET Vanderbilt Expertise Test, MST Mnemonic Similarity Task, CFMT Cambridge Face Memory Task, FMP Face Memory Perception task, PGNG Parametric Go-No Go, CCMT Cambridge Car Memory Task. See “Methods” for an explanation of the different measures. The error bars in all (**a**–**d**) are the SDs of the distribution of correlations per given *L*.

To calculate reliabilities for our battery of tasks (Fig. 2d), we used two forms of each task to get a reliability that corresponds to one commonly used form of each task. For tasks that indexed memory (i.e., where repeating the task with the same exact stimulus set would potentially create learning-related improvements due to additional exposures), the additional versions of the task used the same task structure but novel stimulus sets. Specifically, for the CFMT, we used the original form^2^, and as a second form, we used the Australian version^81^. For VET tasks, three subtests (planes, birds, leaves) were administered and trials were concatenated to create one full form. For tasks where repeating the same task would not create any learning-related improvement, such as the working memory tasks, the same task was administered twice.

#### Effect of reliability on correlations between tasks

**Fig. 3:**
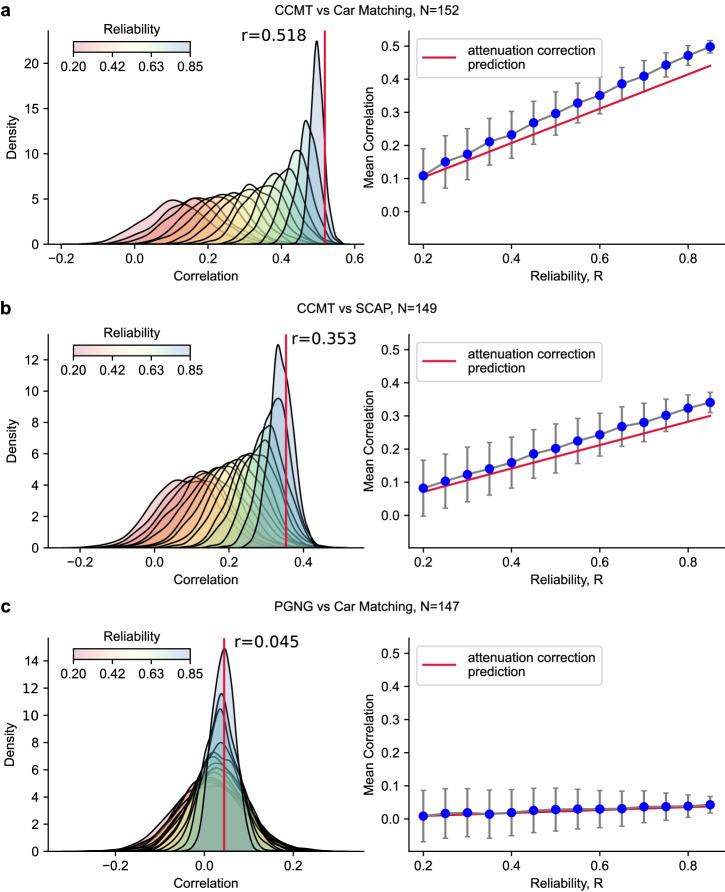
The effect of reliability on correlations between tasks. Left: distributions of correlations between the selected measures colored by resampled reliability of the measures. Red line denotes the correlation between the two measures calculated from the full sample of each. Right: mean correlation over all sub-samples between the two tasks for each reliability level. Error bars denote the standard deviation of correlation values across the different sub-samples. Red line denotes the correlation predicted by the attenuation correction formula. Note that unreliable data generally causes an underestimation of the true correlation values. **a** Correlation between two highly correlated tasks, the Cambridge Car Memory Test (CCMT) and Car Matching. **b** Correlation between two tasks with intermediate correlation, CCMT and Spatial Working Memory Capacity (SCAP). **c** Correlation between two poorly correlated tasks, Parametric Go-No Go Task (PGNG) and Car Matching.

For the analysis in Fig. 3, we used the following approach to estimate the effect of reliability on the correlation between two tasks. From our battery, we selected three pairs of measures. All measures used in the following analyses have high within-participant reliability of at least 0.85 for each measure separately (when using all available trials). The first pair, CCMT and Car Matching, has a high correlation between the two measures (*r* = 0.518, *p* = 7.99^–12^, CI = [0.39, 0.63]). The second pair, CCMT and SCAP overall accuracy, has an intermediate correlation between themselves (*r* = 0.353, *p* = 0.00001, CI = [0.2, 0.49]). The third pair, Car Matching accuracy and PGNG overall accuracy, has a low correlation between the two measures (*r* = 0.045, *p* = 0.59, CI = [–0.12, 0.21]). For each task, we calculated how many trials are needed to achieve different reliability values—from 0.1 to 0.85. In order to be able to utilize the full dataset for estimating the correlations, the number of trials per reliability level (per task) was calculated using the directly fitted fit method (see “Results”—derivation of the fit).

Having determined the necessary number of trials for each reliability level, we then subsampled this corresponding number of trials from each of the tasks and calculated the correlation between each of the two task pairs. We repeated this 1000 times to create a distribution of correlations between those two tasks for a given reliability level. The right panels of Fig. 3a, b display the mean and standard deviation of the correlation distribution for a given reliability (*R*).

#### Error estimation

**Fig. 4:**
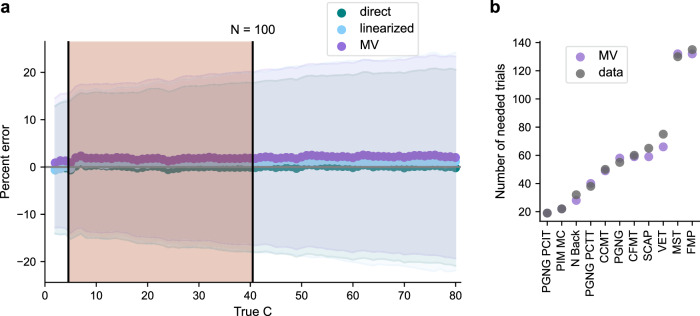
Comparison of three different methods for fitting and estimating the *C* coefficient. **a** Comparison of fitted *C* value to the true *C* value of simulated beta distributions (absolute difference). Each distribution was sampled 1000 times. Results are shown for *L* = 250 trials, *N* = 100 participants and for *C* in the range of 2–80. The plot shows error estimation (median and SD of percent error) of the different fitting methods for a range of values of the *C* coefficient (2–80). The shaded red region denotes *C* coefficient values observed in real-world data (*C* range: 4–41) and the unshaded region shows results from *C* values which we did not observe in our data (42–80). **b** The predicted number of trials to achieve reliability of 0.8 using the MV fit from Eq. (3) (purple) and an actual number of trials necessary for a reliability threshold of 0.8 as calculated from the data (black) for measures. Included are all measures that in our data reached reliability of at least 0.8.

To estimate error (Fig. 4a, b) for validating our method, we created synthetic data sampling *C* coefficients from 2 to 80 by 1 (79 values). We used a beta distribution that is defined using two parameters, $\alpha$ and $\beta$. We verified that all measures in our dataset can be well approximated and fitted using the beta distribution (Supplementary Fig. 1). It can be derived (see SI Sec. Calculating $C$ for the beta distribution) that $C=\alpha +\beta$ and we used $\alpha =\beta$ when generating beta distributions for simplicity. For a given value of *C*, the value of $\alpha$ would uniquely specify $\beta$, with a different $\alpha /\beta$ ratio. We tested that the ratio of these coefficients does not make a substantial difference when comparing the error of Eq. (3) (see Supplementary Fig. 2).

For each value of *C*, we ran 1000 simulations (realizations of each given combination of mean and standard deviation). When calculating reliability to obtain *RL* curves for directly fitted and linearized fit, we sampled *L* only 500 times to reduce computational cost. Each dataset contained 250 trials per participant for 20 different values of *N* (number of participants) ranging from 10 to 200 by 10 to create scaling of error with *N* (Supplementary Fig. 3). The ground truth for the *C* coefficient was established for each *C* separately by generating a distribution with large sample size of *N* = 10^7^ and using MV fit to get the *C* corresponding to this distribution. For all three fits, we computed the difference between the fitted *C* and this true *C*. We then divide this distance by the true *C* to get error in percent and report the median and standard deviation of this percent error (Fig. 4a, b).

To prevent artefactual error arising when the denominator of Eq. (3) approaches zero (happening for low values of *N* and *L*), we disregarded simulations where the denominator was less than 10^–3^. This threshold was determined by finding inflection points and maximas of second derivatives of the MV fit function. That yielded a range of thresholds from 3$\cdot$10^–3^ to 10^–3^ and the less stringent threshold was chosen as a compromise between the possible error and the probability of such an event (computational cost).

**Fig. 5:**
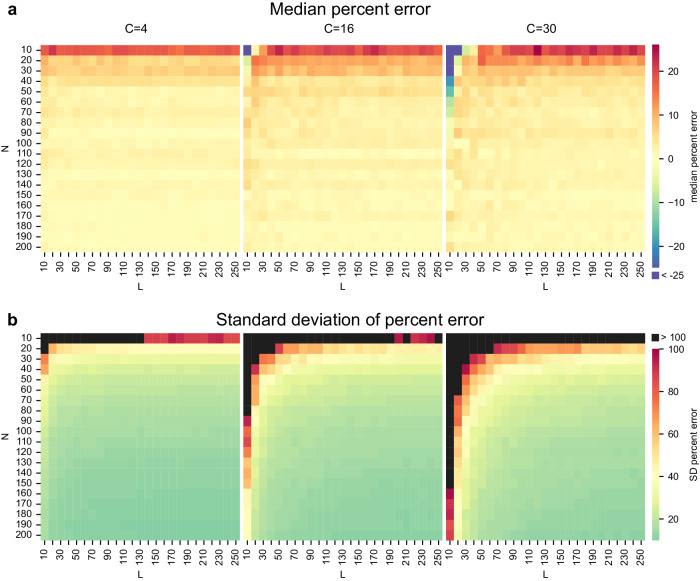
Estimation of error arising from limited sample sizes (number of participants *N* and number of trials *L* per participant) when using the MV formula for selected *C*s matching tasks with low, medium and high variance (PGNG PCIT, CFMT, and MST, respectively) across 1000 simulations. **a** Median percent error. Range is from -69% to 26% with values below -25% shown in dark blue. **b** The standard deviation of the error. Range is from 9.8 to 590% with values above 100% shown in black. Note that values over 100% only occur for small *L* and *N* (*L* < 30, *N* < 30).

The same approach with the same *C* values and policies was used to generate the error matrices in Fig. 5. The only difference is that each of these *N* and *C* combinations was created for 25 different *L* values ranging from 10 to 250 by 10 and only the MV fit was used. We compute the percent error as the median (Fig. 5a) and standard deviation (Fig. 5b) across the 1000 simulations of the distance of the fitted MV value from the true *C* value scaled by the true *C* value. This yields a *N*x*L* matrix of percent error for each *C*.

#### Online reliability tool

We created the online reliability tool (https://jankawis.github.io/reliability-web-app/) based on the MV fit. The tool provides confidence intervals calculated based on the simulations as shown in Fig. 5 and described above in the Error estimation section.

This tool can only be used for binary data. We recommend using at least 30, preferably 50 participants with at least 30 trials per participant. For tasks that are not binary, the *C* coefficient and the reliability curves can still be computed using the direct and linearized fits.

Detailed instructions on how to use the tool are provided in Supplementary Note in the SI and in the FAQs that are part of the GitHub repository (https://github.com/jankaWIS/reliability-paper).

### Reporting summary

Further information on research design is available in the Nature Portfolio Reporting Summary linked to this article.

## Results

### Measuring reliability

In the study of individual differences, it is very common to calculate correlations between two different measures across participants. For instance, in brain-behavior correlations, the main methodology is to search for correlations between the neural variance across participants and the behavioral variance across those same participants. For this metric to be meaningful, both the neural and the behavioral data need to be reliable in the sense that participants’ ranking within the cohort is as consistent as possible, as it is this internal consistency which drives the brain-behavior correlations. This is also the case when we seek to measure correlations of individuals across different behavioral tasks, or the stability of any measure in general. Throughout this paper, we estimate reliability (*R*) for our sample as the mean Pearson’s correlation across participants on different subsets of data from a given behavioral measure. To calculate this measure of reliability, we use a permutation-based split-halves extension of the classic test-retest reliability measure (see Methods and Fig. 1)^84,85,87–89^ as this method has previously been suggested to be optimal for calculating the internal consistency of cognitive tasks^44,87,88,90–92^. Note that for this analysis, we pool across all trials regardless of whether they were collected in the same session or not. Later analyses specifically test the effect of the session in which data were collected, see below.

We began by testing the reliability of a large battery of 12 commonly used cognitive tasks, as well as two new tasks which we developed for the purpose of studying personal identity memory (PIM) and face memory/perception (FMP). These tasks span 21 different behavioral measures in total, with some tasks including multiple measures which capture different aspects of behavior (Table 1, Supplementary Fig. 4, Methods). In order to test how reliability behaves across a diverse set of measures, we included several commonly used measures for these tasks. All data were collected online and were rigorously cleaned to detect and remove participants who were not paying adequate attention to the tasks (see Methods).

If the assumption of convergence to a stable mean holds, then averaging over more trials will provide a better estimate of participants’ true, stable trait-like proficiency, and our data will become more reliable. The rate of convergence is an indication of the degree of noise around each participant’s stable mean. Figure 2a–c shows examples of a reliable task, a less reliable task, and simulated random data, respectively (see Supplementary Fig. 4 for reliability curves of all 21 measures). Note that for the random data, reliability converged to a spurious value, which across hundreds of simulations was bounded between -0.1 and 0.1, the average of which was not significantly different from zero (two-tailed *t* test, t_99_ = 1.17, *p* = 0.25, Cohen’s d = 0.12, CI = [–0.01, 0.02]). Small residual correlations in the random data might be a result of bias in the pseudo-random seed used to generate the data, or of the limited sample size (see Methods)^93^. Data collection included at least twice as much data for almost all tasks than is commonly used (using alternate forms with different stimuli), allowing us to calculate the reliability of a standard one-form administration of the majority of our tasks (Fig. 2d).

### Effect of reliability on correlation between tasks

To test how internal consistency reliability affects correlations between two different measures in a real-world dataset (such as correlations between brain and behavior, or two different behavioral tasks), we examined different subsamples of our measures, considering three different scenarios: (A) two highly correlated measures, for which both measures had enough data to achieve high reliability, (B) two measures with intermediate correlation, and (C) two measures which were not well correlated with each other, but which both have high reliability (see Supplementary Fig. 5 for correlations between all measures). For each of these scenarios, we subsampled each of the tasks to several different reliability levels, and then calculated the correlation between the two tasks. This subsampling procedure was repeated 1000 times to create distributions of possible correlations at a given reliability level (Fig. 3).

Importantly, low reliability tends to cause an underestimation of the true correlation values. This is in line with the well described attenuation effect first put forward by Spearman^94–96^, in which the mean value of the observed correlation is lower than the true underlying correlation, as our data in Fig. 3a, b demonstrates. The left panels of the figure show the distributions of observed correlations between the two measures across all subsamples. The correlation between the full dataset of both tasks is shown by the red line. Note that when observing high correlation values $(| {{{\rm{r}}}} | \, > \, 0.4)$, this correlation is unlikely to be spurious (highly overestimated). However, when the true correlation is very low, as in Fig. 3c, observed correlations on a single “snapshot” of an experiment (i.e., one iteration of the split-halves analysis) can both underestimate or overestimate the true correlations.

The right panels show the same data, with each point depicting the mean of the histogram on the left for the given reliability level, and error bars denoting 1 SD of the distribution (blue lines). The red line plots the correlation predicted by Spearman’s attenuation prediction formula for different reliability levels, applied to the correlation value between the full datasets of both tasks (denoted by the red line in the plots on the left). Even though the full dataset is more reliable than the highest level of subsampling we reach, it is a single measure, and as such also appears to underestimate the true correlation when that correlation is high, accounting for the lower values of the red line in panels Fig. 3a and b but not in panel c.

As reliability of the tasks increases, the distribution of observed correlations between them becomes narrower, as evidenced by the decreased error across the different iterations shown both in the width of the distributions in the left plots, and as error bars in the right plots. The inherent problem with the subsampling method is that with an increased number of trials *L*, we sample a higher percentage of the dataset and thus reduce variance across the distribution of different subsampling iterations. Additional simulations using 10 forms of synthetic data and varying levels of constraining show that the reduced error is not solely driven by the subsampling method as is evidenced by the decreased variance as a function of reliability even for the less constrained data (i.e., when *L* is much smaller than the size of the dataset, see Supplementary Fig. 6).

### Derivation of the fits

Our next question was whether we could mathematically describe the convergence rate of the reliability based on the statistics of the distribution of performance on a given measure, for the general split-halves analysis. This would allow us to not only calculate reliability for a given dataset and to predict reliability for any given sample size, but would also provide us with a quantification of the convergence rate, an important factor in assessing the suitability of a measure for use in studies of individual differences.

Assuming there is no learning (i.e., samples are independent, and two consecutive trials are independent), and assuming each participant has a true proficiency (i.e., a true ability level for a given task), we would expect participants to each converge to a stable mean reflecting this proficiency. In this case, as we average across more trials, reliability at the individual level will increase, which will drive an increase in reliability across individuals (as is indeed shown in Fig. 2 and Supplementary Fig. 4). Under these assumptions, one can derive a formula describing the convergence curve (see SI for the exact derivation):

1 $$R=\frac{L}{L+C}$$

where *R* denotes reliability as defined above, $L$ denotes the number of trials used to calculate the reliability and the convergence coefficient *C* is a free parameter, which is fitted to the particular dataset. The maximum sample size is half of the trials, so *L* is always less than or equal to half of the number of trials in the given experiment. Note that this is a reformulation of the classic Spearman-Brown “prophecy” formula^94,97^, which similarly allows the estimation of reliability for larger number of trials based on previously calculated reliability for smaller number of trials^44,87^. This relationship between number of trials and reliability, however, not only gives a direct quantification of *C*, but allows a much easier calculation of reliability for any given number of trials.

To further describe the *C* coefficient, which in classical test theory represents single trial error variance over true-score variance (see SI derivations for details), and to allow for an even more straightforward calculation of reliability as a function of number of trials in a given task, we sought to describe *C* purely from the statistics of the distribution for that task, without fitting the reliability curve. This is only possible for tasks with binary outcomes on individual trials (e.g., 1/0, correct/incorrect). In these cases, *C* can be expressed using the statistics of proficiencies $f(P)$:

2 $$C=\frac{{\mathbb{E}}\left[P\right]-{{\mathbb{E}}\left[P\right]}^{2}}{{{{{{\rm{Var}}}}}}\left(P\right)}-1 ,$$

where Var$(P)$ denotes the population variance the distribution of proficiencies $P$ and ${\mathbb{E}} [P]$ denotes its mean. In the real world, however, we only have access to the variance of our limited sample and not the true variance of the population. We discuss the impact of limited numbers of participants below. Likewise, for a given participant, we do not have access to their true proficiency, but rather to the proficiency estimated from a limited number of trials. To account for the sampling error which arises from the limited nature of our sampling per participant, we use the law of total variance^98^. We define a random variable $Z$ that is an estimate of the participant’s proficiency, leading to the following corrected formula (see SI for derivation):

3 $$C=\frac{{\mathbb{E}}\left[Z\right]-{{\mathbb{E}}\left[Z\right]}^{2}-{{{{{\rm{Var}}}}}}(Z)}{{{{{{\rm{Var}}}}}}\left(Z\right)-\frac{{\mathbb{E}}\left[Z\right]-{{\mathbb{E}}\left[Z\right]}^{2}}{2L}}$$

The naive estimation of the *C* coefficient using Eq. (2) leads to a systematic bias which consistently underestimates the *C* coefficient by a large margin (see Supplementary Fig. 7). This bias is entirely corrected even for a small number of trials (*L* > 20) using Eq. (3), though a marginal overestimation following the correction remains (Fig. 4, Supplementary Fig. 7).

The *C* coefficient is inversely proportional to the variance in performance on the task across participants – the smaller the variance, the higher the *C*, and the higher the number of trials (*L*) that are necessary to reach a given reliability level (*R*). The *C* coefficient can be fitted from the data using the following three methods (see Supplementary Fig. 7):

1. Directly fitted: fit the convergence curve with hyperbolic function from Eq. (1). Note that this is different from solving the equation for a single given *L* and *R*. To account for noise from a single measurement of *R* (which is reflected in the error bars in Fig. 2a–c) it is advised to fit the full *RL* curve to get *C*.
2. Linearized: fit $1/R$ vs $1/L$ curve, giving a linear fit with an intercept that should be equal to 1 (if trials are independent, i.e., no learning, no fatigue), analogous to Gulliksen^87^:
  4 $$\frac{1}{R}=1+\frac{C}{L}.$$
3. Mean/Variance (MV): for binary tasks, fit *C* using the mean and the variance of the underlying $f$ distribution using Eq. (3). Note that the first two methods are general and can be used for any dataset, whereas this method is limited to binary tasks, meaning tasks in which the outcome of any given trial is binary and there are only two possible outcomes (0/1, correct/incorrect, blue/red, etc.). Multiple choice tasks with more than two alternatives can be used, as long as the outcome of the trial is binary (e.g., tasks with 4 options, 1 correct and 3 incorrect).

### Validation of the different fits

To compare the different fits and how well they are able to predict reliability, we ran extensive simulations of synthetic data mimicking real behavioral data from our task battery to supplement our analysis of the large real-world dataset we collected. We sampled beta distributions as they are naturally bounded between 0 and 1 and because tasks in our dataset were well approximated by a beta distribution (see Supplementary Fig. 1). We created a dense sampling of *C* values in the range observed in our real-world dataset, and also extended this range further to expand our sampling of the space of potential *C* coefficients. For each distribution, we ran 1000 simulations of an experiment with the same overall *C* (as if participants took the test many times) for several values of *N* (number of participants). We then compared the fitted *C* value from the simulated data to the true *C* value of the distribution from which the data was generated (see SI for the derivation of the *C* coefficient for those distributions). Analyses for both the real-world and the simulated datasets are shown in Fig. 4.

As seen from the plots, all three fitting methods (directly fitted, linearized, MV) yield very similar results, and similar (low) error rates (Fig. 4, Supplementary Fig. 7). See Supplementary Fig. 8 for an analysis of the intercept of the linearized fit across our real-world datasets, which validates our assumption that there was no significant learning in the tasks included in our dataset. Median percent error was not significantly dependent on the value of *C* (distribution of differences between neighboring points was not significantly different from 0, two-sided *t* tests: t_direct, 77_ = 0.23, p_direct_ = 0.82, Cohen’s d_direct_ = 0.026, CI = [–0.04, 0.05]; t_lin, 77_ = 0.82, p_lin_ = 0.42, Cohen’s d_lin_ = 0.093, CI = [-0.03, 0.08]; t_MV, 77_ = 0.59, p_MV_ = 0.56, Cohen’s d_MV_ = 0.067, CI = [–0.03, 0.06]), though the degree of uncertainty in the simulations (SD of the error), did grow as a function of *C* (Fig. 4a). Predictions of the number of trials necessary for achieving 0.8 reliability using the MV fit compared to reliability computed directly from the data show that this error is very minimal even when translated to absolute number of trials (Fig. 4b).

### Dependence of the fits on the number of trials and sample size

To further investigate how a limited number of trials and/or participants might affect the accuracy of our estimate of reliability, we ran 1000 simulations of an experiment with the same range of *C* coefficients as those observed in our real-world data, and varied both the number of participants (*N*) and number of trials (*L*) (Fig. 5). As before for the fixed *N* and *L*, median percent error estimated by the MV fit was largely stable across different *C* coefficients, as shown in Fig. 5a. The three values of *C* shown in this plot are representative of values observed in our real-world data: low *C* (left), corresponding to high variance between participants (e.g., Parametric Go-No go Task, percent correct to inhibitory trials), middle range *C*, i.e., middle range variance between participants (middle, e.g., Cambridge Face Memory Test), and high *C*, corresponding to low variance between participants (right, e.g., Mnemonic Similarity Task). As can be seen from all three heatmaps, the median error is negligible and close to zero for $N \ge 50$ and $N \ge 30$, and the decrease in the standard deviation of the error, while higher than the median error, also falls off sharply beyond this point (see also Supplementary Fig. 9 for curves showing the decrease of the SD of the error with *N* and *L*). While Fig. 5a shows the median error across our simulations, Fig. 5b shows the SD of the error calculated across the different simulations. If both the number of trials and the number of participants is very low, error rates are higher, and this is compounded for large values of *C* (reflecting low variance in the data). Because Eq. (3) (which defines the MV fit) is derived for large *N*, it can reach a point of singularity for very small samples (low *L* and *N*), making it impossible to calculate *C*. We remove such rare extreme cases from our simulations (see Methods for details).

### Reliability of real-world tasks

**Fig. 6:**
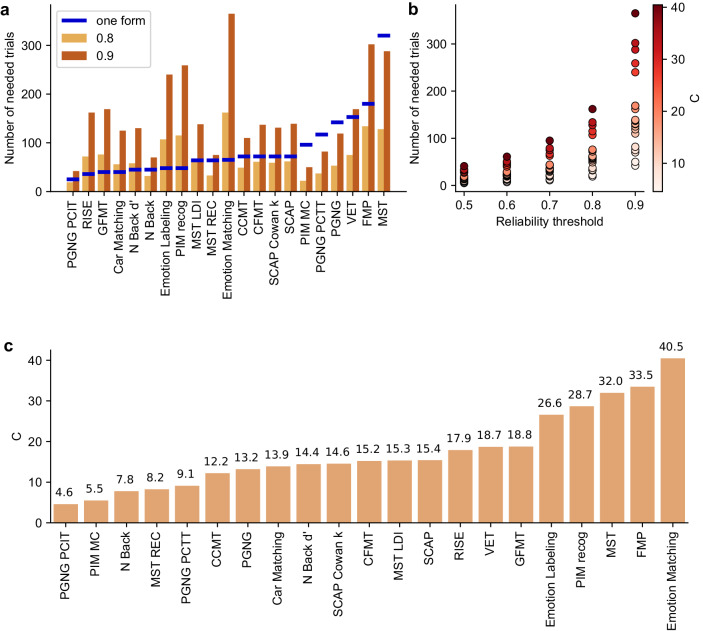
Reliability and its behavior in the real world. **a** Number of trials needed to achieve given reliability (0.8 in light brown, 0.9 in dark brown) per task. Blue marks denote the number of trials in a single standard form of each task. Tasks where the light brown bar is lower than the blue mark are those where one form shows reliability over 0.8. Predictions were obtained from direct fits of the reliability curves. Note that for some tasks, multiple measures are shown illustrating different aspects of performance. **b** Dependence of the necessary number of trials to reach different reliability levels in our real-world dataset, colored by the value of the *C* coefficient. Note the non-linear increase in the number of trials needed to reach higher reliabilities, a visual representation of the prediction of Eq. (1). **c** Value of *C* coefficient across tasks, calculated from the direct fit. See Methods for an explanation of the different measures.

Having established our ability to accurately fit reliability curves to our data, allowing us to predict the reliability for any given sample size, we can now look at the split-halves reliability and the projected reliability for larger sample sizes of all the tasks in our battery. Figure 6a shows the number of trials necessary to reach reliability thresholds of 0.8 and 0.9 per task. For analyses which involve correlating different measures, we would recommend 0.8 as a reasonable minimal reliability to aim for, given the effect of reliability on observed correlations (Fig. 3). The blue line indicates how many trials there are in the standard administration of each task. As can be seen, while some tasks have enough trials to reach a reliability of 0.8 (blue line above the light brown bar), some are considerably less reliable, and very few have enough trials to reach a reliability of 0.9 (blue line above the dark brown bar).

Figure 6b shows the dependence of the number of trials on both the value of *C* and the desired reliability level. There is a non-linear increase in the number of trials necessary for higher reliability if the variance of the task is low (high *C* values), as predicted by Eq. (1). Figure 6c orders the tasks based on their value of *C*. Note that different measures of the same task can have different values of *C* which would influence their reliability values, even for the same number of trials (e.g., MST REC and MST LDI). For other tasks, different measures might be calculated based on different numbers of trials (e.g., PGNG PCIT and PGNG PCTT). In such cases, it will be difficult to know which measure is more suited for studies of individual differences by simply directly comparing reliability, whereas comparing *C* coefficients will give a clear answer.

### Effect of time on reliability

When two measures are compared, they often come from separate days, whether those measures reflect scanning sessions or diagnoses obtained at a different time point. It is conventionally assumed that the change in participants’ behavior during this period is negligible. In our previous analyses, we disregarded the effect of time, pooling data from different days and treating trials collected across different time points as equivalent. Yet there are several potential sources for change in performance across measurements, from transient change arising from momentary differences in attention/arousal across different time points, to true change in cognitive abilities across longer time scales. Moreover, different tasks measuring different cognitive domains might be differentially affected by both transient and more long-term changes. To test this, we examined test-retest reliability, which is a special instance of split-halves reliability, in which trials are only subsampled within one specific testing time point, and then compared to trials subsampled from a different testing time point. Any changes in performance within participants across testing times will result in a reduction in reliability of the test-retest measure as participants’ performance will be less correlated to itself, and will also lead to the reliability curve not converging to 1 for infinite trials, as this is a violation of the assumption that the mean is stable (per participant) across different splits of the data.

**Fig. 7:**
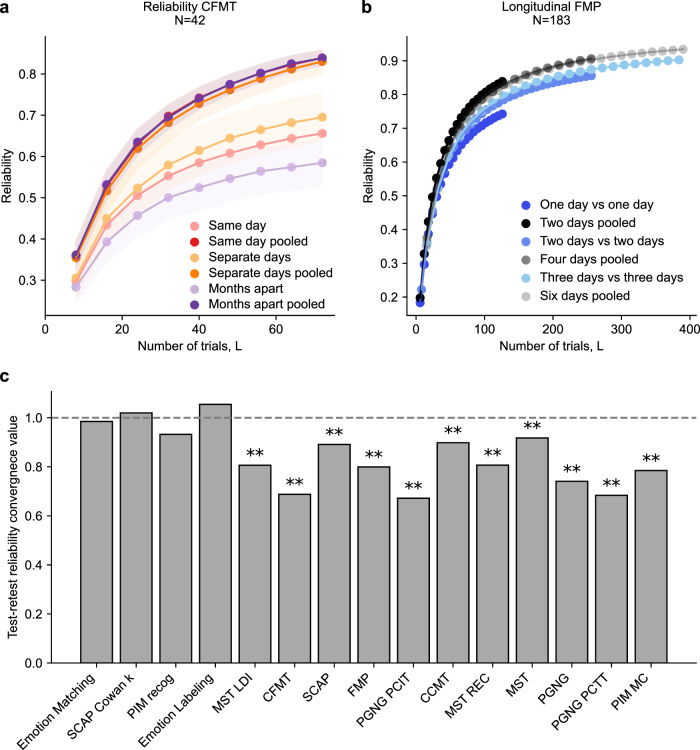
The effect of time on reliability. **a** Comparison of test-retest reliability (light shades) and split-halves reliability on pooled data (dark shades) of CFMT versions performed on the same day, separate days and 7-8 months apart. Error shading denotes the standard deviation calculated across different subsamples of *N* for the two groups with larger number of participants (separate days, months apart). **b** Reliability of FMP across multiple sessions. Split-halves reliability when pooling across two, four, and six testing days (black, gray curves) is compared to test-retest reliability either calculated between two different testing days, or between four or six different days, averaging across two or three non-overlapping days respectively. All statistics are based on permutation tests, see Methods. **c** Test-retest reliability convergence value for infinite number of trials (based on attenuation correction formula which computes the ratio of within day vs. across day reliability) and comparison of split-halves and test-retest reliabilities for measures in tasks where the two forms were completed a few months apart and without any significant difference between the means of the two sessions (all tasks excluding GFMT and Car matching, see Methods and Supplementary Table 1). Dashed gray line denotes value of 1 which the split-halves analysis in our dataset converges to within a small error margin (mean = 1.001, SD = 0.002). Stars denote the significance of the difference between the split-halves and the test-retest analyses: *p* < 0.01 (**). Significance and *p*-values determined by permutation tests, see Supplementary Methods for details: PIM recog *p* = 0.172, Emotion Labeling *p* = 0.230, Emotion Matching *p* = 0.402, SCAP Cowan’s k *p* = 0.388. All the other tasks were entirely outside of the random distribution of 1000 permutations (*p* < 0.001).

Our dataset allows us to examine two distinct session types: those with minimal temporal distance (on the same day or few days apart) and those separated by a significant amount of time (months). To test the effects of different time scales, we examine the CFMT, for which a batch of participants (*N* = 42) completed two different CFMT forms on the same day, while another batch of non-overlapping participants (*N* = 77) performed the tasks a few days apart. A third group of non-overlapping participants (*N* = 79) completed the two forms 7–8 months apart. The results are shown in Fig. 7a. To control for the different numbers of participants (*N*), we downsampled *N* in all groups to match *N* in the group with the fewest participants (same day, *N* = 42). For each time scale (within day, a few days apart, a few months apart), the test-retest analysis (light shades) is compared to the split-halves analysis which pools all data across the two forms, and then randomly splits them disregarding time (dark shades). In theory, if the sessions are equivalent, test-retest is a single example of a split in the pooled data, so we should see no difference in reliability between pooled and test-retest reliability curves. We find a significant decrease in reliability both between the split-halves data and all three test-retest curves (based on comparing the pooled and the test-retest reliability distributions for *L* = 40; same days two-tailed *t* test, t_1998_ = –62.85, *p* = 0.0, Cohen’s d = 2.81, CI = [–0.16, –0.15]; separate days two-tailed *t* test, t_1998_ = –53.61, *p* = 0.0, Cohen’s d = 2.40, CI = [–0.12, –0.11]; month apart two-tailed *t* test, t_1998_ = –105.79, *p* = 0.0, Cohen’s d = 4.73, CI = [–0.21, –0.2]), as well as between the three test-retest curves themselves (same day vs separate days, same day vs months apart, and separate days vs months apart). These differences in reliability are all significant (based on comparing distributions of expected test-retest reliability tested by ANOVA, F(2, 2997)  =  1071.89; *p*  <  0.001; MSE  =  6.16; $\eta {p}^{2}$  =  0.42, and post-hoc Tukey test: same day vs months apart, T = 29.01, *p* < 0.001, Cohen’s d = 1.30, CI = [0.0904, 0.1063]; separate days vs months apart, T = 45.76, *p* < 0.001, Cohen’s d = 2.05, CI = [0.1472, 0.1631]; separate days vs same day, T = 16.74, *p* < 0.001, Cohen’s d = 0.75, CI = [0.0488, 0.0647], see Supplementary Methods). Note that reliability is higher between two sessions a few days apart compared with two sessions on the same day, perhaps indicating the greater effect of fatigue when measuring the beginning and the end of the same testing day.

Next, to test whether test-retest reliability within a short timescale (weeks) converges to 1 when data from different days is averaged, we utilized an unpublished dataset from a separate study of the FMP task, which included six sessions per participant with alternate forms on each session. This allowed us to contrast test-retest reliability calculated within just one single day compared to one different day, to data averaged over 2–3 days and compared to data from 2 to 3 different days. Figure 7b shows how averaging over even just 2–3 days increases test-retest reliability.

Finally, we examined data from all tasks, to investigate whether they are all impacted the same by changes in performance across days. Here, day 1 and day 2 of data collection were always months apart. Using the same attenuation correction formula as above, we can estimate the point to which the curve would converge given infinite trials, which represents the upper bound of test-retest reliability. The attenuation correction formula computes the ratio between the geometric mean of the reliability (correlation) within each day vs. the correlation across days (see Supplementary methods). A value lower than 1 means that the correlation across days is lower than the correlation within days. This is shown in Fig. 7c (see Supplementary Fig. 10 for comparison of the full split-halves and test-retest reliability curves and Supplementary Fig. 11 for scatter plots). Comparing the convergence point of the test-retest measure to the convergence point of the split-halves analysis on the pooled data (the latter always being very close to 1 with mean across tasks = 1.001, SD across tasks = 0.002), it is clear that the effect of measuring performance at different time points varied greatly between tasks. Some tasks were not significantly affected (e.g., Emotion Labeling and Emotion Matching, significance determined by permutation tests, see Supplementary Methods for statistical details), while other tasks were heavily impacted (e.g., PGNG or SCAP).

### Application of the MV fit for easy study design

Important parameters in study design include the number of trials to be collected and the number of participants needed in order to obtain reliable results at the individual level, especially if the intended use is to investigate individual differences. This is true both for the design of new tasks and for commonly used tasks, which may not have been previously validated for high reliability at the individual level, as was the case for many of the tasks in our battery. In order to provide the community with a tool for building reliable studies, we have developed a simple web application using the MV fit based on our results above. To use this app, a researcher could run an initial pilot study using a small number of trials, which will be used to estimate the mean and sample variance (across participants) of the population. These numbers, along with the number of participants on which they were calculated, can then be entered into the app, which will output an estimate of the necessary number of trials to reach any given level of reliability.

To account for inaccuracies due to low sampling of the task’s distribution resulting from small sample sizes, we implemented the error estimated from our extensive simulations (Figs. 4a, 5) in the app, as confidence intervals.

The tool is available online and its basic functionality is depicted in Supplementary Fig. 12 (see SI Note for use instructions and link to the website). The code repository on Github contains FAQs about this tool.

## Discussion

As the focus in psychology and cognitive neuroscience research in recent years has increasingly emphasized understanding the nature of individual differences, it has become crucial to better validate behavioral measures to ensure high reliability at the level of the individual. A common approach adopted in many studies is to use individual differences in behavioral measures to search for the neural variance which explains them, i.e., brain-behavior correlations. In many of these studies, behavioral measures are collected separately from the neural data, often at a different time point, for many good practical and technical reasons. Since the behavior-neural comparison in such cases assumes that the behavior has not changed in the interval, the trait-like stability of the behavioral scores at the individual level is likewise critical for such analyses. The same is true of many other fields utilizing individual differences studies. Yet the very existence of such stable, trait-like individual performance across days has mostly been assumed rather than directly demonstrated in many commonly used tasks. Moreover, many cognitive measures were designed to detect group effects, where the reliability of scores per individual is not as important^58^. Averaging over many participants achieves the same effect of convergence to a stable group mean, even if individual reliability is low. Here we set out to directly test the assumption that relative individual performance on many different commonly used tasks will converge to a stable average given sufficient data, and to ask the more complex question of exactly how much data is sufficient and whether it is possible to estimate this value before full data collection, rather than after.

As was previously reported in the literature^51–57^, and can also be seen in our data (Figs. 2, 6), many tasks have only modest reliability at the individual level. The importance of high reliability for detecting true correlations has also been previously discussed, and the effects of low reliability have previously been shown in simulated data^42–44,47,87,99–103^. We demonstrate these effects on real-world data in Fig. 3 which shows how reliability affects the observed correlation between two different measures, grossly underestimating high correlations, and either underestimating or inflating very weak correlations. In this manuscript, we examine correlations between behavioral measures, but the same effect should be seen for low reliability on the correlation of any two measures, including brain-behavior correlations (see also Nikoladis et al.^43^ for a more in-depth discussion of the effect of behavioral reliability on brain-behavior correlations, which is mathematically similar to comparing two behavioral tasks). Our data are therefore reassuring in the context of the debate regarding inflated correlations in brain-behavior analyses, if moderately high correlations are observed (for instance Fig. 3b shows that a correlation of 0.35 is very unlikely to be overestimated).

There are many different definitions of reliability, which are used for different purposes. Traditional test-retest reliability involves administering the same task twice to the same group of participants, and then calculating the Pearson’s correlation between the two administrations. The repeated split-halves reliability which we use here to calculate reliability of pooled data is an expansion of the traditional test-retest reliability. Repeated split-halves reliability, which is focused on the internal consistency of the scores across participants, rather than the absolute scores themselves, is relevant for our aim of improving the reliability of individual differences measures. However, in cases where the aim is to validate the stability of the absolute score on each of the items on the test in order to provide population norms and statistics, subsampling of different trials within the task as we do here is not helpful, and a different calculation of reliability is used^101,104–106^. For a detailed discussion on these two divergent and often confusing definitions of reliability, see Snijder et al.^48^.

Such split-halves reliability ignores the effect of different measurement times, and treats all trials as being equivalent. Our comparison of split-halves with test-retest reliability, which differentiates between testing times, uncovers two more important insights which can be gained by examining reliability curves. First, while it would be generally expected for test-retest reliability to be lower than split-halves reliability on the pooled data^107^, we observe that the variability of this effect between tasks is substantial. Some tasks show no significant difference between these two methods while other tasks show a significant reduction in test-retest reliability compared with split-halves reliability (Fig. 7c, Supplementary Figs. 10, 11). Secondly, analysis of the CFMT data (Fig. 7a) shows that changes in tasks that are affected seem to be driven both by transient differences, as seen by the reduction in test-retest reliability when comparing forms completed on the same day or a few days apart, as well as potentially by true changes in ability which occur over longer time scales, as seen by the further reduction in test-retest reliability in forms completed months apart. Note that the analyses on the different time lags between tests were carried out on different groups of participants, so cannot be directly compared, and we cannot completely rule out that our effect is driven by differences between participants. Unfortunately, the rest of our dataset was collected with testing days months apart, so it was not possible to conduct this comparison on all tasks.

Interestingly, tasks in which performance was most affected by measuring across different days were tasks with strong memory or attention components, whereas tasks which were more perceptual in nature, or which measure traits thought to be more static, like working memory capacity (SCAP Cowan’s K), did not show differences in performance across days. These differences can be seen in the quantification of this effect of different measurement times (Fig. 7c). Importantly, analysis of the many sessions of the FMP data (Fig. 7b) suggests that at least for transient changes, averaging over 2–3 testing days is enough to achieve a stable measure of performance, which appears to approximate a true trait-like measure even when compared to different days. This recommendation is particularly meaningful if the aim is to compare two different measures, which may be taken at different time points. More research will be required to disentangle the effects of changes in performance over different time scales and their interactions with different tasks, similar to Bohn et al.^70^. One strong prediction from these data would be that averaging across days would remove the transient changes in reliability due to differences in attention or other transient states, but not differences in reliability due to baseline shifts in ability which might occur over longer time scales.

How can we determine which tasks are best suited for studies of individual differences? This has become an even more pressing issue with the advent of online testing, which has promoted the development of many new task paradigms. A tool which could both inform study design by quickly and easily predicting from small amounts of pilot data the necessary number of trials to achieve high reliability, as well as provide a straightforward evaluation and comparison between tasks of their efficiency for individual differences studies, would therefore be of great value to the community.

To address these challenges, we mathematically derived a simple equation which is a reformulation of the Spearman-Brown prophecy. Our equation introduces the convergence parameter *C*, that describes the rate of convergence of the reliability curve as a function of the number of trials for a given measure. *C* is inversely proportional to the variance in performance across participants—i.e., the lower the variance across participants, meaning the narrower the distribution of performance, the larger the value of *C*, and the more trials will be necessary in order to achieve reliable separation of individuals. Intuitively, tasks with very low variance in performance across the population, for instance, due to floor or ceiling effects, will need a large number of trials in order to rank participants reliably and maintain internal consistency. This relationship between *C* and the number of trials needed for increased reliability is non-linear, as is shown in Fig. 6b.

Our analysis is unique in that we not only validate our calculation of the *C* coefficient on simulated data, but also on a broad set of real-world behavioral datasets across many different tasks and cognitive domains. Crucially, all these data across all the different tasks were collected on the same participants, allowing us to directly compare the tasks (Table 1). Simulations are often the gold standard as they are not limited by expensive and time-consuming data collection, but they require making assumptions on the data which may not hold true for different tasks. Figure 4 shows that applying both the direct/linearized fit formulas as well as the MV fit very accurately predicted the reliability for different sample sizes across both our real-world dataset as well as across large simulated datasets. Figures 5c, 6 and Supplementary Fig. 4 show that different tasks had very different convergence rates, even when measured within the same individuals.

The remaining question is how much data are needed in order to get a reasonable estimate of reliability and of the *C* coefficient, for instance in the case of a pilot study. The accuracy of the estimate depends on both the quality of the data and the sample size. When collecting data online, it is recommended to first thoroughly clean the data, removing non-compliant participants and identifying participants who were inattentive to the tasks^108–110^. This is, naturally, also true for data collected in the lab. As for the effect of sample size on the potential error in the estimation of *C*, we ran extensive simulations to estimate the dependence of the error on both the number of participants (*N*) and the number of trials (*L*) (Figs. 4a, 5). A surprising result from this analysis is that beyond a relatively small number of participants (*N* ~ 40-50) and a small number of trials (*L* ≥ 30), the reliability estimates are very robust, and the error is not greatly reduced by increasing sample size (see also Supplementary Fig. 9). For a given number of trials, this is achieved in our formula through the implementation of the law of total variance, which corrects for the systematic underestimation of *C* which exists otherwise (Supplementary Fig. 7). Our results suggest that collecting data from 40-50 participants will provide a reasonable sampling of the statistics of the population for these tasks, and also serves as a general guide to selecting the minimum number of participants for an individual differences study.

One strong recommendation that emerges from our work is to focus on highly reliable tasks, and consider the tradeoff between data per participant and number of participants. By using highly reliable tasks and collecting more data per participant, ideally over 2–3 different testing days, experiments could preclude the need for thousands of participants. The other recommendation would be to focus on behavioral tasks and neural measures that do not have very low correlations between them (r < 0.1), unlike many of the brain-behavior correlations discussed by Marek et al.^30^. While not guaranteed, higher correlations could be accomplished by choosing tasks that are more domain-specific (and thus likely to better predict the connectivity strength between certain nodes), by using multivariate instead of univariate neuroimaging measures, or by functionally defining relevant regions individually instead of using generic anatomical parcellations.

Our reformulation of the Spearman-Brown prophecy, along with the derivation of *C* as a function of the mean and variance of the distribution, provides a simple way of calculating the reliability of any given dataset, and predicting how many trials will be needed to reach any given level of reliability. Perhaps more importantly, this reformulation suggests another way of conceptualizing reliability not only as an outcome to strive towards, but also as a core measurable property describing the suitability of a task for individual difference studies. The convergence parameter *C* provides us with a “score” of how well a task can reliably separate individuals, above and beyond the reliability of the standard administration of that task which could vary with number of trials included in the form (Figs. 4, 6). It is a direct assessment of the task itself as a measure of individual differences, not just of how well the standard administration does in collecting sufficient data in terms of having enough trials. This can help evaluate the suitability of that particular task for individual differences studies in relation to other similar tasks. The main utility of *C*, a dimensionless coefficient, is therefore primarily in comparing different tasks, rather than in the meaning of the absolute number in itself, which is difficult to interpret on its own. For instance, the standard administration of the Cambridge Face Memory Test (CFMT) is more reliable than the Glasgow Face Memory Test (GFMT), and also more suitable for studying individual differences (lower *C*). Emotion discrimination tasks (emotion labeling and emotion matching) have very low reliability for a standard administration. These tasks also have high values of *C*, perhaps pointing to a lack of objective answers for these tasks. We provide the range of *C*s calculated from our dataset as a starting point (Fig. 6c), to help relate new tasks to an existing, though partial, scale. We expect that as more data accumulates regarding values of *C* on different tasks, the absolute value in itself will become more informative. Note, however, that tasks can differ greatly not only in how many trials are necessary to reliably separate individuals but also in how long it takes to collect said number of trials, as time is a key practical parameter in real-world studies. Since the dependence of the number of trials on *C* is not linear, and trial length varies widely between tasks, there is no trivial comparison between two tasks with different *C* parameters in terms of the time taken to collect the data. Experimenters are therefore advised to factor in the time necessary to collect enough data for their desired reliability level.

Our results show that reliability of standard tests should not be implicitly assumed but rather experimenters need to explicitly ensure that the test instrument, together with experiment parameters including number of subjects and number of trials, collectively yield a study with acceptable reliability. The analytical methods we presented in this study can be adopted as a general tool for study design, and can be used both to project the necessary number of trials/participants for any desired reliability level, based on only a pilot study, as well as to characterize reliability of existing data sets (e.g., before subjecting them to brain-behavior association analyses). In order to maximize the accessibility of this tool, we created a simple web application that allows researchers to obtain the reliability curve and the *C* coefficient simply by entering the mean, sample variance, number of participants and trials in their study. The application also has the option to calculate the time it would take to collect enough trials for any given reliability level by including the amount of time taken to collect a given number of trials (Supplementary Fig. 12). The web app implements confidence margins, based on the mean and standard deviation of the error observed across our widespread simulations. This addresses the potential for a small error in the calculated *C* leading to a large error in projected number of trials, as discussed by Charter^86^. Our hope is that by reducing demands on the computational background of end-users to an absolute minimum, the web app will remove any barrier that would otherwise hinder the applicability of this finding and allow researchers across different fields to easily use this tool in their research to optimize study design.

Note that the only assumption we have made in deriving these fits is that there is no learning (e.g., performance changes due to stimulus familiarization or new cognitive strategies), or alternatively, fatigue over time as participants perform each task. To minimize fatigue, we split our tasks across several shorter testing days, providing participants with a financial bonus for completing all tasks across the different days in order to minimize attrition. Even so, some participants were missing some of the data for various reasons. The issue of missing data is complex, as the effect of missing trials in calculating reliability is task dependent (for instance, whether all trials are the same type, same difficulty level, etc.). Our analysis is based on large datasets with overall few missing data points, so we were able to ignore missing data. Participants with many missing data points were excluded based on our other exclusion criteria (see Methods). Different experimental setups might require different approaches to dealing with missing data^111–113^. To ensure that there is no learning, we used alternate forms whenever more than one form was collected (except for the CCMT, which shows no learning even with the same form, as is shown by the intercept for the linear fit, Supplementary Fig. 8). For most tasks, the use of alternate forms is important to counteract learning. It follows from the equation of the linearized fit (Eq. (4)) that if trials are independent (an assumption in our derivation which would translate to no learning or fatigue), the intercept of the fit must be 1, which is indeed the case for all or most of our measures (see Supplementary Fig. 8). Deviations from an intercept of 1 on the linearized fit suggest a violation of the assumptions (no learning / fatigue). This could provide a way of testing the presence or absence of learning or fatigue in a particular dataset, as well as opening up potential directions for future explorations of learning by developing further models to understand the interaction between learning and the intercept. Use of the *C* coefficient could also be extended to investigate the reliability of responses in research areas where intra-individual variance is a key measure of interest, such as in mind-wandering studies^114^, where fluctuations in performance index level of task-directed focus. Likewise in consciousness studies, individuals consciously aware and perceiving a certain stimulus may exhibit more reliable responses, potentially resulting in a lower *C* coefficient compared with those who do not consciously process the stimuli^115^.

### Limitations

Although this real-world dataset collected online represents a more heterogeneous population than the typical study conducted in-person in a lab (often overwhelmingly composed of college students), it is still likely less heterogeneous than the full general population. It is possible that more participants will be required to reach target reliability thresholds for the tasks we report here when studies include even more diverse populations and/or individuals with clinical disorders.

Although the derivation of Eqs. (1) and (4) assume only independence of trials (i.e., no learning or fatigue), the formula for the MV fit (Eqs. (2) and (3)) that is implemented in the app requires measures to be sampled from a Bernoulli distribution (i.e., each trial can yield only two possible outcomes). For any other measures which do not obey these assumptions (e.g., response times or scalar ratings), we suggest applying the directly fitted or linearized fits (not through the app). See also *What are the limitations of this app? Can I use it for any task?* section of the FAQ.

For some distributions, for which the calculated *C* is very high, corresponding to very low variance (potentially as a result of floor or ceiling effects), the app will provide a warning. In such cases, the use of that measure for studying individual differences should be reconsidered. A warning will also be issued if the error of the fit cannot be properly estimated for very small combinations of *L* and *N*.

## Conclusions

In summary, we validated our reformulation of the Spearman-Brown prophecy in a range of real-world cognitive tasks across many different cognitive domains, showing that the reliability of these tasks does eventually converge. We introduce a convergence coefficient *C*, which we conceptualize as a fundamental characteristic of a given task that can be used to evaluate its suitability for testing individual differences. We next explore the difference between split-halves and test-retest reliability on the tasks in our battery, and find that different tasks are differentially affected by measuring over different time points, with tasks with strong memory and attention components being most affected. This effect appears to be divided into a short-term, transient effect (such as mood, attention, arousal), which can be compensated for by averaging over 2–3 different testing days, and a more long-term effect (potentially reflecting real change in cognitive abilities over time). We further quantify this effect, so that the projected value of the test-retest reliability and its comparison to the split-halves reliability can be used to evaluate each task’s sensitivity to time. This can be particularly useful in longitudinal studies to investigate baseline shifts in cognitive abilities as opposed to transient changes. Finally, we provide the community with a simple online tool that computes the *C* coefficient, projects the reliability curve using only descriptive statistics of the experiment (mean, variance, number of participants and trials), and can be easily used to aid in designing new studies.

### Supplementary information

Peer Review File

Supplementary Information

Reporting summary

## Data Availability

The data presented in this article is openly available on Open Science Framework (OSF) under the following 10.17605/OSF.IO/CRE2B^116^.

## Code availability

All the analyses were performed using Python 3^117^ and the following packages: numpy^118^, pandas^119^, matplotlib^120^, seaborn^121^, lmfit^122^, scipy^123^, pingouin^124^. Final figures were created using the CanD^125^ package. The web app is designed using pyscript^126^, HTML, CSS and the same python packages as mentioned above. All the tasks were coded using lab.js^127^ (www.lab.js.org) and run on our servers. All code is accessible on GitHub (https://github.com/jankaWIS/reliability-paper) and Zenodo (https://zenodo.org/records/11564064)^128^. The online tool is available under the following link: https://jankawis.github.io/reliability-web-app/. The FAQs about the web app are part of the GitHub repository.
