# Supplementary material for: A measure of reliability convergence to select and optimize cognitive tasks for individual differences research
Source: Commun Psychol. 2024 Jul 4;2:64. doi: 10.1038/s44271-024-00114-4 (PMC11332135; doi:10.1038/s44271-024-00114-4)
Supplement: Supplementary file 2 — Supplementary Information [file 44271_2024_114_MOESM2_ESM.pdf]

A measure of reliability convergence to select and optimize cognitive tasks for individual differences research
Supplementary Information

Jan Kadlec[1], Catherine R. Walsh[2,3], Uri Sadé[4], Ariel Amir[4], Jesse Rissman[2,5], and Michal Ramot*[1]

[1]Department of Brain Sciences, Weizmann Institute of Science, Rehovot, Israel
[2]Department of Psychology, University of California, Los Angeles, CA, USA
[3]Present address: Section on Functional Imaging Methods, National Institute of Mental Health, Bethesda MD, USA
[4]Faculty of Physics, Weizmann Institute of Science, Rehovot, Israel
[5]Department of Psychiatry and Biobehavioral Sciences, University of California, Los Angeles, CA, USA

Contents

Supplementary Figures 3
- Supplementary Figure 1 . . . 3
- Supplementary Figure 2 . . . 4
- Supplementary Figure 3 . . . 5
- Supplementary Figure 4 . . . 6
- Supplementary Figure 5 . . . 7
- Supplementary Figure 6 . . . 8
- Supplementary Figure 7 . . . 9
- Supplementary Figure 8 . . . 10
- Supplementary Figure 9 . . . 10
- Supplementary Figure 10 . . . 11
- Supplementary Figure 11 . . . 12
- Supplementary Figure 12 . . . 13

Supplementary Note 14
- Instructions for use of the online tool . . . 14

Supplementary Methods 15
- Derivation of the closed formula . . . 15
  - Definitions and assumptions . . . 15
  - Calculation . . . 15
- Particular case – Tasks with binary outcomes . . . 18
  - Calculating C for the beta distribution . . . 18
- Computing Var(P) and $\mathbb{E}[P^2]$ from the participants' scores . . . 19

*Corresponding author: michal.ramot@weizmann.ac.il

Background and assumptions 19
Calculating Var(P) 19
Calculating $\mathbb{E}[P^2]$ 20
Calculating C 20
Derivation of the Attenuation-Correction Formula 21
Change in variance in constrained datasets 22
Statistical significance testing for CFMT curves 22
Comparing test-retest reliability with split-halves reliability 23
Alpha/beta for generating beta distributions 24

Supplementary Table – Abbreviations **25**

Supplementary References **26**

Supplementary Figures

Fit beta distributions to data

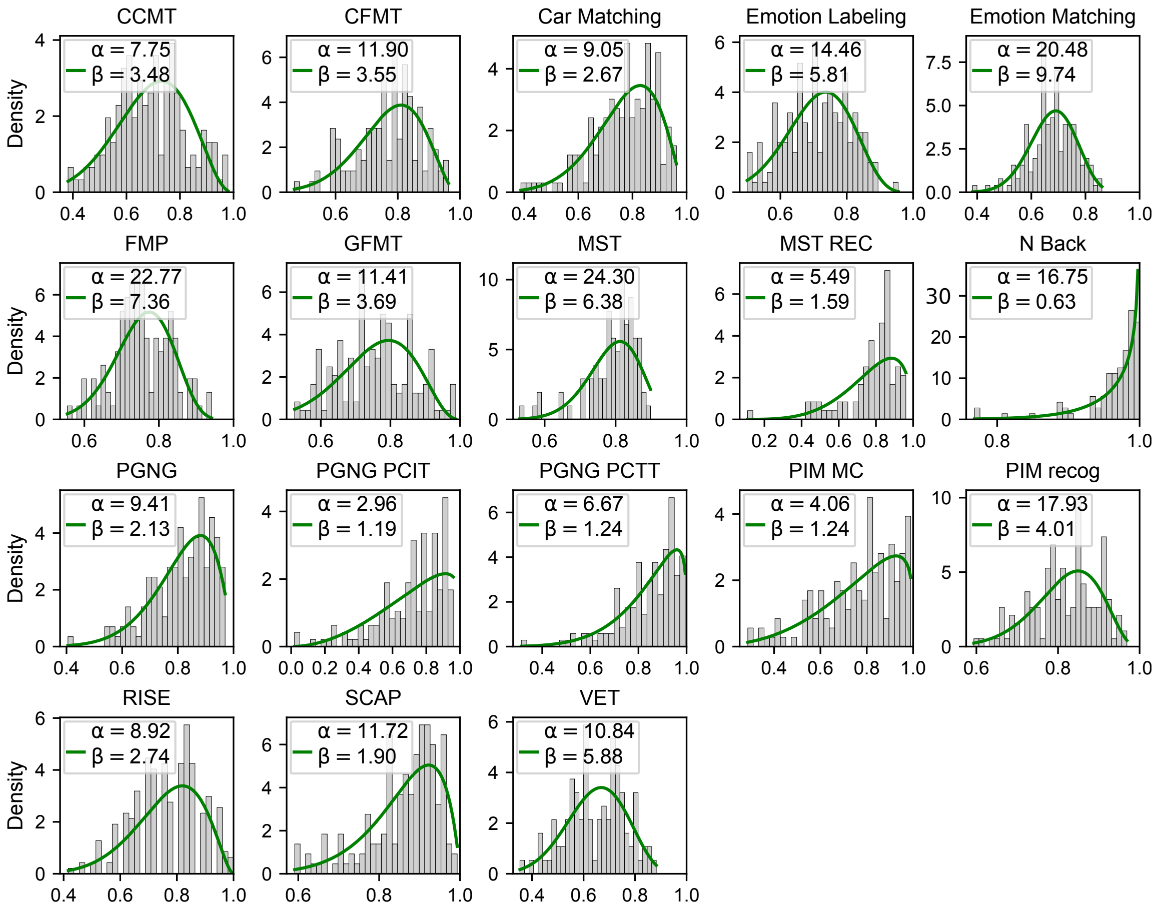

Supplementary Figure 1. Fit of beta distributions to all relevant measures (18). The barplot shows the distributions of scores, and the green line shows the best fit.

Alpha/beta for generating beta distributions

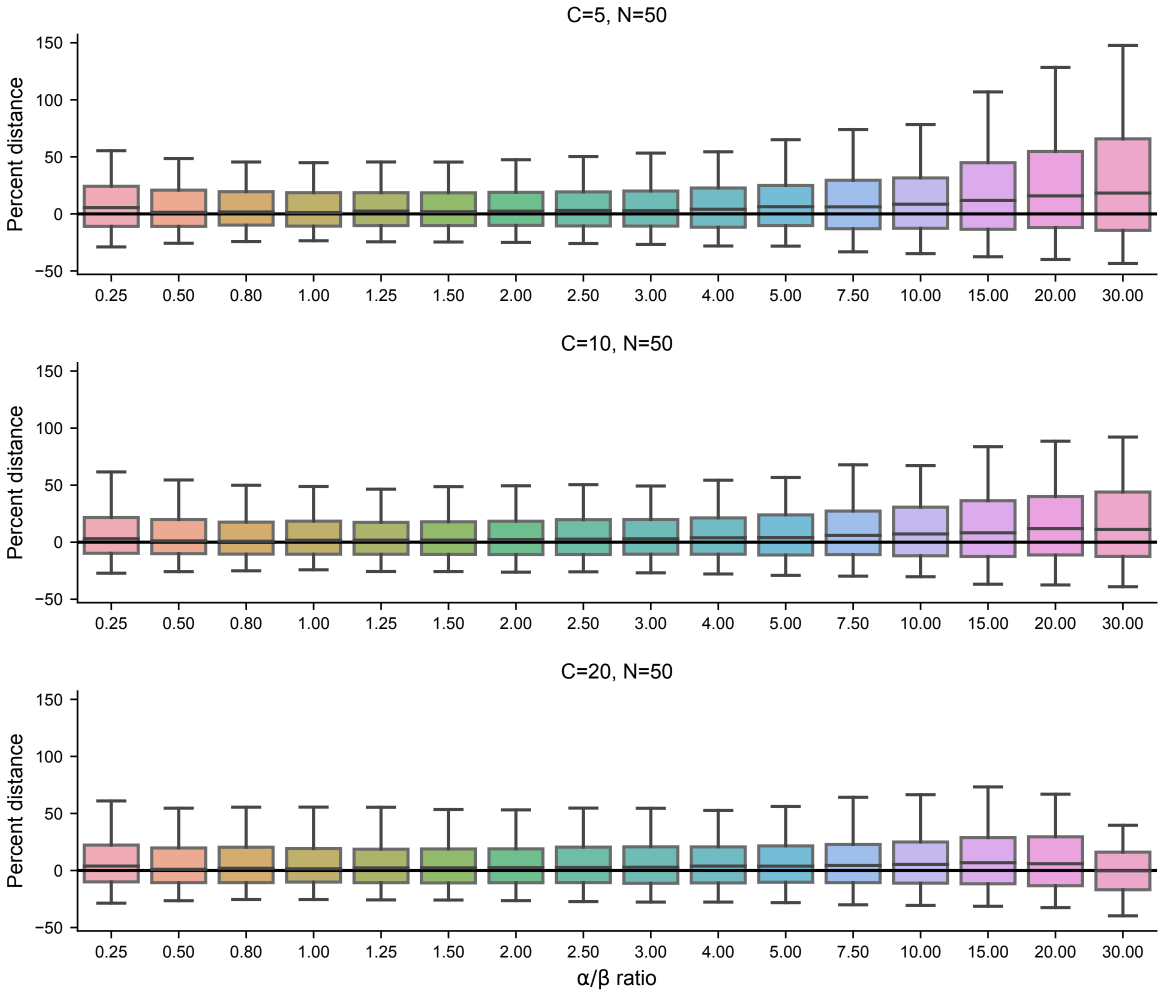

Supplementary Figure 2. Ratios of alpha to beta (α/β) for generating beta distributions have a small effect on the percent error when estimating C coefficient using the MV fit.

Dependence of median error in C estimation using MV fit on the value of C

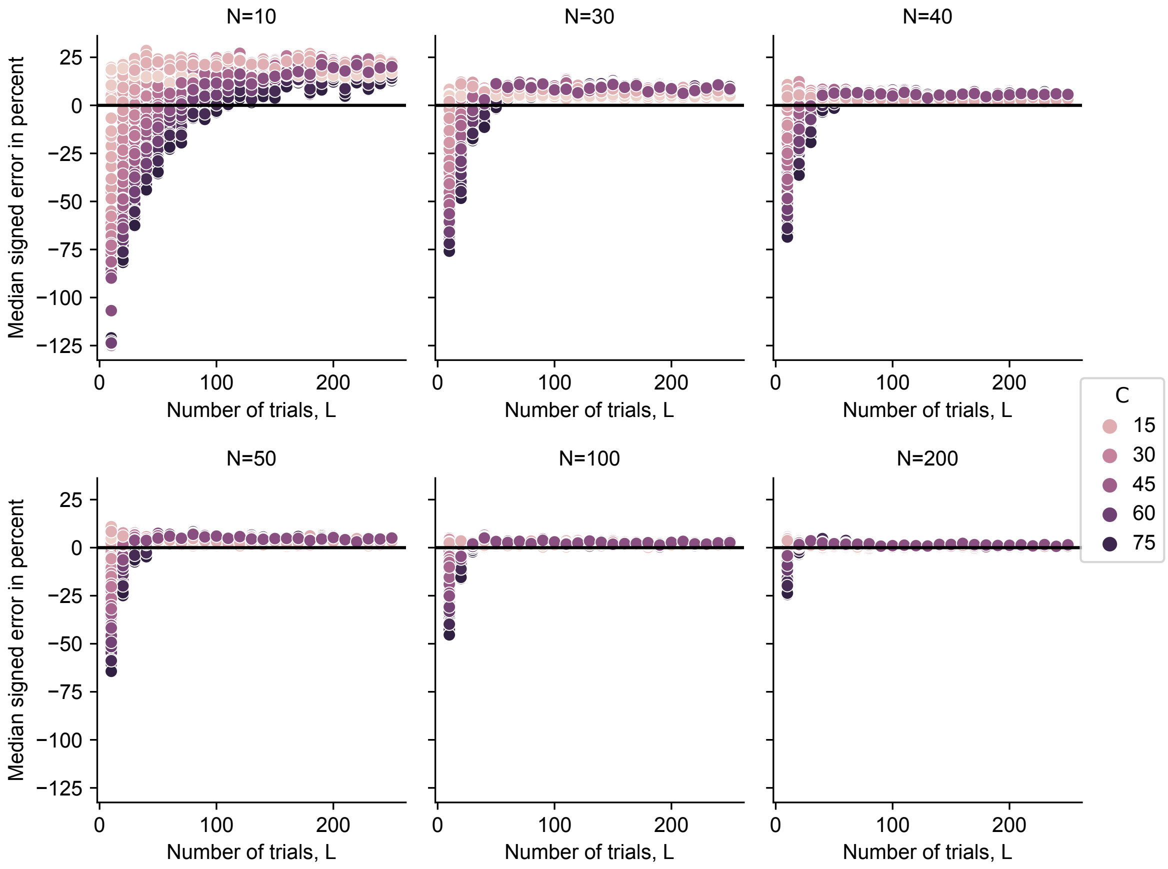

Supplementary Figure 3. Dependence of median error in C estimation using the MV fit on the value of C using synthetic data.

Curves for all tasks

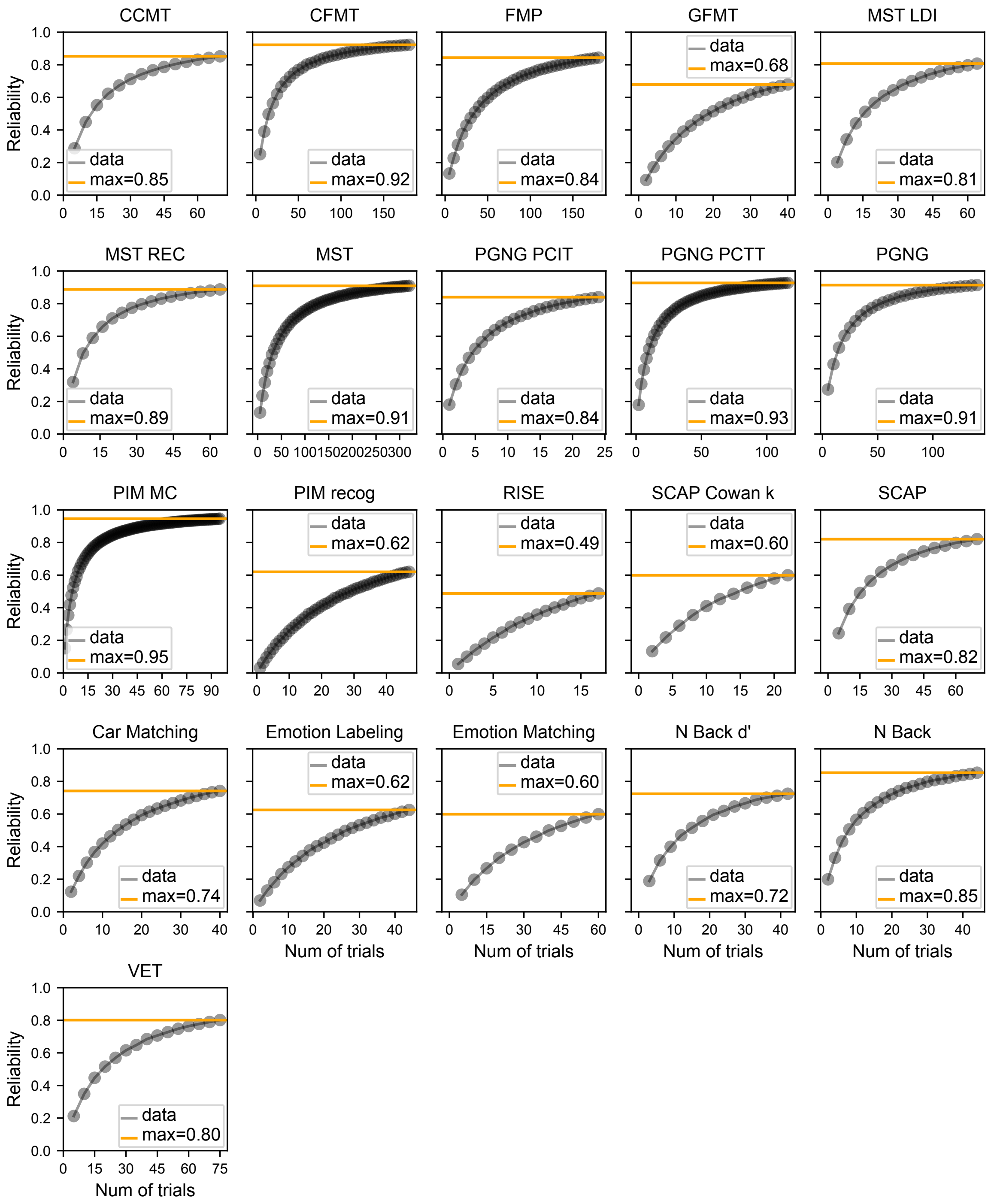

Supplementary Figure 4. Reliability curves for all behavioral measures (21) in our battery.

Correlation between all tasks

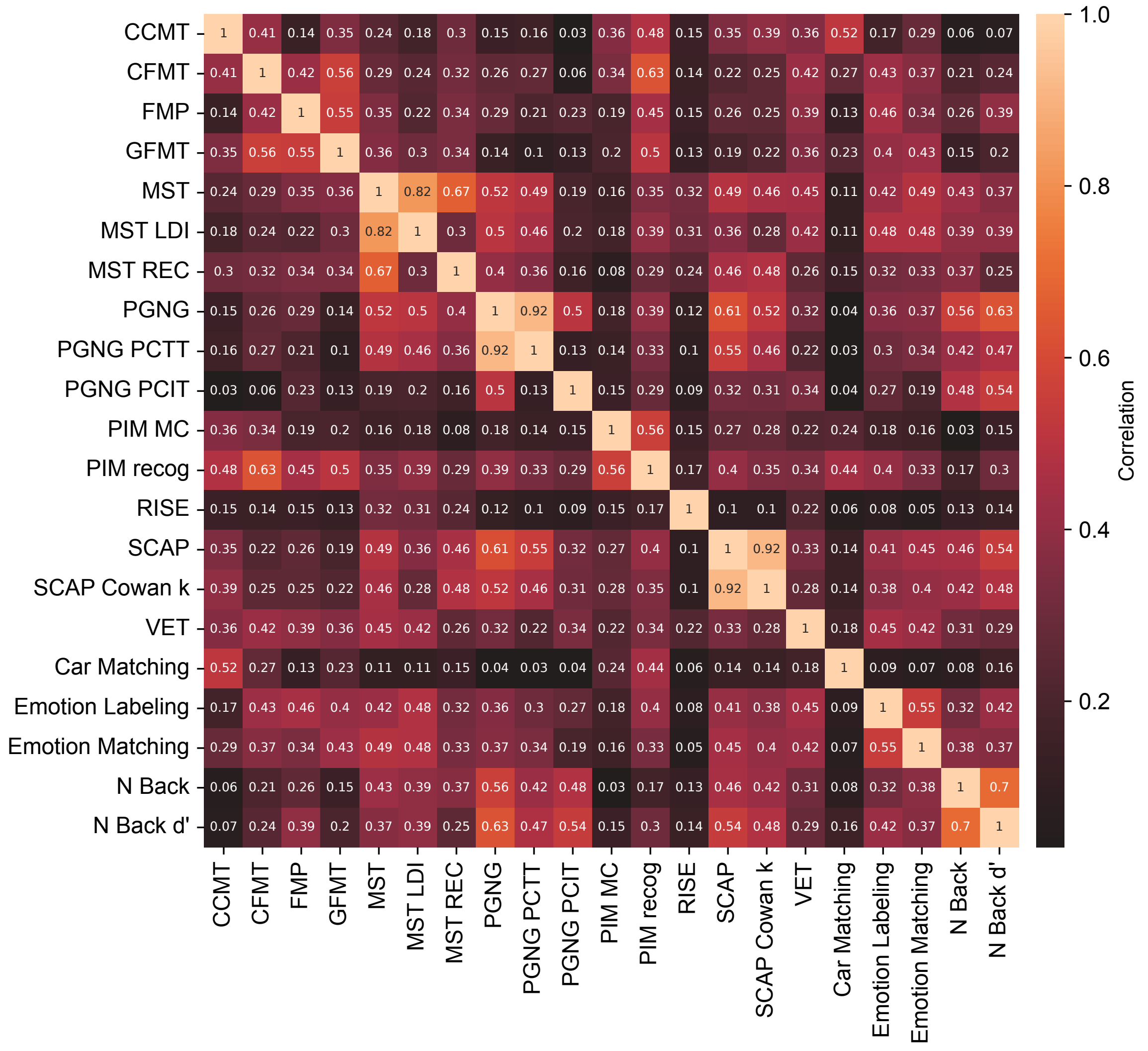

Supplementary Figure 5. Correlation between measures across all tasks (14) and measures (21).

Change in variance in constrained datasets

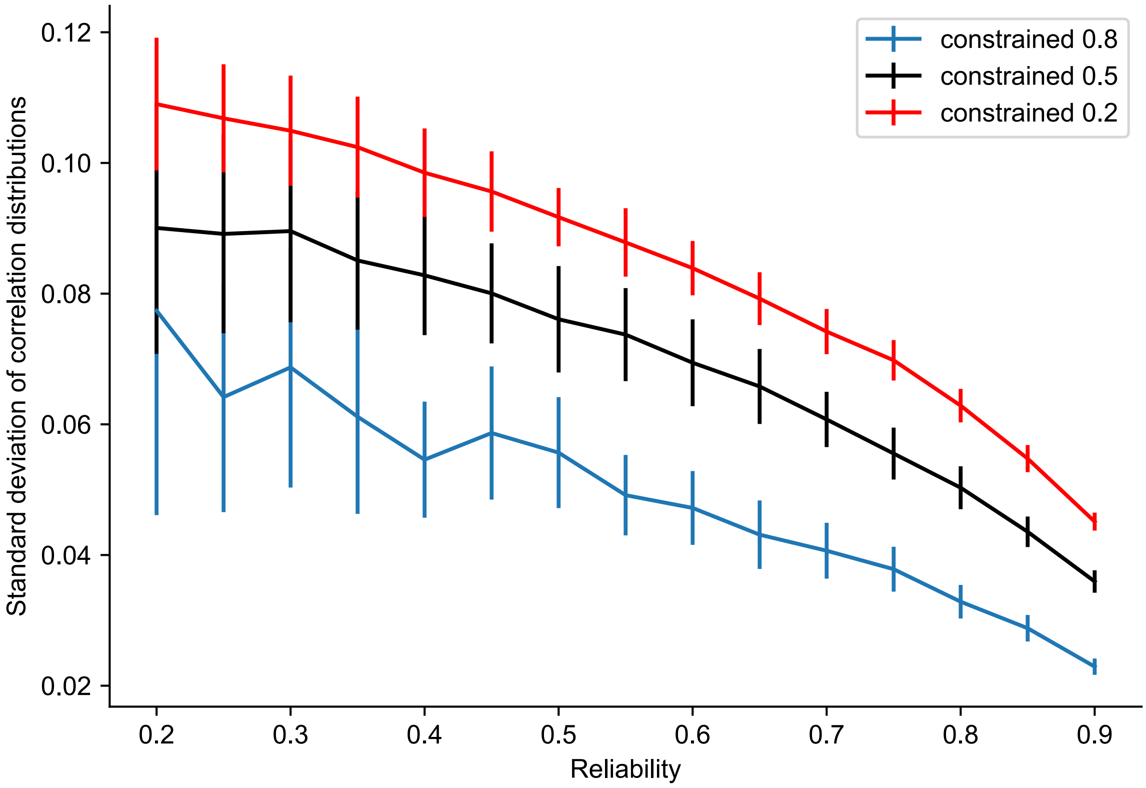

Supplementary Figure 6. Mean standard deviation of correlation distributions for reliability level across 100 simulations of 10 forms of synthetic datasets based on statistics from our real-world tasks for $N = 75$. Error bars depict the SD of the distribution. Colors show different levels of constraints of the dataset (i.e., what percentage of the dataset the L trials used to calculate reliability consisted of) when calculating reliability to ensure the reduction of standard deviation with increased reliability was not an artefact of decreased variance in the subsampling. Standard deviation decreases with increasing reliability (regardless of the size of the dataset that trials were sampled from), suggesting that the correlation between the tasks is truly better estimated and not the result of an artificial reduction in variance.

Comparison of fitting methods

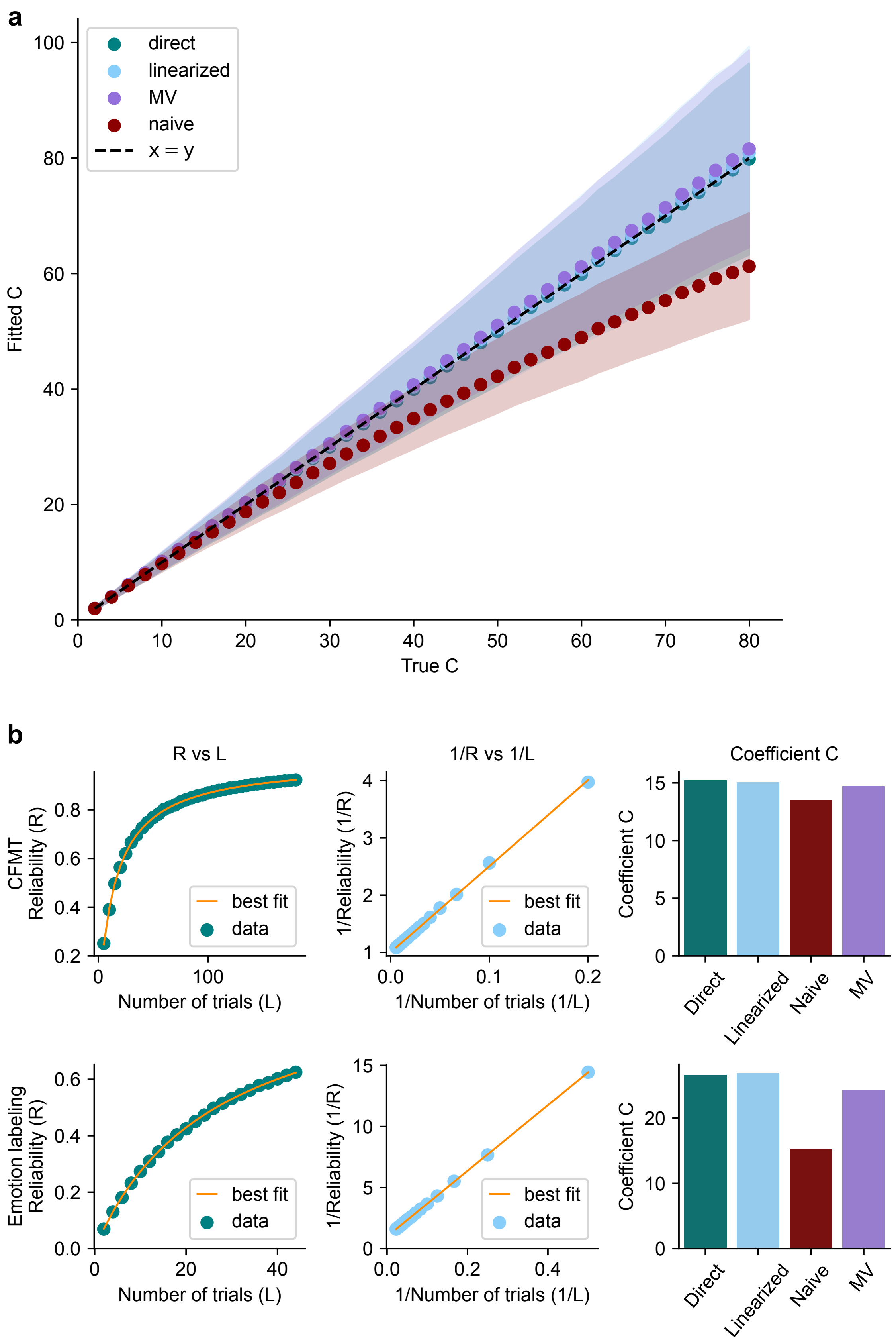

Supplementary Figure 7. **a.** True C versus fitted C using four different methods derived and discussed in the paper for $N = 100$ and $L = 250$. The naive method (red) used to fit the data using Eq. 2 consistently underestimates the C coefficient. **b.** Comparison of C

(continued) estimations using different methods for CFMT (top) and Emotion labeling (bottom) tasks. The first column shows the direct fit (Eq. 1), the middle column depicts linearized fit (Eq. 4), and the right column shows C values obtained for each of the four fits (Eq. 1-4).

Intercept values (Quality Check for linearized fit)

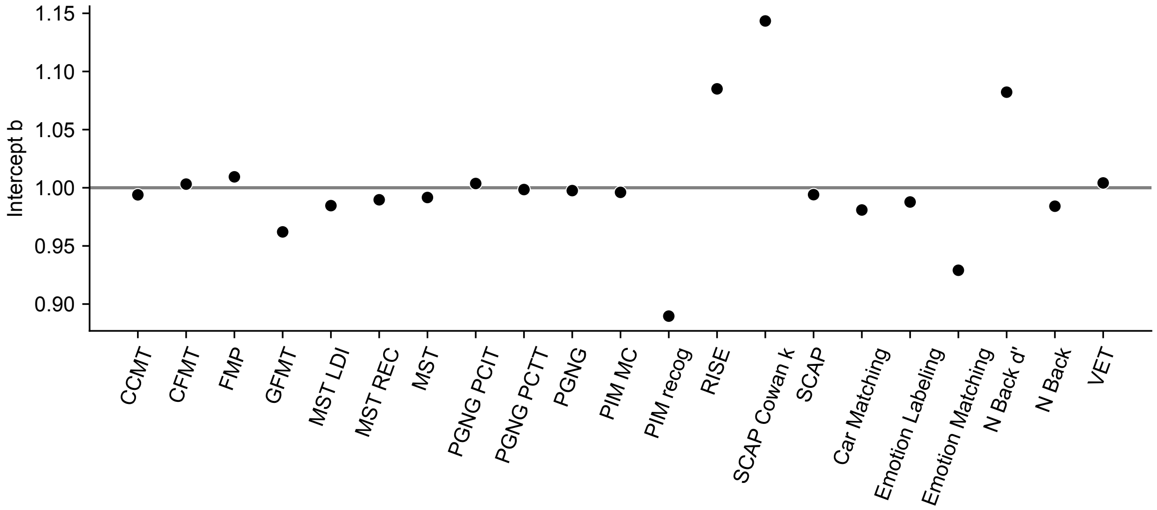

Supplementary Figure 8. Values of intercept for linearized fit across all the measures (21). According to Eq. 4, the intercept value should be equal to one (horizontal line). Deviations of the intercept value from one suggest a violation of the assumptions (no learning/fatigue).

Dependence of standard deviation of percent error in C estimation using the MV fit on the number of trials

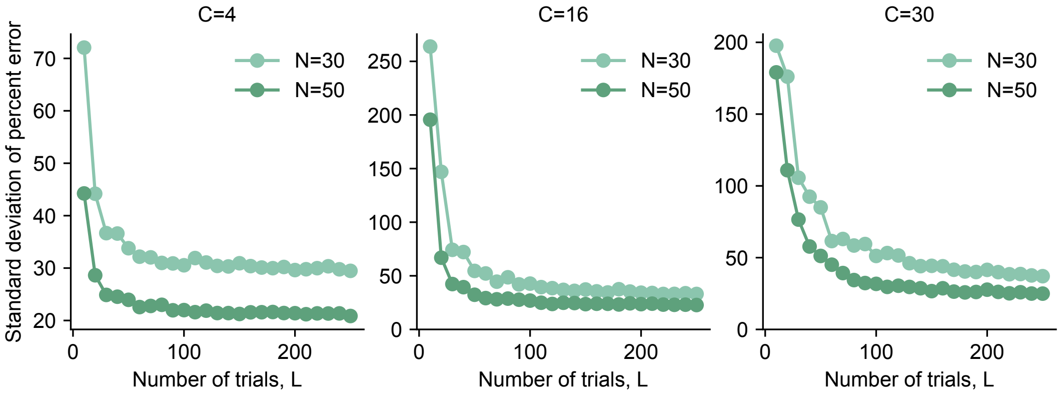

Supplementary Figure 9. Dependence of standard deviation of percent error in C estimation using the MV fit on the number of trials using synthetic data.

Reliability curves for test-retest and split-halves reliabilities across days on all tasks

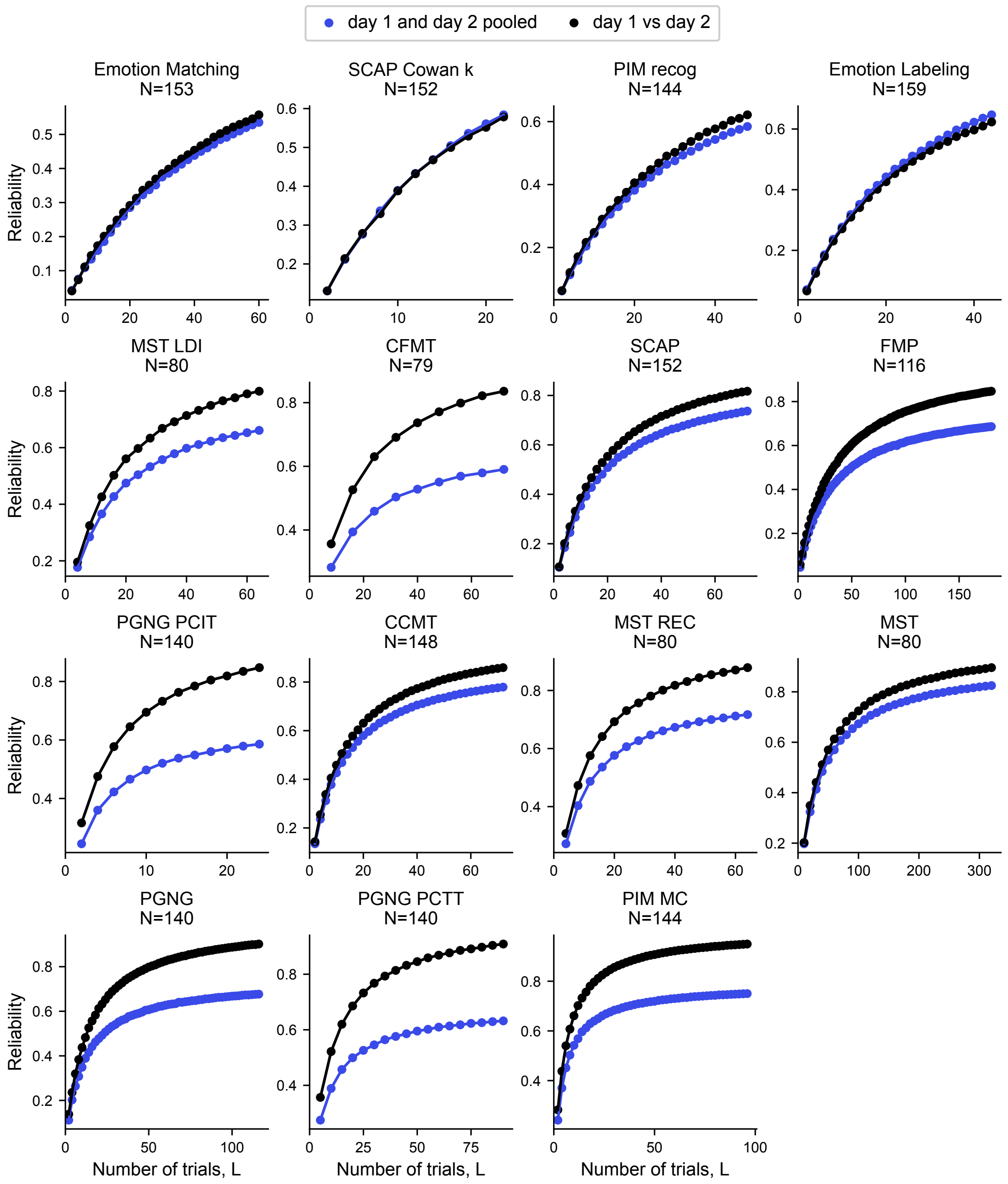

Supplementary Figure 10. The effect of time on reliability across all tasks and measures (15 in total), with sessions occurring at a median interval of 236 days. The black curve shows pooled data from both days, and the light blue curve shows reliability of day 1 vs day 2. Note the effect of sessions is not the same for all tasks.

Scatterplots for reliabilities across days for all tasks

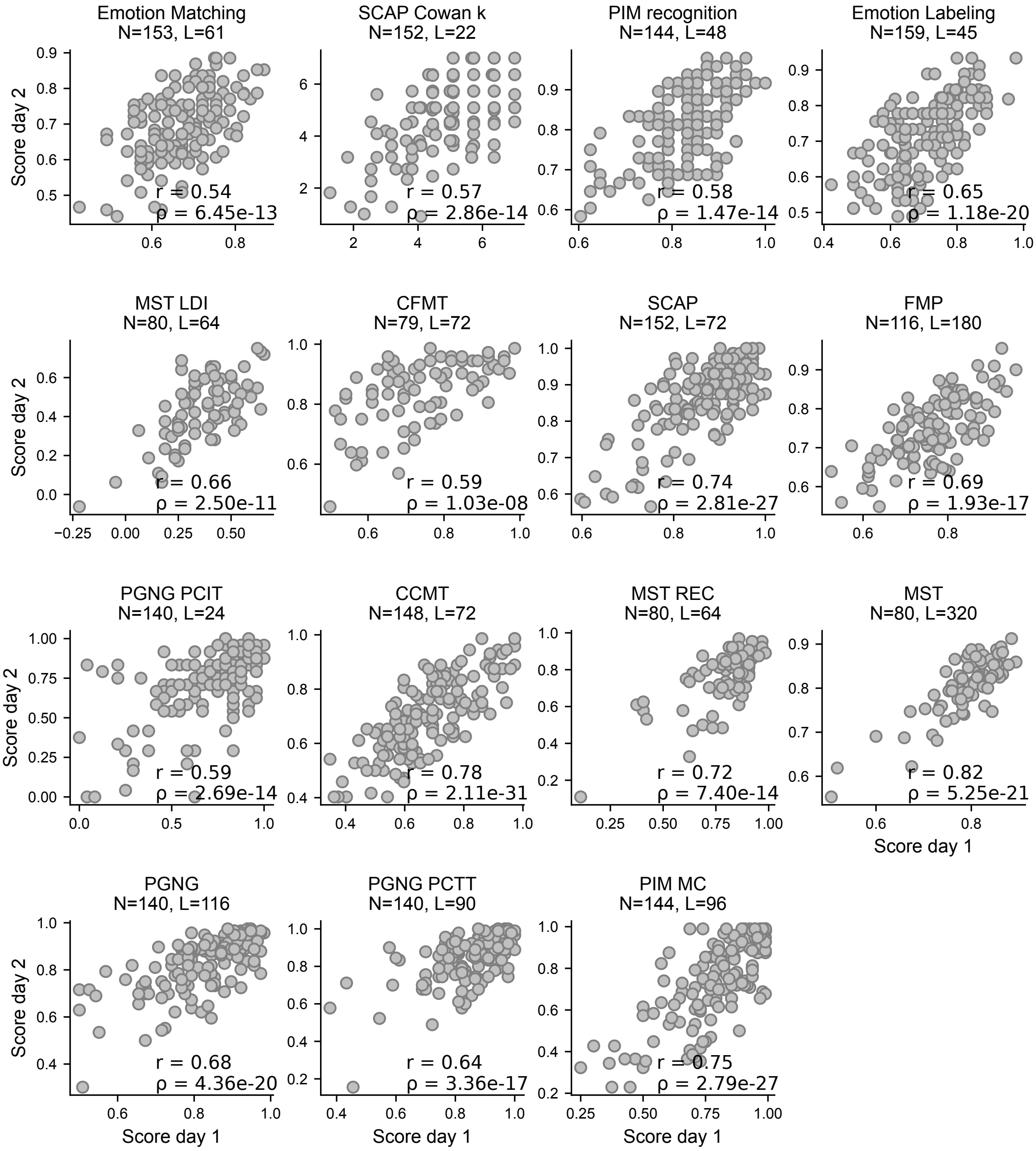

Supplementary Figure 11. Supplementary figure to Figure 7 from the main text and Supplementary Figure 10 showing the underlying scatterplots that give rise to the reliability curves. Shown is a random iteration from the 1000 iterations for the largest L in the task. Each dot is a participant; on the x-axis is their score on L trials of day 1, and on the y-axis is their score on L trials of day 2.

Web application for reliability estimates

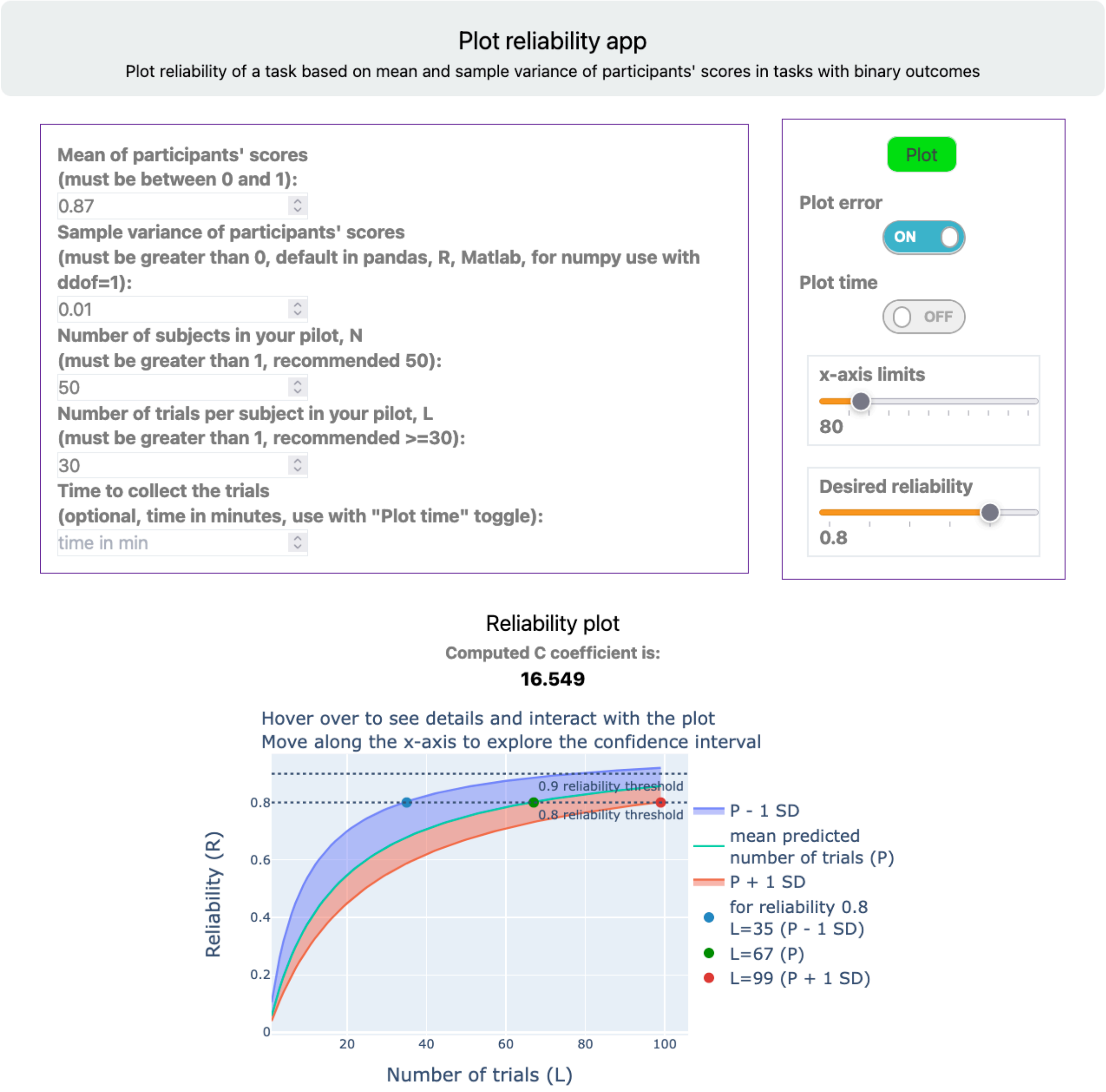

Supplementary Figure 12. Web application for reliability estimates.

Supplementary Note

Instructions for use of the online tool

To use the online web application (https://jankawis.github.io/reliability-web-app/), follow this protocol:

1. Run your pilot experiment with a small number of participants and trials per participant (we recommend $L \geq 30$, and at least $N = 30$, though preferably $N \sim 50$. Using a smaller number of trials or participants is possible, but will result in larger confidence intervals).

2. Calculate the mean score for each participant across trials.

3. Calculate the mean ($\mathbb{E}[Z]$) and sample variance (Var(Z)) of those scores across the group of scores. Sample variance is the default in many statistical tools (pandas, R, Matlab) except for numpy where `ddof=1` has to be explicitly used.

4. Enter the mean, variance, and the number of participants and trials per participant into the online tool and plot the corresponding reliability curve.

Toggle the reliability R to see how many trials L you need to reach your desired reliability level. You can also optionally provide the time it took to collect these L trials and plot reliability versus the time it would take to collect the necessary number of trials.

Supplementary Methods

Derivation of the closed formula

Definitions and assumptions

We assume that there are $N \gg 1$ participants performing a given task. A task consists of $2 \cdot L$ trials, and the result of a single task is a single score per participant. We assume that each participant i has some value representing their "true proficiency" (true score) at the task, p_i, and the outcome of a single trial, d_i^j, is a random variable (whose properties depend on p_i, and with j an arbitrary index, since the variables d_i^j are i.i.d. random variables). We assume there is no learning and no fatigue, i.e., samples are independent. In turn, the proficiency p_i is also assumed to be a random variable and is assumed to be drawn independently for each participant from the continuous probability distribution $f(P)$. Throughout, we will use P to denote the random variable associated with the proficiency distribution, e.g., $\mathbb{E}[P] = \int f(P)pdp$.

We define the *sum* of the outcomes of trials performed by participant i in, without loss of generality, the first L trials as the random variable x_i and in the last L by the random variable y_i. Since the outcomes of the trials for a participant are independent, $\mathbb{E}[x_i] = L \cdot \mathbb{E}[d_i^j]$ and $\text{Var}(x_i) = L \cdot \text{Var}(d_i^j)$.

We define two vectors of length N: $\vec{X}$, whose i-th element is x_i and $\vec{Y}$, whose i-th element is y_i. Our goal is to calculate the Pearson correlation between two vectors representing the average scores of participants, each across L trials. This is equivalent to calculating the Pearson correlation between the sum of the participants' trial outcomes instead of their average, so we will use sums for the sake of simpler calculations.

Finally, we define a random variable x_p, with the same properties as that of x_i but when fixing the proficiency to be a specific value p. Similarly, d_p will denote the performance in a single trial.

Calculation

The Pearson correlation coefficient for a sample is defined by:

$$R = \sum_{i=1}^{N} \frac{(x_i - \overline{x})(y_i - \overline{y})}{\sigma_x \sigma_y}, \tag{1}$$

where $\overline{x}$ and $\overline{y}$ denote the means of $\vec{X}$'s and $\vec{Y}$'s elements respectively, and σ their corresponding standard deviations.

For the next step, we group the participants by proficiency. The inner sum is over all the participants with a given proficiency p: $\{x_{p_j}\}$

$$R=\sum_{i=1}^{N}\frac{\left(x_i-\overline{x}\right)\left(y_i-\overline{y}\right)}{\sigma_x\sigma_y}= \tag{2}$$

$$=\sum_{p}\frac{\sum_j\left(x_{p_j}-\overline{x}\right)\left(y_{p_j}-\overline{y}\right)}{\sigma_x\sigma_y}= \tag{3}$$

$$=\sum_{p}\frac{\sum_j\left[\left(x_{p_j}-\mathbb{E}[x_p]\right)+\left(\mathbb{E}[x_p]-\overline{x}\right)\right]\left[\left(y_{p_j}-\mathbb{E}[x_p]\right)+\left(\mathbb{E}[x_p]-\overline{y}\right)\right]}{\sigma_x\sigma_y}. \tag{4}$$

Since we assumed large N, we will assume the sample standard deviations and means are close enough to the population standard deviations and expectations. That gives us $\overline{x}=\mathbb{E}[x_i]=\mathbb{E}[y_i]=\overline{y}$. Additionally, it is clear that $\mathbb{E}[x_p]=\mathbb{E}[y_p]$ and $\sigma_x=\sigma_y$. Using those facts and substituting $\sigma_x^2=\text{Var}(x)$, we can further simplify the equation into the following formula:

$$R=\sum_{p}\frac{\sum_j\left[\left(x_{p_j}-\mathbb{E}[x_p]\right)+\left(\mathbb{E}[x_p]-\overline{x}\right)\right]\left[\left(y_{p_j}-\mathbb{E}[x_p]\right)+\left(\mathbb{E}[x_p]-\overline{y}\right)\right]}{\sigma_x\sigma_y}= \tag{5}$$

$$=\frac{1}{\text{Var}(x)}\sum_p\sum_j\left(\left(x_{p_j}-\mathbb{E}[x_p]\right)\left(y_{p_j}-\mathbb{E}[x_p]\right)+\right. \tag{6}$$

$$\left.+\left[\left(x_{p_j}-\mathbb{E}[x_p]\right)+\left(y_{p_j}-\mathbb{E}[x_p]\right)\right]\left(\mathbb{E}[x_p]-\overline{x}\right)+\left(\mathbb{E}[x_p]-\overline{x}\right)^2\right). \tag{7}$$

Since $x_{p_j}-\mathbb{E}[x_p]$ and $y_{p_j}-\mathbb{E}[x_p]$ are fluctuations around their respective expected values, and $\vec{X}$ and $\vec{Y}$ are independent, the averages of their products vanish. Therefore, we obtain:

$$R=\sum_p\frac{\left(\mathbb{E}[x_p]-\overline{x}\right)^2}{\text{Var}(x)}. \tag{8}$$

Next, we calculate $\overline{x}$:

$$\overline{x}=\mathbb{E}[x_i]=\mathbb{E}[\mathbb{E}[x_i|p_i=p]], \tag{9}$$

with $\mathbb{E}[x_i|p_i=p]=\mathbb{E}[x_p]$ denoting conditioning on the proficiency of the participant being equal to p, and the outer expectation value is the average over all participants (accounting for the distribution of proficiencies). This leads to:

$$\overline{x}=\int F\left(p\right)\mathbb{E}[x_p]dp=L\cdot\int F\left(p\right)\mathbb{E}[d_p]dp. \tag{10}$$

Additionally:

$$\mathbb{E}[x_p^2]=\text{Var}(x_p)+\mathbb{E}[x_p]^2=L\cdot\text{Var}(d_p)+L^2\cdot\mathbb{E}[d_p]^2, \tag{11}$$

and:

$$\mathbb{E}[x^2] = \mathbb{E}[\mathbb{E}[x^2|P]] = \int f(P)\left(\mathbb{E}[x^2|P=p]\right)dp = \int f(P)\left(\mathbb{E}[x_p^2]\right)dp = \tag{12}$$

$$= \int f(P)\left(L \cdot \text{Var}(d_p) + L^2 \cdot \mathbb{E}[d_p]^2\right)dp. \tag{13}$$

Using that, we can calculate Var(x):

$$\text{Var}(x) = \mathbb{E}[x^2] - \mathbb{E}[x]^2 = \tag{14}$$

$$= \int f(P)\left(L \cdot Var(d_p) + L^2 \cdot \mathbb{E}[d_p]^2\right)dp - \left(L\int F(p)\,\mathbb{E}[d_p]dp\right)^2 = \tag{15}$$

$$= AL^2 + BL, \tag{16}$$

where we defined the two new coefficients (independent of L):

$$A = \int f(P)\mathbb{E}[d_p]^2 dp - \left(\int F(p)\,\mathbb{E}[d_p]dp\right)^2 = \text{Var}(\mathbb{E}[d_P]), \tag{17}$$

$$B = \int f(P)\text{Var}(d_p)dp. \tag{18}$$

In classical test theory, A corresponds to true-score variance, and B is the error variance for our single trials.

Returning to Eq. 8 and using what we derived, we can express reliability in the following way:

$$R = \sum_p \frac{(\mathbb{E}[x_p] - \overline{x})^2}{\text{Var}(x)} = \tag{19}$$

$$= \frac{1}{AL^2 + BL}\int f(P)\left(L \cdot \mathbb{E}[d_p] - L \cdot \int F(p)\,\mathbb{E}[d_p]dp\right)^2 dp = \tag{20}$$

$$= \frac{L}{AL+B}\text{Var}(\mathbb{E}[d_P]) = \tag{21}$$

$$= \frac{L}{AL+B}A. \tag{22}$$

That leads us to the final formula:

$$R = \frac{L}{AL+B}A = \frac{L}{L+\frac{B}{A}}. \tag{23}$$

We can denote by C the ratio B/A:

$$C = \frac{B}{A} = \frac{\int f(P)\text{Var}(d_p)dp}{\text{Var}(\mathbb{E}[d_P])}. \tag{24}$$

The relationship between L and R that we derived can be expressed as

$$R = \frac{L}{L+C}. \tag{25}$$

Note that this is equivalent to the famous Spearman-Brown formula.[1,2]

Particular case – Tasks with binary outcomes

If the tasks given to the participants have binary outcomes (e.g., 0/1, correct/incorrect, ...), we have $d_p \sim Bernoulli(p)$. Using that, we can derive an explicit formula for $R(L)$. Following from that, we know:

$$\mathbb{E}[d_p] = p, \tag{26}$$
$$\text{Var}(d_p) = p(1-p). \tag{27}$$

We can calculate C explicitly:

$$C = \frac{B}{A} = \frac{\int f(P)\text{Var}(d_p)dp}{\text{Var}(\mathbb{E}[d_P])} = \tag{28}$$
$$= \frac{\int f(P)p(1-p)dp}{\int f(P)p^2dp - \left(\int F\left(p\right)pdp\right)^2} = \tag{29}$$
$$= \frac{\mathbb{E}[P] - \mathbb{E}[P^2]}{\mathbb{E}[P^2] - \left(\mathbb{E}[P]\right)^2} = \frac{\mathbb{E}[P] - \mathbb{E}[P^2]}{\text{Var}(P)}. \tag{30}$$

We know that:

$$\text{Var}(P) = \mathbb{E}[P^2] - \mathbb{E}[P]^2, \tag{31}$$
$$\mathbb{E}[P^2] = \text{Var}(P) + \mathbb{E}[P]^2. \tag{32}$$

So we can simplify the expression into:

$$C = \frac{\mathbb{E}[P] - \mathbb{E}[P^2]}{\text{Var}(P)} = \frac{\mathbb{E}[P] - \text{Var}(P) - \mathbb{E}[P]^2}{\text{Var}(P)} = \frac{\mathbb{E}[P] - \mathbb{E}[P]^2}{\text{Var}(P)} - 1. \tag{33}$$

Calculating C for the beta distribution

For the beta distribution defined by two parameters, α and β, we can derive the value of C explicitly. The mean and the variance of beta distribution can be expressed using those two parameters in the following way:

$$\mathbb{E}[P] = \frac{\alpha}{\alpha+\beta}, \tag{34}$$
$$\text{Var}(P) = \frac{\alpha\beta}{(\alpha+\beta+1)(\alpha+\beta)^2}. \tag{35}$$

Substituting to Eq. 33, we get:

$$\begin{aligned}
C &= \frac{\frac{\alpha}{\alpha+\beta} - \left(\frac{\alpha}{\alpha+\beta}\right)^2}{\frac{\alpha\beta}{(\alpha+\beta+1)(\alpha+\beta)^2}} - 1, \\
&= \frac{\frac{\alpha(\alpha+\beta)}{(\alpha+\beta)^2} - \frac{\alpha^2}{(\alpha+\beta)^2}}{\frac{\alpha\beta}{(\alpha+\beta+1)(\alpha+\beta)^2}} - 1, \\
&= \frac{\frac{\alpha\beta}{(\alpha+\beta)^2}}{\frac{\alpha\beta}{(\alpha+\beta+1)(\alpha+\beta)}} - 1, \\
&= \frac{\alpha\beta}{(\alpha+\beta)^2} \cdot \frac{(\alpha+\beta+1)(\alpha+\beta)^2}{a\beta} - 1, \\
&= \alpha + \beta.
\end{aligned}$$

Computing Var(P) and $\mathbb{E}[P^2]$ from the participants' scores

Background and assumptions

The previous calculations derived the Pearson correlation coefficient, theoretically, for a population size $\to \infty$ (i.e., this is implicit when we take the ensemble average). When handling real data, we can only calculate the Pearson correlation coefficient for a finite population size. This correlation coefficient would approach the theoretical value as $N \to \infty$. Since we use a finite number of participants, we should expect some error between the theoretical value of the coefficient and the value calculated from a finite population, but no bias.

There is, however, another source of error. When trying to calculate C from real data, we never have access to the real participants' proficiencies. We can never know P, and we are thus introducing an error by having a limited number of trials per participant when estimating their proficiencies.

We have, nonetheless, the scores of the participants (x_i for the i-th participant) from which we can estimate the p_i of the i-th participant using $\frac{x_i+y_i}{2L}$. This naive approach, however, creates a bias when calculating $\mathbb{E}[P^2]$ and Var(P). As these are used in Eq. 33, we would expect to see a bias if we used this equation in this form (as shown in Supplementary Fig. 4). In this section, we derive a formula that does not have this bias and will allow us to estimate C from data accurately.

For simplicity, we assume a binary case, where a participant's score with proficiency p will be given by a random variable with distribution $x_p \sim B(2L, p)$ (binomial). We define a random variable $Z \sim \frac{B(2L,P)}{2L}$ that provides an estimate of the participant's proficiency.

Our goal in this part is to calculate how Var(Z) depends both on Var(P) and on L, enabling us to express C using the statistics of Z and thus overcoming the bias stemming from calculating the proficiencies naively when given a limited number of trials per participant.

Calculating Var(P)

The law of total variance dictates that:

$$\text{Var}(Z) = \mathbb{E}[\text{Var}(Z|P)] + \text{Var}(\mathbb{E}[Z|P]). \tag{36}$$

Using:

$$\mathbb{E}[Z|P=p] = \mathbb{E}\left[\frac{B(2L,p)}{2L}\right] = \frac{2Lp}{2L} = p, \tag{37}$$

we can calculate:

$$\text{Var}(\mathbb{E}[Z|P]) = \frac{\sum_{i=1}^{N}(p_i - \mu_P)^2}{N} = \text{Var}(P). \tag{38}$$

Next, we use:

$$\text{Var}(Z|P=p) = \text{Var}\left(\frac{B(2L,p)}{2L}\right) = \frac{2Lp(1-p)}{(2L)^2} = \frac{p(1-p)}{2L}, \tag{39}$$

to calculate:

$$\mathbb{E}[\text{Var}(Z|P)] = \frac{\sum_{i=1}^{N}\frac{p_i(1-p_i))}{2L}}{N} = \frac{1}{2L}\cdot\frac{\sum_{i=1}^{N}p_i(1-p_i)}{N}. \tag{40}$$

Putting it together, we find:

$$\text{Var}(Z) = \mathbb{E}[\text{Var}(Z|P)] + \text{Var}(\mathbb{E}[Z|P]) = \text{Var}(P) + \frac{1}{2L} \cdot \frac{\sum_{i=1}^{N} p_i(1-p_i)}{N}, \tag{41}$$

$$\text{Var}(P) = \text{Var}(Z) - \frac{1}{2L} \cdot \frac{\sum_{i=1}^{N} p_i(1-p_i)}{N} = \text{Var}(Z) - \frac{\mathbb{E}[P] - \mathbb{E}[P^2]}{2L}. \tag{42}$$

Calculating $\mathbb{E}[P^2]$

We observe that:

$$\mathbb{E}[P^2] = \frac{\sum_{i=1}^{N} p_i^2}{N}, \tag{43}$$

and:

$$Z^2 = \frac{B(2L, P)^2}{(2L)^2}. \tag{44}$$

From the expected value of the square of a binomial variable, we can now see that:

$$\mathbb{E}[Z^2|P = p] = \frac{1}{(2L)^2} \left((2L)^2 p^2 + 2Lp\,(1-p) \right). \tag{45}$$

Using the law of total expectation, we can now calculate the following:

$$\mathbb{E}[Z^2] = \mathbb{E}[\mathbb{E}[Z^2|P]] = \frac{\sum_{i=1}^{N} \left(p_i^2 + \frac{p_i(1-p_i)}{2L} \right)}{N} = \tag{46}$$

$$= \mathbb{E}[P^2] + \frac{\mathbb{E}[P]}{2L} - \frac{\mathbb{E}[P^2]}{2L}. \tag{47}$$

Similarly,

$$\mathbb{E}[P] = \mathbb{E}[Z]. \tag{48}$$

Finally, we obtain:

$$\mathbb{E}[P^2] = \frac{2L\mathbb{E}[Z^2] - \mathbb{E}[Z]}{2L - 1}. \tag{49}$$

Calculating C

We know that:

$$C = \frac{\mathbb{E}[P] - \mathbb{E}[P]^2}{\text{Var}(P)} - 1. \tag{50}$$

Using Eqs. 42 and 49, we can derive:

$$C = \frac{\mathbb{E}[Z] - \mathbb{E}[Z]^2}{\mathrm{Var}(Z) - \frac{\mathbb{E}[P]-\mathbb{E}[P^2]}{2L}} - 1 = \tag{51}$$

$$= \frac{\mathbb{E}[Z] - \mathbb{E}[Z]^2}{\mathrm{Var}(Z) - \frac{\mathbb{E}[Z]-\frac{2L\mathbb{E}[Z^2]-\mathbb{E}[Z]}{2L-1}}{2L}} - 1 = \tag{52}$$

$$= \frac{\mathbb{E}[Z] - \mathbb{E}[Z]^2}{\mathrm{Var}(Z) - \frac{\mathbb{E}[Z]-\mathbb{E}[Z^2]}{2L-1}} - 1 = \tag{53}$$

$$= \frac{(2L-1)\left(\mathbb{E}[Z] - \mathbb{E}[Z]^2\right) - (2L-1)\mathrm{Var}(Z) + \mathbb{E}[Z] - \mathbb{E}[Z^2]}{(2L-1)\mathrm{Var}(Z) + \mathbb{E}[Z] - \mathbb{E}[Z^2]} = \tag{54}$$

$$= \frac{(2L-1)\left(\mathbb{E}[Z] - \mathbb{E}[Z]^2\right) - (2L-1)\left(\mathbb{E}[Z^2] - \mathbb{E}[Z]^2\right) + \mathbb{E}[Z] - \mathbb{E}[Z^2]}{(2L-1)\left(\mathbb{E}[Z^2] - \mathbb{E}[Z]^2\right) + \mathbb{E}[Z] - \mathbb{E}[Z^2]} = \tag{55}$$

$$= \frac{2L\mathbb{E}[Z] - 2L\mathbb{E}[Z]^2}{2L\mathbb{E}[Z^2] - 2L\mathbb{E}[2]^2 - \mathbb{E}[Z] + \mathbb{E}[Z]^2} = \tag{56}$$

$$= \frac{\mathbb{E}[Z] - \mathbb{E}[Z^2]}{\mathrm{Var}(Z) - \frac{\mathbb{E}[Z]-\mathbb{E}[Z]^2}{2L}}. \tag{57}$$

Note that this is an explicit formula for the coefficient C using *observed* data alone. Eq. 57 is reminiscent of an equation previously described by Kuder and Richardson (KR-21[3–5]).

Derivation of the Attenuation-Correction Formula

In this section, we derive the Attenuation-Correction formula, first formulated by Spearman[1,6] and further developed by others,[7–9] to represent the correlation between two quantities represented by vectors of their measurements, $\vec{X}$ and $\vec{Y}$, using the internal reliabilities of those measurements. We start by defining $\vec{X}$ and $\vec{Y}$ as:

$$\vec{X'} = \vec{X} + \vec{e_1}, \tag{58}$$

$$\vec{Y'} = \vec{Y} + \vec{e_2}, \tag{59}$$

where $\vec{X'}, \vec{Y'}$ are vectors representing the measured scores, $\vec{X}, \vec{Y}$ are vectors representing the real, underlying proficiencies, and $\vec{e_1}, \vec{e_2}$ are the errors from the measurement, respectively. We assume that $\vec{e_1}$, $\vec{e_2}$ are independent, and also that $\vec{X}$, $\vec{e_i}$ are independent (same for $\vec{Y}$). We note that the Pearson correlation between two variables can be written as:

$$R_{XY} = \frac{\mathrm{Cov}(X,Y)}{\sigma_X \sigma_Y}. \tag{60}$$

If we denote X_1' and X_2' as the split-halves of X', we can use Eq. 60 to see that the internal reliabilities of the measurement vectors are:

$$R_{X'X'} = \frac{\mathrm{Cov}(X_1', X_2')}{\sigma_{X'}\sigma_{X'}} = \frac{\mathrm{Cov}(X + e_1, X + e_1)}{\mathrm{Var}(X')} = \frac{\mathrm{Var}(X)}{\mathrm{Var}(X')}. \tag{61}$$

Analogous formula gives $R_{Y'Y'}$. Now we can calculate the following:

$$R_{X'Y'} = \frac{\text{Cov}(X', Y')}{\sigma_{X'}\sigma_{Y'}} = \frac{\text{Cov}(X + e_1, Y + e_2)}{\sigma_{X'}\sigma_{Y'}} = \frac{\text{Cov}(X, Y)}{\sigma_{X'}\sigma_{Y'}} = \tag{62}$$

$$= \frac{R_{XY}\sigma_X\sigma_Y}{\sigma_{X'}\sigma_{Y'}} = R_{XY}\sqrt{\frac{\text{Var}(X)}{\text{Var}(X')}}\sqrt{\frac{\text{Var}(Y)}{\text{Var}(Y')}} = \tag{63}$$

$$= R_{XY}\sqrt{R_{X'X'}R_{Y'Y'}}. \tag{64}$$

Change in variance in constrained datasets

To calculate the standard deviation of the correlations between the tasks shown in Supplementary Figure 6, we first created two synthetic datasets that mirror the observed distributions of tasks from our real-world dataset. Using the mean accuracy from 75 participants who had two forms of data from each of the pairs of tasks, we generated ten synthetic forms of data per participant that preserved both the reliability of each task as well as their observed correlations. Next, we calculated how many trials (L) would be necessary for each task to achieve a given level of reliability, as was done in previous analyses. Using the ten forms of synthetic data, we created three constrained datasets, where the required number of trials L would comprise 80%, 50% or 20% of the total synthetic dataset. For example, if a task required 9 trials to achieve a reliability of 0.3, we would first randomly select $9/0.8 = 11$ trials for our most constrained sample dataset, $9/0.5 = 18$ trials for our moderately constrained sample dataset, and $9/0.2 = 45$ trials for our minimally constrained sample dataset. For each level of reliability, the required L trials were then randomly sampled 1000 times from within these three constrained datasets. The resulting correlations (and the mean and standard deviation across the iterations) between the two pairs of tasks were therefore calculated based only on subsampling from the relevant constrained dataset. This entire simulation process was repeated 100 times, each time randomly selecting the constrained dataset from the full set of ten synthetic task forms. This yielded 100 values of standard deviations for each reliability level and each constrained dataset, and Supplementary Figure 6 plots the mean and standard deviation of these values.

Statistical significance testing for CFMT curves

We employed permutation tests to statistically verify significance of the difference between reliability curves on the same day, separate days and months apart in Figure 7a. Since we only have data from 42 participants who completed two forms of the CFMT on the same day, compared with 77 and 79 who completed the two forms either days or months apart, we downsampled the other groups to match $N = 42$. We subsampled 100 times to create a distribution of potential results (as in Fig. 7b) for this constrained number of participants ($N = 42$). We first tested that the reliability of all three test-retest measures was significantly different from the pooled split-halves reliability, as above. To test whether the three curves are different from each other, another approach was needed, as these are different participants. For each subsample of participants, for each group (same day, days apart, and months apart), we first calculated test-retest reliability by randomly selecting half the trials in

each form ($L = 36$) and calculating the correlation between them. Using the attenuation correction formula, we then calculated the expected test-retest reliability that this would converge to for this particular sampling of trials, as described above. This procedure was repeated 1000 times, for 1000 random samplings of trials, giving us a distribution of 1000 values of expected test-retest reliability values for each group. For the months-apart and days-apart groups, we averaged over the 100 subsamples of participants. We then compared the three distributions and tested for significance. One-way ANOVA showed a significant effect of time ($F = 1071.9$, $p < 0.001$, $df = 2$). The effect size, as measured by the partial eta-squared, is substantial ($\eta p^2 = 0.417$). Follow-up post-hoc Tukey HSD revealed significant differences between all pairs of groups ($p < 0.001$) and the following statistics: same day vs months apart ($T = 29.01$, unbiased Cohen's d $= 1.30$, CI $= [0.0904, 0.1063]$), separate days vs months apart ($T = 45.76$, unbiased Cohen's d $= 2.05$, CI $= [0.1472, 0.1631]$), separate days vs same day ($T = 16.74$, unbiased Cohen's d $= 0.75$, CI $= [0.0488, 0.0647]$).

Comparing test-retest reliability with split-halves reliability

In this comparison, we included only tasks where the score difference between the first and second visits was not significant. We applied a more stringent significance threshold of $p < 0.01$ to exclude tasks and we excluded two tasks based on this criteria: GFMT (two-tailed t-test, $t_{300} = 3.12$, $p = 0.00199$, Cohen's d $= 0.36$, CI$=$ $[0.02, 0.07]$) and Car matching (two-tailed t-test, $t_{326} = -3.16$, $p = 0.0017$, Cohen's d $= 0.35$, CI$=$ $[-0.07, -0.02]$). Supplem. Table 1. summarizes statistics for all the tasks.

To determine test-retest reliability, i.e., the value the test-retest reliability curve would converge to as L goes to infinity, we used the attenuation correction formula in the following form:

$$R_{XY} = \frac{R_{X'Y'}}{\sqrt{R_{X'X'} \cdot R_{Y'Y'}}} \tag{65}$$

where for any L, $R_{X'Y'}$ is the observed correlation/reliability between variables X and Y (day 1 vs day 2), and $R_{X'X'}$, and $R_{Y'Y'}$, are the observed split-halves correlation/reliability of variable X (day 1 vs day 1), or Y (day 2 vs day 2) respectively. In Figure 7c, we compute this value for tasks that have two sessions months apart (a median of 236 days apart) and for the largest L possible for the given task – as close as possible to half the total number of trials in one form of the task as this is the maximum L that we can calculate single-day reliability for. Under the null hypothesis, if there is no effect of time, we expect this correlation to converge to 1 (black line) and the pooled-data split-halves reliability to be the same as test-retest reliability (blue and black curve overlapping in Supplementary Fig. 10).

To calculate whether the test-retest reliability was significantly different from split-halves reliability, we conducted a permutation test to estimate the range of reliabilities that could be expected from constraining the data into two arbitrary "pseudo days" and comparing them through a test-retest type analysis. We performed 1000 permutations, first pooling all the data across the two testing days and then randomly splitting it into two "days". We then performed the same test-retest analysis – computing reliability curves for pseudo day 1 vs pseudo day 2 for each permutation and using the attenuation correction formula to obtain the predicted correlation at convergence for infinite L. For each task in Figure 7c, (**) denotes the actual test-retest value on the real split of days being outside the 1st-99th percentile of the random distribution

Task	T	Degrees of freedom	p-val	CI95%	Cohen's d
Car Matching	-3.1641	326	0.0017	[-0.07, -0.02]	0.3494
CCMT	-1.2028	294	0.2300	[-0.05, 0.01]	0.1398
Emotion Labeling	-0.9212	316	0.3576	[-0.03, 0.01]	0.1033
Emotion Matching	-2.3099	304	0.0216	[-0.04, -0.00]	0.2641
FMP	1.6890	230	0.0926	[-0.00, 0.04]	0.2218
GFMT	3.1188	300	0.0020	[0.02, 0.07]	0.3589
MST	-1.3162	158	0.1900	[-0.04, 0.01]	0.2081
MST LDI	-2.4331	158	0.0161	[-0.11, -0.01]	0.3847
MST REC	1.1650	158	0.2458	[-0.02, 0.07]	0.1842
PGNG	-0.1954	278	0.8452	[-0.03, 0.02]	0.0234
PGNG PCTT	0.1075	278	0.9144	[-0.03, 0.03]	0.0129
PGNG PCIT	-0.6610	278	0.5092	[-0.07, 0.04]	0.0790
PIM MC	2.4827	286	0.0136	[0.01, 0.10]	0.2926
PIM recog	1.9667	286	0.0502	[-0.00, 0.04]	0.2318
SCAP	0.2561	302	0.7981	[-0.02, 0.02]	0.0294
SCAP Cowan's k	0.2861	302	0.7750	[-0.25, 0.33]	0.0328

Supplementary Table 1. Two-sided t-test results for differences between the first and the second visit.

($p < 0.01$). The p-values are determined as the number of values in the random distribution that were higher/lower than the measured test-retest reliability and are the following: PIM recog $172/1000$, Emotion Labeling $230/1000$, Emotion Matching $402/1000$, SCAP Cowan's k $388/1000$. All the other tasks were entirely outside of the random distribution with $0/1000$ ($p < 0.001$).

Alpha/beta for generating beta distributions

To assess the impact of the ratio of the two parameters, α and β, that define the beta distribution in our simulations, we selected three values of the C coefficient ($C = 5, 10, 20$), and for each C, we created 15 different combinations of α and β with the following α/β ratios: $0.25, 0.5, 0.8, 1, 1.25, 1.5, 2, 2.5, 3, 4, 5, 7.5, 10, 15, 20, 30$. Each pair of α and β, i.e., each ratio, generated a beta distribution for $N = 50$ participants, each with $L = 250$ trials. The remaining parts of the simulations were similar to the *Error estimation* of the different fits but using only the MV fit. We conducted 1000 simulations for each combination of C and the α/β ratio. The ground truth for the C coefficient was established for every combination of C and α/β separately by generating a large distribution with $N = 10^7$ and using the MV fit to get the C corresponding to this distribution. We computed the difference between the fitted C and this true C. We then divided this distance by the true C to get error in percent and plot it as boxplots in Supplementary Figure 2.

Supplementary Table – Abbreviations

Abbreviation	Meaning
CCMT	Cambridge Car Memory Task
CFMT	Cambridge Face Memory Task
FMP	Face Memory Perception task
GFMT	Glasgow Face Matching Task
MST	Mnemonic Similarity Task
MST LDI	Mnemonic Similarity Task – Lure Discrimination Index
MST REC	Mnemonic Similarity Task – Recognition accuracy score
MV fit	Mean/Variance fit, based on Eq. 3
PGNG	Parametric Go-No Go
PCTT	Percent Correct To Target
PCIT	Percent Correct to Inhibitory Trials
PIM	Personal Identity Memory
PIM MC	Personal Identity Memory – Multiple Choice test
PIM recog	Personal Identity Memory – Recognition test
RISE	Relational and Item-Specific Encoding
SCAP	Spatial Working Memory Capacity
VET	Vanderbilt Expertise Test

Supplementary Table 2. Abbreviations used in the study. See Methods for an explanation of the different measures.

Supplementary References

1. Spearman, C. Correlation Calculated from Faulty Data. en. *British Journal of Psychology, 1904-1920* **3,** 271–295 (1910).
2. Brown, W. Some Experimental Results in the Correlation of Mental Abilities1. en. *British Journal of Psychology, 1904-1920* **3,** 296–322 (1910).
3. Kuder, G. F. & Richardson, M. W. The theory of the estimation of test reliability. en. *Psychometrika* **2,** 151–160 (Sept. 1937).
4. Allen, M. J. & Yen, W. M. *Introduction to Measurement Theory* en (Waveland Press, Dec. 2001).
5. Thissen, D. & Wainer, H. *Test scoring* (Lawrence Erlbaum Associates Publishers, Mahwah, NJ, US, 2001).
6. Spearman, C. The Proof and Measurement of Association between Two Things. *The American Journal of Psychology* **15,** 72–101 (1904).
7. Johnson, H. G. Test reliability and correction for attenuation. en. *Psychometrika* **15,** 115–119 (June 1950).
8. Trafimow, D. The attenuation of correlation coefficients: a statistical literacy issue. en. *Teaching Statistics* **38,** 25–28 (2016).
9. Metsämuuronen, J. Attenuation-Corrected Estimators of Reliability. *Applied Psychological Measurement* **46,** 720–737 (Nov. 2022).
