# Supplementary material for: A measure of reliability convergence to select and optimize cognitive tasks for individual differences research
Source: Commun Psychol. 2024 Jul 4;2:64. doi: 10.1038/s44271-024-00114-4 (PMC11332135; doi:10.1038/s44271-024-00114-4)
Supplement: Supplementary file 3 — Reporting summary [file 44271_2024_114_MOESM3_ESM.pdf]

nature portfolio

Corresponding author(s): Michal Ramot

Last updated by author(s): Apr 14, 2024

Reporting Summary

Nature Portfolio wishes to improve the reproducibility of the work that we publish. This form provides structure for consistency and transparency in reporting. For further information on Nature Portfolio policies, see our Editorial Policies and the Editorial Policy Checklist.

Statistics

For all statistical analyses, confirm that the following items are present in the figure legend, table legend, main text, or Methods section.

n/a	Confirmed	
☐	☒	The exact sample size (*n*) for each experimental group/condition, given as a discrete number and unit of measurement
☒	☐	A statement on whether measurements were taken from distinct samples or whether the same sample was measured repeatedly
☐	☒	The statistical test(s) used AND whether they are one- or two-sided<br>*Only common tests should be described solely by name; describe more complex techniques in the Methods section.*
☒	☐	A description of all covariates tested
☐	☒	A description of any assumptions or corrections, such as tests of normality and adjustment for multiple comparisons
☐	☒	A full description of the statistical parameters including central tendency (e.g. means) or other basic estimates (e.g. regression coefficient) AND variation (e.g. standard deviation) or associated estimates of uncertainty (e.g. confidence intervals)
☐	☒	For null hypothesis testing, the test statistic (e.g. *F*, *t*, *r*) with confidence intervals, effect sizes, degrees of freedom and *P* value noted<br>*Give P values as exact values whenever suitable.*
☒	☐	For Bayesian analysis, information on the choice of priors and Markov chain Monte Carlo settings
☒	☐	For hierarchical and complex designs, identification of the appropriate level for tests and full reporting of outcomes
☐	☒	Estimates of effect sizes (e.g. Cohen's *d*, Pearson's *r*), indicating how they were calculated

Our web collection on statistics for biologists contains articles on many of the points above.

Software and code

Policy information about availability of computer code

Data collection	All the tasks were coded using lab.js (www.lab.js.org) and run on our servers.
Data analysis	All the analyses were performed using Python 3 and the following packages:<br>numpy, pandas, matplotlib, seaborn, lmfit, scipy, pingouin. Final figures were created using the CanD package. The web app is designed using pyscript, HTML, CSS and the same python packages as mentioned above.<br>All the tasks were coded using lab.js (www.lab.js.org) and run on our servers.

For manuscripts utilizing custom algorithms or software that are central to the research but not yet described in published literature, software must be made available to editors and reviewers. We strongly encourage code deposition in a community repository (e.g. GitHub). See the Nature Portfolio guidelines for submitting code & software for further information.

Data

Policy information about availability of data

All manuscripts must include a data availability statement. This statement should provide the following information, where applicable:

- Accession codes, unique identifiers, or web links for publicly available datasets
- A description of any restrictions on data availability
- For clinical datasets or third party data, please ensure that the statement adheres to our policy

Code and data will be made accessible on GitHub and OSF at the time of publication. Authors provide a link to reviewers to see all the code.

Human research participants

Policy information about studies involving human research participants and Sex and Gender in Research.

Reporting on sex and gender	We do not consider it in our analysis. Our results should be applicable regardless of sex/gender. Sex/gender were self-reported on Prolific.
Population characteristics	Out of them, a total number of N=257 (131 females, 120 males, 6 not stated, mean age: 29.8 ± 7.7) finished all tasks of the first experimental day and were included in our analyses. All demographics information are part of the data and will be available upon publishing. An additional dataset of N=206 (76 females, 129 males, mean age: 29.8 ± 8.3) participants were included during the review process..
Recruitment	All data were collected online and participants were recruited using the online platform Prolific (www.prolific.co). Participants received monetary compensation.
Ethics oversight	Weizmann and UCLA institutional review boards.

Note that full information on the approval of the study protocol must also be provided in the manuscript.

Field-specific reporting

Please select the one below that is the best fit for your research. If you are not sure, read the appropriate sections before making your selection.

☐ Life sciences ☒ Behavioural & social sciences ☐ Ecological, evolutionary & environmental sciences

For a reference copy of the document with all sections, see nature.com/documents/nr-reporting-summary-flat.pdf

Behavioural & social sciences study design

All studies must disclose on these points even when the disclosure is negative.

Study description	This study consists of both quantitative and qualitative measurements and simulations. We used a large battery of behavioral tasks to determine the number of trials needed to reliably measure individual differences in task performance and extended the Spearman-Brown prophecy to calculate this number of trials from even a small pilot sample. We present the C coefficient that allows direct comparison of tasks' suitability for individual differences. Additionally, we developed a user-friendly web-app to make this calculation more accessible.
Research sample	All data were collected online and participants were recruited using the online platform Prolific (www.prolific.co). 298 participants started the first day of the experiment. Out of them, a total number of N=257 (131 females, 120 males, 6 not stated, mean age: 29.8 ± 7.7) finished all tasks of the first experimental day and were included in our analyses. Of those, 244 completed the full 3-day battery. All demographics information are part of the data and will be available upon publishing. An additional dataset of N=206 (76 females, 129 males, mean age: 29.8 ± 8.3) participants were included during the review process.
Sampling strategy	We used a random sampling approach. The data used in this manuscript were collected with the goal of having at least 100 useable datasets (which we defined as 100 subjects missing less than 15% of all tasks). We chose this number because this dataset is also being used for a separate project where the main analysis is an exploratory factor analysis, which has a minimum recommended N of 100 participants. Because we administered our tasks online, we expected that not all participants will complete all days and all tasks, therefore we started with N~300. After the first round of analyses, we decided to collect more data per participant to verify predictions for large number of trials, L, given by our theory. We recruited participants on Prolific with the following eligibility criteria: age range 18-65, fluent in English, no ongoing mental health/illness/condition, approval rate >80, and chose the Standard sample method.
Data collection	All data were collected online (due to data collection occurring during the COVID19 pandemic) and participants were recruited using the online platform Prolific (www.prolific.co). 298 participants started the first day of the experiment. Out of them, a total number of N=257 (131 females, 120 males, 6 not stated, mean age: 29.8 ± 7.7) finished all tasks of the first experimental day and were included

in our analyses. Of those, 244 completed the full 3-day battery. An additional dataset of N=206 (76 females, 129 males, mean age: 29.8 ± 8.3) participants were included during the review process.

Timing

The first collection was done in June, 2021. The subsequent collection was done in February and March 2022. The additional dataset included during the review process was collected between December 2021 and February 2022.

Data exclusions

All the relevant details are in Methods. We chose to exclude participants on a per-task basis, based on a combination of their accuracy, reaction time (RT) and individual trial responses. We assessed the following criteria and excluded participants from a task if two or more of the following criteria were met:

Average RT was 2 standard deviations (SD) faster than the group mean.
Standard deviation of RT was less than 2 SD below the standard deviation of the group.
Average sequence length of a single response was 2 SD greater than the group mean sequence length.

If a participant's accuracy was greater than 0.5 SD below the mean, they were included, regardless of their RT and individual trial responses. The only exception to this was for the MST, where if the standard deviation of the RT was less than 2 SD below the SD of the group, they were excluded regardless of performance, as this pattern of responses suggested that the task was being performed by a script/bot rather than a human.

After excluding participants based on accuracy, RT and individual trial responses, we additionally excluded participants based on the following criteria, which would indicate that they were not paying attention during the tasks:

CFMT: Four or more incorrect trials in Stage 1 (i.e., Stage 1 score less than 83%). For participants with three incorrect trials in Stage 1, data were excluded if performance on the other stages indicated a lack of attention rather than a valid measure of poor performance.
FMP: Accuracy below chance (50%) in face-matching trials.
PGNG: Accuracy less than 3 SD below the mean for the two target identification stages. If accuracy was less than 2 SD below the mean, performance on the rest of the task was evaluated to determine whether low accuracy was because of a genuine lower performance or lack of attention.
N-Back: Accuracy less than 3 SD below the mean for the 1-back blocks. If accuracy was less than 2 SD below the mean, performance on the rest of the task was evaluated to determine whether low accuracy was because of a genuine lower performance or lack of attention.
VET tasks: Incorrect or missing responses on 2 out of 3 catch trials.

When comparing test-retest reliability and split-halves reliability on pooled data, we implemented another exclusion criteria. To ensure that the effect is not driven by outliers, we removed all participants from the analyses whose difference in score between the two sessions was more than 2 SD from the mean differences. This allowed us to remove a small number of participants (3-12 participants, no more than 8 % of the total sample size for each task) that were exceptionally good or bad on one day and not on the other – a pattern suggestive of inconsistent data quality. In the case of the longitudinal FMP dataset, we performed this exclusion on score differences between every comparison and removed the union of all excluded participants, in total 23 out of 206 participants were removed.

Non-participation

298 participants started the first day of the experiment. Out of them, a total number of N=257 (131 females, 120 males, 6 not stated, mean age: 29.8 ± 7.7) finished all tasks of the first experimental day and were included in our analyses. Of those, 244 completed the full 3-day battery. At this stage we introduced two additional tasks to the battery – the Vanderbilt Expertise Task (birds, leaves and planes subscales) and a visual N-Back task with fractal stimuli, which included 0-back, 1-back and 2-back conditions. A total of N=183 returned and did some portion of the tasks (see Table 1 for counts of participants per task). 89 participants successfully completed all tasks with at least one repetition. Of these, 41 participants successfully completed all the repetitions.

In the newly added dataset that contained 6 sessions, N=271 started the longitudinal experiment out of which 216 successfully finished all sessions.

Randomization

The order of tasks was pseudo-random with some tasks being always the first/last in a given session while the rest was randomised across participants. Participants were not split into any groups thus no randomisation occurred.

Reporting for specific materials, systems and methods

We require information from authors about some types of materials, experimental systems and methods used in many studies. Here, indicate whether each material, system or method listed is relevant to your study. If you are not sure if a list item applies to your research, read the appropriate section before selecting a response.

Materials & experimental systems

n/a	Involved in the study	
☒	☐	Antibodies
☒	☐	Eukaryotic cell lines
☒	☐	Palaeontology and archaeology
☒	☐	Animals and other organisms
☒	☐	Clinical data
☒	☐	Dual use research of concern

Methods

n/a	Involved in the study	
☒	☐	ChIP-seq
☒	☐	Flow cytometry
☒	☐	MRI-based neuroimaging
