# Supplementary material for: A measure of reliability convergence to select and optimize cognitive tasks for individual differences research
Source: Commun Psychol. 2024 Jul 4;2:64. doi: 10.1038/s44271-024-00114-4 (PMC11332135; doi:10.1038/s44271-024-00114-4)
Supplement: Supplementary file 1 — Peer Review File [file 44271_2024_114_MOESM1_ESM.pdf]

nature portfolio

Peer Review File

A measure of reliability convergence to select and optimize cognitive tasks for individual differences research

Open Access This file is licensed under a Creative Commons Attribution 4.0 International License, which permits use, sharing, adaptation, distribution and reproduction in any medium or format, as long as you give appropriate credit to the original author(s) and the source, provide a link to the Creative Commons license, and indicate if changes were made. In the cases where the authors are anonymous, such as is the case for the reports of anonymous peer reviewers, author attribution should be to 'Anonymous Referee' followed by a clear attribution to the source work. The images or other third party material in this file are included in the article's Creative Commons license, unless indicated otherwise in a credit line to the material. If material is not included in the article's Creative Commons license and your intended use is not permitted by statutory regulation or exceeds the permitted use, you will need to obtain permission directly from the copyright holder. To view a copy of this license, visit http://creativecommons.org/licenses/by/4.0/.

Web links to the author's journal account have been redacted from the decision letters as indicated to maintain confidentiality

25th Sep 23

Dear Michal,

Thank you for your patience during the peer-review process. I very much regret the delay in returning to you with a decision. As I mentioned previously, one referee unfortunately had to withdraw from the evaluation process, which led to significant delay.

Your manuscript titled "Putting cognitive tasks on trial: A measure of reliability convergence" has now been seen by 2 reviewers, and I include their comments at the end of this message. They find your work of interest, but raised some important points.

As you will see below, the concerns raised by the referees are mainly presentational, emphasizing that while useful, the app will not resolve all the issues that the reader might expect. They also highlight that some revisions are needed to broaden the accessibility of the work. Editorially, and partially informed by the reviewers' feedback, we find that the work is not suitable for our Research Article format and would instead need to be published as a Resource (https://www.nature.com/commspsychol/submit/content-types#resource)

We are interested in the possibility of publishing your work as a Resource in Communications Psychology; if you are amenable to that change in format, we would like to consider your revised manuscript before we make a final decision on publication. We therefore invite you to revise and resubmit your manuscript, along with a point-by-point response to the reviewers. Please highlight all changes in the manuscript text file.

Resources in Communications Psychology include the following sections: Abstract; Introduction; Methods and Data; Validation; and Discussion (including a Limitations section). The empirical work included in your manuscript would need to be presented in the "Validation" section, which is obligatory in this case. It is understood that the Methods and Data section will focus on the Methods, rather than Data.

We are committed to providing a fair and constructive peer-review process. Please don't hesitate to contact us if you wish to discuss the revision in more detail.

Please use the following link to submit your revised manuscript and point-by-point response to the referees’ comments (which should be in a separate document to any cover letter):

[link redacted]

** This url links to your confidential home page and associated information about manuscripts you may have submitted or be reviewing for us. If you wish to forward this email to co-authors, please delete the link to your homepage first **

We hope to receive your revised paper within 8-12 weeks; please let us know if you aren't able to submit it within this time so that we can discuss how best to proceed.

We would appreciate it if you could keep us informed about an estimated timescale for resubmission, to facilitate our planning.

Please do not hesitate to contact me if you have any questions or would like to discuss these revisions further. We look forward to seeing the revised manuscript and thank you for the opportunity to review your work.

Best wishes,

Marike

Marike Schiffer, PhD

Chief Editor

Communications Psychology

EDITORIAL POLICIES AND FORMATTING

We ask that you ensure your manuscript complies with our editorial policies. Please ensure that the following formatting requirements are met, and any checklist relevant to your research is completed and uploaded as a Related Manuscript file type with the revised article.

Editorial Policy: Policy requirements (Download the link to your computer as a PDF.)

* TRANSPARENT PEER REVIEW: Communications Psychology uses a transparent peer review system. This means that we publish the editorial decision letters including Reviewers' comments to the authors and the author rebuttal letters online as a supplementary peer review file. However, on author request, confidential information and data can be removed from the published reviewer reports and rebuttal letters prior to publication. If your manuscript has been previously reviewed at another journal, those Reviewers' comments would not form part of the published peer review file.

* CODE AVAILABILITY: All Communications Psychology manuscripts must include a section titled "Code Availability" at the end of the methods section. In the event of publication, we require that the custom analysis code supporting your conclusions is made available in a publicly accessible repository; at publication, we ask you to choose a repository that provides a DOI for the code; the link to the repository and the DOI will need to be included in the Code Availability statement. Publication as Supplementary Information will not suffice. We ask you to prepare code at this stage, to avoid delays later on in the process.

* DATA AVAILABILITY:

All Communications Psychology manuscripts must include a section titled "Data Availability" at the end of the Methods section or main text (if no Methods). More information on this policy, is available at http://www.nature.com/authors/policies/data/data-availability-statements-data-citations.pdf.

At a minimum the Data availability statement must explain how the data can be obtained and whether there are any restrictions on data sharing. Communications Psychology strongly endorses open sharing of data. If you do make your data openly available, please include in the statement:

- Unique identifiers (such as DOIs and hyperlinks for datasets in public repositories)

- Accession codes where appropriate

- If applicable, a statement regarding data available with restrictions

- If a dataset has a Digital Object Identifier (DOI) as its unique identifier, we strongly encourage including this in the Reference list and citing the dataset in the Data Availability Statement.

We recommend submitting the data to discipline-specific, community-recognized repositories, where possible and a list of recommended repositories is provided at http://www.nature.com/sdata/policies/repositories.

If a community resource is unavailable, data can be submitted to generalist repositories such as figshare or Dryad Digital Repository. Please provide a unique identifier for the data (for example a DOI or a permanent URL) in the data availability statement, if possible. If the repository does not provide identifiers, we encourage authors to supply the search terms that will return the data. For data that have been obtained from publicly available sources, please provide a URL and the specific data product name in the data availability statement. Data with a DOI should be further cited in the methods reference section.

Please refer to our data policies at http://www.nature.com/authors/policies/availability.html.

REVIEWERS' EXPERTISE:

Reviewer #1: statistics, cognitive psychology

Reviewer #2: statistics, cognitive psychology

REVIEWERS' COMMENTS:

Reviewer #1 (Remarks to the Author):

This paper derives a formulation of reliability in terms of the mean and between-person variance of binary data that allows neuroscience researchers to input these summary statistics from pilot data into a web app to get a reliability curve. The resulting reliability curves can be used to adjudicate whether a particular measure is suited for individual differences research, and if it is, how many trials are required to attain acceptable reliability. The novelty of this method is that researchers are not required to compute reliability (e.g., coefficient alpha) from their pilot data, instead it is inferred from the mean and variance statistics.

I fully agree that cognitive scientists need to pay more attention to reliability of their measures (and measurement more generally) if they are interested in pursuing individual differences research. I also think the app is neat, and I hope researchers find it helpful. Technically there’s nothing really new here – I’m not sure I agree with the authors that this approach is “much easier” than

calculating alpha, which is readily accessible in any statistical software package – but if this paper brings some attention to the reliability phenomena that are documented in it, it will be a valuable contribution to the literature.

My primary concern with this paper is that its audience is not clear. My own area of study is psychometrics, not neuroscience, and I found this paper extremely difficult to follow. The notation used throughout is very different than the standard notation used in psychometrics and psychology, making the whole paper seem far more complicated and impenetrable than it really is. The math is quite simple, but I'm not sure that anyone in neuroscience who reads this will be able to follow.

The paper could be made more readable by making connections between the technical parts and the substantive interpretation. For example, the technical development in this paper is to state reliability in terms of a convergence coefficient "C", but nowhere in the paper is it made clear how to interpret this coefficient. What are its units? Are there thresholds for when C is so large that a measure shouldn't be used? It is helpful that Cs are given for a range of tasks, but I'm still not sure what C = 25 means.

Simulation results in Fig 4. are shown for 250 trials and 100 participants. But the app recommends 50 participants and >20 trials. What are these recommendations built on? Based on Figure 4, it looks like even with N = 100 and 250 trials, there is substantial variability around the estimated C coefficient, which makes sense since reliability is known to be subject to high sampling variability when reliability is low and sample sizes are small. Is there any evidence that the numbers recommended in the app are sufficient for estimation? Figure 5 shows that median error is negligible, but researchers will be subject to error on a single trial, not median error. If I'm reading Fig 5 correctly, then with N = 50 and L = 30, we're looking at a SD of 60 (80? it's very hard to differentiate these shades!) percent error, which seems substantial.

"we therefore define reliability (R) as…" what follows is not a definition but a way of estimating reliability.

On page 4 the term "samples" is used to refer to number of trials. This was incredibly confusing – it's typical to reserve "sample" and "sample size" and "subsample" to refer to participants, and "trials" for trials.

It would be useful to include some discussion of missing data. In some designs (e.g., infant data), it is common for participants to do as many trials as possible before getting fatigued, resulting in an uneven number of trials per participant. Perhaps missing or unbalanced data is unusual in the

kinds of tasks that are discussed in this paper, but at the very least it would be good to note somewhere whether the app can be used when participants differ in their number of trials.

Comments on the app:

Nowhere on the app is it noted that this only works for binary data; sooner rather than later someone is going to use it on continuous data and come to a very wrong conclusion.

Mean and sample variance should be clarified, esp. that variance is taken over people (not all trials).

It's not clear why "Time to collect the trials" is included as input – it doesn't look like anything is done with this information.

It took me a while to figure out what the numbers mean when hovering over the plot.

Do "upper limit" and "lower limit" refer to the upper and lower limit of the confidence interval for C, such that the lower limit results in a higher reliability? It is confusing that the lower limit refers to a higher reliability and the upper limit is lower. If it's a confidence interval for C, what percentage of the distribution is covered?

The meaning of the term "calculated trace" will not be apparent to users.

Reviewer #2 (Remarks to the Author):

The authors propose a new web tool which, as they describe it in the Discussion, "could both inform study design by quickly and easily predicting from small amounts of pilot data the necessary number of trials to achieve high reliability, as well as provide a straightforward evaluation and comparison between tasks of their efficiency for individual differences studies…" This is an important endeavor and prima facia seems like a tool that could have some use beyond the Spearman-Brown Prophecy formula. There was a lot to appreciate about the paper; it is important to study and document the relationships between task duration and accuracy – in short, the signal-to-noise obtained given the amount of time spent on the task – is an important aspect of optimizing

time and tolerance for behavioral testing. At the same time I had several concerns that I believe will be useful for the authors to address.

1. Firstly, the authors begin the argument for reliable tasks by describing Marek and colleagues' challenge to the power of neuroimaging studies. However, this study won't deliver on that promise – that more reliable behavioral tasks deliver more power for neuroimaging. Some readers will feel this motivation for the work is a distraction. Furthermore, I've had this very conversation with the authors who will themselves deny that their small effects are a function of reliability, pointing to similar effects from both less (functional) and more (structural) reliability data (albeit both in the imaging modality). That is to say, while I absolutely see that the authors are motivated to bring the role of reliability into hot debates in the field, there is a good deal more ordinance flying across that field than these data will speak to. Behavioral (and similar) data are also increasingly being used for applications like predicting risk, drug response and precision medicine, diagnosis, and success where reliability is just as, if not more, important as correlations with biological data.

2. Regarding the introduction, I don't think that most people believe they have reached a task's reliability ceiling at the end of the task. Researchers I know are looking for "good enough" reliability, or "as good as we're likely to get for the time invested." It is worth communicating that the authors understand this cost-benefit analysis, since the tool they will present is all about informing this cost-benefit calculation.

3. Third, I was also confused by the author's introduction of the issue of "stable, trait-like individual performance across days" in the Discussion. To me seemed the study had been about internal consistency to this point. Considering the data are collected at the same time, the issue of something being stable and trait-like is the domain of retest-reliability and seemed to be a nonsequitur in this instance. Later, though, the authors write "Reliability, as we define it here, is an expansion of the traditional test-retest reliability which involves administering the same task twice to the same group of participants, and then calculating the Pearson's correlation between the two repetitions. This reliability, which is focused on the internal consistency of the scores..." This conflation of retest and internal consistency is going to cause a lot of readers to scratch their heads.

4. p. 4 and Figure 1. In the text you note that data subsamples L are non-overlapping. This non-overlapping requirement is essential to notions of split-half correlation. Consider adding this constraint to the description of L in the caption to reassure readers of the Figure that you are not double dipping from the same data.

5. Table 1 would benefit from citations for the tasks described.

I would request the forgiveness of the authors and editor for any misunderstandings or factual errors I may have made in reading this manuscript, and I wish the authors the best as they move forward in this project. As I usually do, I have signed this review that it may be included with my comments,

Angus MacDonald, III

University of Minnesota

Reviewer #1:

This paper derives a formulation of reliability in terms of the mean and between-person variance of binary data that allows neuroscience researchers to input these summary statistics from pilot data into a web app to get a reliability curve. The resulting reliability curves can be used to adjudicate whether a particular measure is suited for individual differences research, and if it is, how many trials are required to attain acceptable reliability. The novelty of this method is that researchers are not required to compute reliability (e.g., coefficient alpha) from their pilot data, instead it is inferred from the mean and variance statistics.

I fully agree that cognitive scientists need to pay more attention to reliability of their measures (and measurement more generally) if they are interested in pursuing individual differences research. I also think the app is neat, and I hope researchers find it helpful. Technically there's nothing really new here – I'm not sure I agree with the authors that this approach is "much easier" than calculating alpha, which is readily accessible in any statistical software package – but if this paper brings some attention to the reliability phenomena that are documented in it, it will be a valuable contribution to the literature.

My primary concern with this paper is that its audience is not clear. My own area of study is psychometrics, not neuroscience, and I found this paper extremely difficult to follow. The notation used throughout is very different than the standard notation used in psychometrics and psychology, making the whole paper seem far more complicated and impenetrable than it really is. The math is quite simple, but I'm not sure that anyone in neuroscience who reads this will be able to follow.

We thank the reviewer for a thorough review of our paper. We greatly appreciate their enthusiasm for our contribution and recognition of the potential impact on individual differences research. We further thank the reviewer for bringing to our attention the lack of clarity regarding the intended audience. The issue of reliability is, of course, relevant for a very broad audience. Following this comment, as well as comments by reviewer 2, we have tried to better elucidate the intended audience and the importance and implications of reliability for a wide set of problems in the framing of the paper, moving beyond the narrower neuroscience lens. We have thus rewritten the abstract and expanded the Introduction (p. 1-2) as well as the Discussion (p. 28) to include sections on more general uses of behavioral data, which are impacted by issues of reliability.

Additionally, we have considerably expanded the scope of the paper by incorporating analyses of a new dataset that allowed us to address questions of test-retest reliability across days, as opposed to just split-halves reliability, which assumes all trials are equivalent. This resulted in changes to both the Introduction (p. 3) and Discussion (p. 24-25, 28-29), an additional section in the Results (section Effect of time on reliability, p.18-20) and three additional figures – one main text figure and two supplementary information figures (Supplementary Figures 10 and 11). In short, our new findings greatly expand the discussion of reliability and add a number of important observations – there is a large variance in how different tasks are affected when we specifically test how time impacts their reliability, indicating that performance on these tasks is differentially affected by measuring over different time points, and these effects can be broken down into transient effects (over days),

and more long-term effects (over months). We hope these new results will make the paper even more relevant to a broad audience.

To improve the readability of the paper, we have changed the wording and nomenclature to better follow common usage in psychometrics and psychology, hopefully improving accessibility; see also response to comments below.

The paper could be made more readable by making connections between the technical parts and the substantive interpretation. For example, the technical development in this paper is to state reliability in terms of a convergence coefficient “C”, but nowhere in the paper is it made clear how to interpret this coefficient. What are its units? Are there thresholds for when C is so large that a measure shouldn’t be used? It is helpful that Cs are given for a range of tasks, but I’m still not sure what C = 25 means.

We agree with the reviewer that the interpretation of the *C* coefficient in the paper is indeed limited. *C* is a dimensionless coefficient, which describes the (nonlinear) relationship between a given number of trials and the reliability level that will be obtained. As this is a nonlinear relationship, *C* adds information that is not immediately apparent from simply examining the reliability level for a particular number of trials, and in that sense, it is a score of how well the task separates individuals. Its utility is mainly in comparing different tasks and not as an absolute number in itself. For instance, if two tasks are thought to capture the same aspect of cognition, then comparing their *C* coefficients can assist with validating this assumption or selecting the one that would be more suitable for individual differences studies. We initially included an interpretation for the *C* coefficient in the results section, but have added additional context and interpretational guidelines in the text (p. 27-28).

The main utility of C, a dimensionless coefficient, is therefore primarily in comparing different tasks, rather than in the meaning of the absolute number in itself, which is difficult to interpret on its own. For instance, the standard administration of the Cambridge Face Memory Test (CFMT) is more reliable than the Glasgow Face Memory Test (GFMT), and also more suitable for studying individual differences (lower C). Emotion discrimination tasks (emotion labeling and emotion matching) have very low reliability for a standard administration. These tasks also have high values of C, perhaps pointing to a lack of objective answers for these tasks. We provide the range of Cs calculated from our dataset as a starting point (Fig. 7c), to help relate new tasks to an existing, though partial, scale. We expect that as more data accumulates regarding values of C on different tasks, the absolute value in itself will become more informative.

Simulation results in Fig 4. are shown for 250 trials and 100 participants. But the app recommends 50 participants and >20 trials. What are these recommendations built on? Based on Figure 4, it looks like even with N = 100 and 250 trials, there is substantial variability around the estimated C coefficient, which makes sense since reliability is known to be subject to high sampling variability when reliability is low and sample sizes are small. Is there any evidence that the numbers recommended in the app are sufficient for estimation? Figure 5 shows that median error is negligible, but researchers will be subject to error on a single trial, not median error. If I’m reading Fig 5 correctly, then with N = 50 and L = 30, we’re looking at a SD of 60 (80? it’s very hard to differentiate these shades!) percent error, which seems substantial.

As the reviewer pointed out, while the median error is negligible for these N/L pairs, there is still substantial variation in the error of the estimation, which will affect the error of a single trial measurement. The recommendations are based on the finding that beyond *N*=50 and *L*=30, the decrease in error with additional data falls off, making these numbers the elbow of the error curve, and thus a reasonable threshold for data collection. We thank the reviewer for pointing out that this was not previously made clear, as well as the difficulty in differentiating the shading. We have changed the color scale in Fig. 5 to make differences more readily apparent, which should now make the above point clearer. We also now make this point explicit in the Results (p. 15), and have added a supplementary figure which shows the error estimation curves for 30 and 50 participants (Supplementary Figure 8). We have also updated the recommendation to use *L*>=30 trials instead of *L*>20 trials, to better match our data. In any case, the app implements these errors as confidence intervals to make this data readily accessible to users, who can then choose how conservative they wish to be in determining their sample sizes. An additional explanation of the meaning of the shaded error shades in the app was added to the newly created FAQ of the app (to be linked on the app page upon publication, and reproduced below).

p. 15:

As can be seen from all three heatmaps, the median error is negligible and close to zero for N>=50 and L>=30, and the decrease in the standard deviation of the error, while higher than the median error, also falls off sharply beyond this point (see also Supplementary Fig. 8 for curves showing the decrease of the SD of the error with N and L).

"we therefore define reliability (R) as…" what follows is not a definition but a way of estimating reliability.

The sentence on p. 4-5 was rewritten more accurately as follows:

Throughout this paper, we therefore use the definition of reliability (R) as the mean Pearson's correlation across participants on different subsets of data from a given behavioral measure. To calculate this measure of reliability, we use a permutation-based split-half extension of the classic test-retest reliability measure (see Methods)[69,70,73–75] as this method has previously been suggested to be optimal for calculating the internal consistency of cognitive tasks[43,69,75–78]. Note that for this analysis, we pool across all trials regardless of whether they were collected in the same session or not. Later analyses specifically test the effect of the session in which data were collected, see below. In order to calculate reliability for different numbers of trials, we split participants' data into two smaller, non-overlapping batches of L trials, calculate the scores from those batches in each split per participant…

On page 4 the term "samples" is used to refer to number of trials. This was incredibly confusing – it's typical to reserve "sample" and "sample size" and "subsample" to refer to participants, and "trials" for trials.

We thank the reviewer for pointing out this confusion in the notation. This sentence was rewritten to better adhere to common terminology, see the final sentence in the previous response (p. 4-5).

It would be useful to include some discussion of missing data. In some designs (e.g., infant data), it is common for participants to do as many trials as possible before getting fatigued, resulting in an uneven number of trials per participant. Perhaps missing or unbalanced data is unusual in the kinds of tasks that are discussed in this paper, but at the very least it would be good to note somewhere whether the app can be used when participants differ in their number of trials.

The issue of missing data is difficult to address, as any solutions that ignore or impute missing data could have unforeseen or task-specific consequences. There is no simple mathematical solution. At the moment, our recommendation to readers is to follow the same protocol as we adopt in the current paper – discard participants with too much missing data, and ignore missing trials per participant, if only a few trials are missing. We also note that while this is true for our dataset, other datasets might require different handling of missing data. We have added this to the Discussion (p. 28), and to the FAQs of the app.

Even so, some participants were missing some of the data for various reasons. The issue of missing data is complex, as the effect of missing trials in calculating reliability is task dependent (for instance, whether all trials are the same type, same difficulty level, etc.). Our analysis is based on large datasets with overall few missing data points, so we were able to ignore missing data. Participants with many missing data points were excluded based on our other exclusion criteria (see Methods). Different experimental setups might require different approaches to dealing with missing data[109–111].

Comments on the app:

Nowhere on the app is it noted that this only works for binary data; sooner rather than later someone is going to use it on continuous data and come to a very wrong conclusion.

We thank the reviewer for pointing out this omission. Indeed, it is very important to guard against user error as much as possible. We have now added an extensive FAQ to the app, which hopefully will address many of these concerns. Specifically for this point, apart from adding a section to the app, we have also added a note to the subtitle, as this is one of the most critical points in using the app.

Mean and sample variance should be clarified, esp. that variance is taken over people (not all trials).

An explanation was added to the labels (previously in the subtitle only), and further explanation was also added to the FAQ.

It's not clear why "Time to collect the trials" is included as input – it doesn't look like anything is done with this information.

This option was added to help experimenters understand how long their experiment will take to run for a given reliability level, as trial length can vary substantially between different tasks. As time is often the most important resource experimenters have to optimize, and the relationship between *C*, reliability, and number of trials is non-linear, this option is meant to allow the direct comparison of the total time it would take to run two different tasks at the same reliability level. The information of time is displayed on the graph if selected. The explanation for this distinction is now added to the FAQ and further discussed on p. 3.

It took me a while to figure out what the numbers mean when hovering over the plot.

An explanation was added to the FAQ and labels were added when hovering.

Do "upper limit" and "lower limit" refer to the upper and lower limit of the confidence interval for C, such that the lower limit results in a higher reliability? It is confusing that the lower limit refers to a higher reliability and the upper limit is lower. If it's a confidence interval for C, what percentage of the distribution is covered?

The upper and lower limits indeed referred to the confidence margin or, rather, the error in estimating *C* that is associated with the given set of parameters provided by the user. The bounds represent mean error +- mean SD for the given combination of *L* and *N* that were obtained through simulations (Fig. 5). In cases where the SD> 100%, a warning is presented to the user and SD of 100% is used.

We agree with the reviewer that the terminology "lower" and "upper" was confusing, and have therefore renamed the labels, to more accurately indicate what they represent - mean predicted number of trials +/- 1 SD, accordingly. We have also added an explanation in the FAQ.

The meaning of the term "calculated trace" will not be apparent to users.

Changed to mean predicted number of trials.

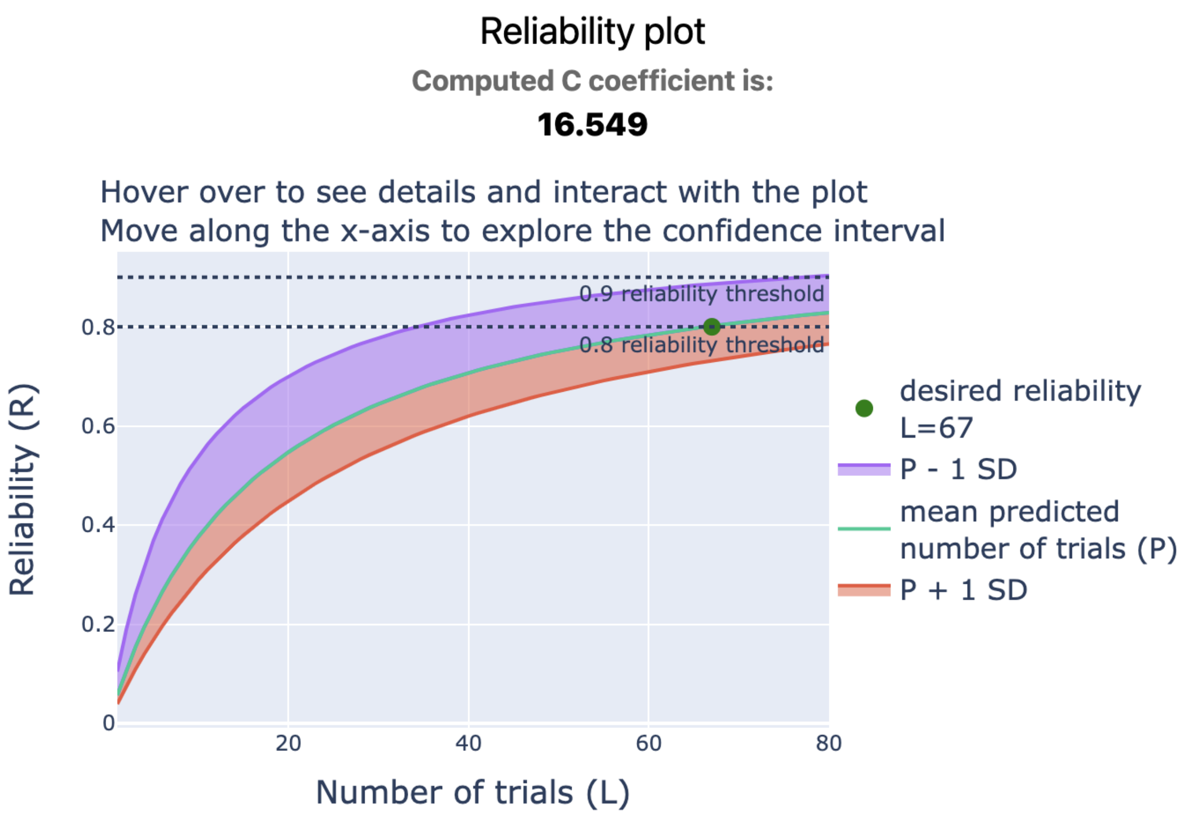

Reviewer #2:

The authors propose a new web tool which, as they describe it in the Discussion, “could both inform study design by quickly and easily predicting from small amounts of pilot data the necessary number of trials to achieve high reliability, as well as provide a straightforward evaluation and comparison between tasks of their efficiency for individual differences studies…” This is an important endeavor and prima facia seems like a tool that could have some use beyond the Spearman-Brown Prophecy formula. There was a lot to appreciate about the paper; it is important to study and document the relationships between task duration and accuracy – in short, the signal-to-noise obtained given the amount of time spent on the task – is an important aspect of optimizing time and tolerance for behavioral testing. At the same time I had several concerns that I believe will be useful for the authors to address.

We thank the reviewer for their insightful review. We appreciate positive remarks on the significance of our web tool for study design and efficiency evaluation in individual differences studies.

1. Firstly, the authors begin the argument for reliable tasks by describing Marek and colleagues’ challenge to the power of neuroimaging studies. However, this study won’t deliver on that promise – that more reliable behavioral tasks deliver more power for

neuroimaging. Some readers will feel this motivation for the work is a distraction. Furthermore, I've had this very conversation with the authors who will themselves deny that their small effects are a function of reliability, pointing to similar effects from both less (functional) and more (structural) reliability data (albeit both in the imaging modality). That is to say, while I absolutely see that the authors are motivated to bring the role of reliability into hot debates in the field, there is a good deal more ordinance flying across that field than these data will speak to. Behavioral (and similar) data are also increasingly being used for applications like predicting risk, drug response and precision medicine, diagnosis, and success where reliability is just as, if not more, important as correlations with biological data.

We thank the reviewer for this perspective and for prompting us to expand the framing of the paper beyond neuroscience. We completely agree that the findings of this paper and the use of the tool should not be limited to neuroscience, and have now rewritten the abstract and expanded the Introduction (p. 1-2) as well as the Discussion (p. 28) to reflect this better. We hope this has now made the paper relevant to a much wider audience, and are thankful for the helpful comment.

As an aside, perhaps as part of a larger debate regarding the interpretation of the findings by Marek et al., we still firmly believe that the findings presented here, and specifically the reliability of behavioral measures, play a crucial role in the hot topic of brain-behavior correlation studies. At the very least, the effects of reliability on observed correlations are dramatic and their relevance to brain-behavior correlation studies, given the low reliability of many of the standard administrations of cognitive behavioral tasks we present here, should be taken into account.

2. Regarding the introduction, I don't think that most people believe they have reached a task's reliability ceiling at the end of the task. Researchers I know are looking for "good enough" reliability, or "as good as we're likely to get for the time invested." It is worth communicating that the authors understand this cost-benefit analysis, since the tool they will present is all about informing this cost-benefit calculation.

We thank the reviewer for pointing out that this important aim was not previously sufficiently well communicated in the paper, as, of course, it is exactly the intention of the app to make this cost-benefit analysis easier for experimenters so that they can optimize their study design based on their existing resources. We have added a section in the introduction explicitly stating this (p. 3):

Although more data enhances reliability, it must be balanced against available time and resources. The convergence rate directly influences what this trade-off function looks like, and making it explicit should aid researchers in optimizing their data collection strategies.

3. Third, I was also confused by the author's introduction of the issue of "stable, trait-like individual performance across days" in the Discussion. To me it seemed the study had been about internal consistency to this point. Considering the data are collected at the same time, the issue of something being stable and trait-like is the domain of retest-reliability and seemed to be a nonsequitur in this instance. Later, though, the authors write "Reliability, as

we define it here, is an expansion of the traditional test-retest reliability which involves administering the same task twice to the same group of participants, and then calculating the Pearson's correlation between the two repetitions. This reliability, which is focused on the internal consistency of the scores…" This conflation of retest and internal consistency is going to cause a lot of readers to scratch their heads.

We are sincerely grateful to the reviewer for pointing out this omission on our part, as it has led to a crucial addition to this paper and perhaps the most important and novel contribution that this paper makes. We had indeed neglected this important distinction between the split-halves analysis which we were using, which pools across all data, and test-retest reliability, which does not assume that data collected at different time points is equivalent. As the data in our battery was mostly collected on at least two separate testing days, following this comment, we directly compared split-halves and test-retest reliability analyses, with surprising results. In short, we found large variance in how different tasks are affected by specifically testing these effects of time on reliability, indicating that performance on these tasks is differentially affected by measuring over different time points. Tasks which were more perceptual in nature were less affected, whereas those with a strong memory or attention component were more affected. This prompted us to add additional datasets to our analysis which were not previously included here, to directly test two different points – the effect of different time spans on this change in test-retest vs. split-halves reliability and the effects of averaging across days. From this, we learned that these effects can be broken down into transient and potentially more long-term effects and that averaging over 2-3 days appears to be sufficient to remove many of the transient effects. This new dataset and new analyses resulted in changes to both the Introduction (p. 3) and Discussion (p. 24-25, 28-29), an additional section in the results (section Effect of time on reliability, p. 18-20), and three additional figures – one main text figure, and two more supplementary information figures (Supplementary Figures 10 and 11). We believe this addition has strengthened the paper substantially and greatly expanded its applicability for a broad range of audiences, and we thank the reviewer once again for pointing us in this direction.

4. p. 4 and Figure 1. In the text you note that data subsamples L are non-overlapping. This non-overlapping requirement is essential to notions of split-half correlation. Consider adding this constraint to the description of L in the caption to reassure readers of the Figure that you are not double dipping from the same data.

We appreciate the reviewer's suggestion to clarify the non-overlapping nature of data subsamples in our paper. We have incorporated this information into the caption of Figure 1. The revised caption now explicitly states that data subsamples L are non-overlapping, thus providing reassurance that there is no double-dipping from the same data.

5. Table 1 would benefit from citations for the tasks described.

We thank the reviewer for their suggestion regarding Table 1. We have added citations for the tasks described in the table.

I would request the forgiveness of the authors and editor for any misunderstandings or factual errors I may have made in reading this manuscript, and I wish the authors the best as they move forward in this project. As I usually do, I have signed this review that it may be included with my comments,

Angus MacDonald, III

University of Minnesota

FAQ about the app

How to use this app

Detailed instructions are provided in the SI of the original article. To use the online web application (https://jankawis.github.io/reliability-web-app/), follow this protocol:

1. Run your pilot experiment with a small number of participants and trials per participant (we recommend at least N=30, though preferably N~50 participants, and L>=30 trials per participant).
   Using a smaller number of trials or participants is possible, but will result in larger confidence intervals.

2. Calculate the mean score for each participant across trials.

3. Calculate the mean ($E[Z]$) and sample variance (Var(Z)) of those scores across the group of scores. Sample variance is the default in many statistical tools (pandas, R, Matlab) except for numpy where ddof=1 has to be explicitly used.

4. Enter the mean and variance computed in step 3., and the number of participants and trials per participant into the online tool and plot the corresponding reliability curve.

Toggle the reliability R to see how many trials L you need to reach your desired reliability level. You can also optionally provide the time it took to collect these L trials and plot reliability versus the time it would take to collect the necessary number of trials.

In some cases, for which the calculated C is very high, corresponding to very low variance (potentially as a result of floor or ceiling effects), the app will provide a warning. In such cases, the use of that measure for studying individual differences should be reconsidered.

A warning will also be issued if the error of the fit cannot be properly estimated for very small combinations of L and N.

Note that a further **limitation** is that the MV fit implemented in the app requires measures to be sampled from a Bernoulli distribution (i.e., each trial can yield only two possible outcomes). For any other measures which do not obey these assumptions, we suggest applying the directly fitted or linearized fits (not through the app). See *What are the limitations of this app? Can I use it for any task?* section of the FAQ.

What are the limitations of this app? Can I use it for any task?

Unfortunately not. This web app uses a method that is limited to binary tasks, meaning tasks in which the outcome of any given trial is binary and there are only two possible outcomes (0/1, correct/incorrect, blue/red, etc.). Multiple choice tasks with more than two alternatives can be used as long as the outcome of the trial is binary (e.g., tasks with 4 options, 1 correct and 3 incorrect). Examples where the online tool **cannot** be directly used are any difference measures (difference of scores in trials of category "A" and category "B", e.g. before and after some intervention, or of different difficulties), composite measures such as d', Cowan's k, beta, Likert scale scores, etc.

However, if your task is NOT binary, you **can** still compute the *C* coefficient and create the reliability curve using the other two methods – direct and linearized fits. Consult please our paper to find out more.

What to do with missing data per subject?

This question is complex and task-dependent. Our recommendation at the moment is to follow the same protocol as we adopted in the current paper – discard participants who have too many missing data points, and utilize functions such as nanmean to calculate summary statistics by ignoring missing data. It is also possible to downsample the number of trials per participant to the minimum number of trials available for all participants. If this significantly changes the mean and variance of the distribution, perhaps the inclusion of participants with missing data should be reconsidered.

Which variance should I use? Sample or population?

You should use sample variance of participants' scores. This means calculating the scores for all participants and then determining the variance of this set/distribution of scores. It is the default in pandas, R, and Matlab. If you use numpy, you need to manually set ddof=1 (see https://numpy.org/doc/stable/reference/generated/numpy.var.html). If you are an Excel user, please use VAR.S (or VARA, https://support.microsoft.com/en-gb/office/var-s-function-913633de-136b-449d-813e-65a00b2b990b).

What is the "Time to collect the trials" good for?

The optional "Time to collect the trials" performs a simple calculation of the time that it will take to collect data to achieve the desired reliability. It is to give you a sense of the practical implications for collecting a given number of trials and help inform decisions about the reliability vs time to collect data trade-off. If you provide the

necessary information, you can toggle the "Plot time" button and see the reliability with experiment time rather than the number of trials.

What are the upper and lower limits (P + 1SD and P - 1SD) and which one should I use? (aka How to read the reliability plot)

Short answer: Move along the x-axis to explore the confidence interval. The lower limit is the minimum number of trials for a desired reliability (purple, P - 1SD), while the upper limit (red, P + 1SD) ensures you shouldn't need more trials than specified to achieve that reliability level.

Long answer: The upper and lower limits in the reliability plot show the confidence margin, or the error, in estimating the *C* coefficient based on the user-provided parameters. It is a range indicating the mean error plus or minus the mean standard deviation (SD) for the given combination of *L* and *N* that were determined through simulations (refer to Figure 5 in the paper).

In cases where the SD is greater than 100%, a warning is presented to the user and SD of 100% is used.

To understand the "lower" and "upper" bounds: focus on the reliability (y-axis) of the plot and follow a line parallel with the x-axis. Intercepts with the curves will tell you the lower and upper limits.

Lower Limit (P - 1SD): This tells you the minimum number of trials needed to achieve a specific reliability. Less than this number of trials will NOT reach the desired reliability level.

Upper Limit (P + 1SD): This indicates that you won't need more trials than this number to achieve the desired level of reliability. It ensures you're on the safe side.

What do the numbers mean when hovering over the plot?

It is the (x,y) coordinate of the point, so the first says the number of trials (*L*) and the second is reliability (*R*) corresponding to this number of trials.

The app does not allow me to enter a mean that is less than zero or more than 1. What should I do?

If your mean is less than 0 or more than 1, it very likely means that the outcomes of trials in your experiment or the measure you use are not binary (see section *What are the limitations for this app? Can I use it for any task?*) and this tool **cannot** be used for your data. If, however, your task is binary, this may simply mean that you need to scale your data appropriately (for instance – a participant might have a score of 0.8 instead of 80% correct, or 40/50 trials correct). If your task is not binary, you can still create the reliability curve

yourself, calculate C and obtain reliability predictions based on the direct and linearized fits. Please see the main text for details on how to compute these curves.

11th Mar 24

Dear Michal,

Thank you for your patience during the peer-review process. Your manuscript titled "Putting cognitive tasks on trial: A measure of reliability convergence" has now been seen by 2 reviewers, and I include their comments at the end of this message.

Reviewer #2 is satisfied with the changes you have made in revision, but Reviewer #1 voices outstanding concerns.

We remain interested in the possibility of publishing your study in Communications Psychology, but would like to consider your responses to these concerns and assess a revised manuscript before we make a final decision on publication. The referee's concerns must be addressed through revisions to the manuscript.

As discussed via email, we are happy to continue to consider your contribution as an Article, rather than a Resource. As you revise the work to address the Reviewer's comments, we ask you to work with the enclosed checklist for Articles, as we believe that some issues will be resolved if presentational issues are addressed. Amongst these presentational issues is our request to adopt the Introduction-Methods-Results-Discussion order of sections. Prominently implementing visual display items that form part of the Methods, such as simulations or depictions of the model is advisable. These changes will help you to set out the research question, how it was addressed, and what results it delivered in a manner that will bring greater conceptual clarity and focus.

The Introduction will benefit from adopting a stronger focus on existing definitions and on the literature and research questions that the research will inform, and might perhaps better eschew rhetorical questions.

We are committed to providing a fair and constructive peer-review process. Please don't hesitate to contact us if you wish to discuss the revision in more detail.

I am attaching an Editorial Requests Table that details critical reporting requirements for the revised manuscript. Please attend to each item and ensure your manuscript is fully compliant. We are requesting that your manuscript aligns with these requirements as this facilitates the evaluation of your manuscript, reducing delays in re-review and potential future acceptance. If your revised manuscript is not aligned with these requests on major issues, such as those concerning statistics,

it may be returned to you for further revisions without re-review. Additional information can be found in our style and formatting guide Communications Psychology formatting guide.

Please use the following link to submit your

- revised manuscript,

- point-by-point response to the referees' comments,

- cover letter (as a separate document),

- the Editorial Policy Checklist (see below),

- the Reporting Summary (see below), and

- the completed Editorial Request Table (attached):

[link redacted]

** This url links to your confidential home page and associated information about manuscripts you may have submitted or be reviewing for us. If you wish to forward this email to co-authors, please delete the link to your homepage first **

We hope to receive your revised paper within 8 weeks; please let us know if you aren't able to submit it within this time so that we can discuss how best to proceed. If we don't hear from you, and the revision process takes significantly longer, we may close your file. In this event, we will still be happy to reconsider your paper at a later date, provided it still presents a significant contribution to the literature at that stage.

We would appreciate it if you could keep us informed about an estimated timescale for resubmission, to facilitate our planning.

We look forward to seeing the revised manuscript and thank you for the opportunity to review your work.

Best regards,

Marike

Marike Schiffer, PhD

Chief Editor

Communications Psychology

REVIEWER REPORTS:

Reviewer #1 (Remarks to the Author):

Thanks to the authors for responding to my previous review. I think I was able to understand more of the paper this time around, and my resulting impression is that this paper lacks a strong basis in psychometric theory. In particular, the concept of measurement error is missing from the definition of reliability and the derivation of the formulas, which results in some confusion about what reliability is and how it influences correlations and outcomes, and how the coefficient C can be interpreted. The survey of behavioral outcomes and their reliability is useful, and there are some nice demonstrations of well-known properties of reliability, but none of these are explained as clearly as they could be. I also continue to be uncertain about the usefulness of sample estimates of C, given the apparent imprecision of its estimate in reasonably sized samples.

Many places in the text suggest confusion about the meaning of reliability and stability. For example, on page 2, the question is posed whether modest test-retest reliability estimates arise because "behavior truly does not converge to a more stable performance, perhaps influenced by momentary, non-specific fluctuations in a non-measured variable such as attention" or whether "insufficient data [is] being used to estimate individual abilities". But "momentary, non-specific fluctuations in a non-measured variable" are exactly the kind of variables that are usually considered to be measurement error, i.e., the non-reliable component of a measure. A measure that is strongly impacted by fluctuations like this is an unreliable measure, and a way to make it more reliable is to aggregate more replications of the measure. Neither of these options describes actual fluctuations in the trait that is being measured, which would result in a low estimate of test-retest "reliability" (which in this case is not truly reliability but true change). Some version of the phrase "converges to a stable mean" is repeated often, but it is never clear what it means. Throughout the paper, this confusion about what instability across measurement occasions indicates persists.

The definition of reliability given "the mean Person's correlation across participants on different subsets of data from a given behavioral measure" is still not a definition. Reliability is a population

parameter that can be defined and described without reference to any given sample. Various test theories delineate assumptions (e.g., parallel tests, stable traits) under which particular sample estimates (like alpha or split-half or test-retest reliability) give unbiased estimates of the population quantity. The delineation between population quantity and sample estimate needs to be made clear, and doing so will help to clarify when various estimates are actually valid (e.g., test-retest reliability does not estimate reliability if the trait being measured is not stable).

All error bars in figures and in the app represent one SD, meaning that these represent 68% confidence intervals. Given the norms in the field of using alpha levels of .05 and interpreting 95% intervals, it would be more useful if these were 95% confidence intervals instead. This note is especially important for Fig 4, where the distribution of estimated values of C are shown.

I'm not sure what message is conveyed by Figure 1. If the point is to show that reliability increases with number of trials, why bother computing 1000 split-halves and then computing their mean, rather than just directly computing coefficient alpha for each value of L and plotting that? It is well known that just using one arbitrary split-half is inefficient, and I don't think that's relevant to the message but I'm not actually sure what the figure is trying to convey. A more useful error bar would be to compute the analytical confidence interval of coefficient alpha and plot that, as it represents expected sampling variability of the most efficient measure of reliability rather than permutation variability of an inefficient measure.

Fig 2 – are error bars still SDs over different split-halves?

Fig 3 – I'm not sure why the blue dot-lines and the red lines differ. To derive the attenuation formula estimated values, what population correlation value was used? I'm also not sure how to read the conclusion "Note that overestimation is very unlikely when the true underlying correlation is high" from this figure.

Derivations:

- Sample notation (e.g., y_bar) and population expectations (e.g., E(y)) are used interchangeably, to confusing effect.

- The derivations make no contact with psychometric theory, and they are so hard to get through for that reason. The whole business of ordering by proficiency is unnecessary to get to the endpoint of the Spearman-Brown formula. The result is that C seems like some uninterpretable quantity. If you start from classical test theory (e.g., see Lord and Novick, 1968), the quantity that is called B is just error variance (for a single trial), and the quantity that is called A is true-score variance, and the "consistency coefficient" is just error variance over true score variance. It's a totally interpretable quantity, but it is made to become completely mysterious.

- “since the outcomes of the trials for a participant are independent ... var(x_i) = L*var(d_i)”. The only way for outcomes of trials for a participant to be independent is if the trials are not measuring anything stable. This definition appears to assume that there are no stable individual differences, at least until p comes in. I think this is another example of confusion arising unnecessarily as a result of trying to define reliability without having a conception of measurement error.

Reviewer #2 (Remarks to the Author):

None.

EDITORIAL POLICIES

We ask that you ensure your manuscript complies with our editorial policies and reporting requirements.

To that end, we require revised manuscripts to be accompanied by two completed items: a reporting summary that collects information on study design and procedure, and an editorial policy checklist that verifies compliance with all required editorial policies.

Nature Research Reporting Summary

Editorial Policy Checklist

All points on the policy checklist must be addressed. Your revised manuscript can only be sent back to the referees if these checklists are completed and uploaded with the revision.

Notes: If you have submitted a Stage 1 Registered Report, Review, Primer, Comment, or Perspective you do not need to submit these forms. If you have already submitted these forms, you may disregard this request.

Reviewer #1:

Thanks to the authors for responding to my previous review. I think I was able to understand more of the paper this time around, and my resulting impression is that this paper lacks a strong basis in psychometric theory. In particular, the concept of measurement error is missing from the definition of reliability and the derivation of the formulas, which results in some confusion about what reliability is and how it influences correlations and outcomes, and how the coefficient C can be interpreted. The survey of behavioral outcomes and their reliability is useful, and there are some nice demonstrations of well-known properties of reliability, but none of these are explained as clearly as they could be. I also continue to be uncertain about the usefulness of sample estimates of C, given the apparent imprecision of its estimate in reasonably sized samples.

Many places in the text suggest confusion about the meaning of reliability and stability. For example, on page 2, the question is posed whether modest test-retest reliability estimates arise because "behavior truly does not converge to a more stable performance, perhaps influenced by momentary, non-specific fluctuations in a non-measured variable such as attention" or whether "insufficient data [is] being used to estimate individual abilities". But "momentary, non-specific fluctuations in a non-measured variable" are exactly the kind of variables that are usually considered to be measurement error, i.e., the non-reliable component of a measure. A measure that is strongly impacted by fluctuations like this is an unreliable measure, and a way to make it more reliable is to aggregate more replications of the measure. Neither of these options describes actual fluctuations in the trait that is being measured, which would result in a low estimate of test-retest "reliability" (which in this case is not truly reliability but true change). Some version of the phrase "converges to a stable mean" is repeated often, but it is never clear what it means. Throughout the paper, this confusion about what instability across measurement occasions indicates persists.

We thank the reviewer for a thorough review of our paper and for their insightful comments, our responses to which we hope have now helped us ground the paper more solidly in psychometric theory. We have extensively reworked the relevant sections of the introduction to explicitly address and better explain the distinction we were attempting to make between measurement error and fluctuation in the measured abilities themselves as it pertains to complex cognitive tasks. We have also attempted to better position this discussion within the existing framework of psychometric literature and to fit our terminology to its more standard use in the field. There are changes in terminology throughout the paper, but the main change to the introduction is in this section (p.3):

"Split-halves reliability (comparing a random half of the data to the other half) could be affected by momentary, non-specific fluctuations in a non-measured variable such as attention. Test-retest reliability, which compares scores across different testing times, would be even more sensitive to such state changes, for instance in attention, arousal, motivation, etc. While this might be considered measurement error, such variables could interact directly with the ability being tested, especially in complex cognitive tasks. For example, attention could be considered an inherent process involved in memory tasks, directly influencing your ability to encode the stimuli, rather than an independent variable. This would mean that any fluctuations in measured scores could be a true change in abilities (e.g., ability to remember the stimuli) rather than measurement error, making these two difficult to separate (for

instance see discussion in chapter 2 of Lord&Novick[cit]). For test-retest measures, there could also be additional longitudinal change in the true measured ability, especially as time between measurements increases.

Examining how split-halves and test-retest reliabilities converge as more trials are added will allow us to test whether the low reliability scores reported for cognitive tasks are a function of insufficient data being used to estimate individual proficiencies, or whether they are the result of true change in individual abilities."

The definition of reliability given "the mean Person's correlation across participants on different subsets of data from a given behavioral measure" is still not a definition. Reliability is a population parameter that can be defined and described without reference to any given sample. Various test theories delineate assumptions (e.g., parallel tests, stable traits) under which particular sample estimates (like alpha or split-half or test-retest reliability) give unbiased estimates of the population quantity. The delineation between population quantity and sample estimate needs to be made clear, and doing so will help to clarify when various estimates are actually valid (e.g., test-retest reliability does not estimate reliability if the trait being measured is not stable).

We thank the reviewer for pointing out the lingering imprecision in our definitions. We have made changes throughout the paper to clarify when sample or population estimates are being used, for example:

"Throughout this paper, we estimate reliability (R) for our sample as the mean Pearson's correlation across participants on different subsets of data from a given behavioral measure."

All error bars in figures and in the app represent one SD, meaning that these represent 68% confidence intervals. Given the norms in the field of using alpha levels of .05 and interpreting 95% intervals, it would be more useful if these were 95% confidence intervals instead. This note is especially important for Fig 4, where the distribution of estimated values of C are shown.

We thank the reviewer for this suggestion. As noted, a single estimate of a measured variable, or a single estimate of C using one sample, is noisy. The error bars in both the figures and the app are meant to represent a reasonable estimate of the distribution of expected values over many such calculations. While alpha of .05 is standard when claiming that results are significant notwithstanding a narrow margin of error, the error bars in our figures and in the app are not meant to show the full range of values you might potentially get for a single calculation, but rather the range you are most likely to fall within, as a reasonable starting point for making experimental design and data collection decisions. For this purpose, we believe that changing this value to 2 SDs, to be more in line with an alpha of .05, will be less rather than more informative for getting a sense of what the likely values are and for making experimental decisions, as extreme values, while rare, can certainly occur. Following this comment and the comment on Fig 2. below, we have ensured that it is clear in all relevant places in the text and figure legends that all error bars indeed represent

1 SD, and not an alpha of .05. For further clarity and consistency, we have also removed Fig. 4a from the paper that showed model comparison of the 3 fits and used interquartile range as it was causing confusion with the error of 1 SD shown in the rest of the article, and was redundant with the information presented in Fig. 4b (now 4a).

I'm not sure what message is conveyed by Figure 1. If the point is to show that reliability increases with number of trials, why bother computing 1000 split-halves and then computing their mean, rather than just directly computing coefficient alpha for each value of L and plotting that? It is well known that just using one arbitrary split-half is inefficient, and I don't think that's relevant to the message but I'm not actually sure what the figure is trying to convey. A more useful error bar would be to compute the analytical confidence interval of coefficient alpha and plot that, as it represents expected sampling variability of the most efficient measure of reliability rather than permutation variability of an inefficient measure.

We thank the reviewer for pointing out the confusion regarding the role of Figure 1. The point of this figure was merely to explain the exact calculation of the repeated split-halves reliability measure we use throughout the paper. We have now explicitly clarified this in the text:

"Figure 1 shows our methodology in calculating the reliability for different numbers of trials."

Fig 2 – are error bars still SDs over different split-halves?

The label was indeed missing from the figure caption. In all panels (a-d) of Fig. 2, the error bars are the SDs of the distribution of correlations per given L. We have added this to the caption.

Fig 3 – I'm not sure why the blue dot-lines and the red lines differ. To derive the attenuation formula estimated values, what population correlation value was used? I'm also not sure how to read the conclusion "Note that overestimation is very unlikely when the true underlying correlation is high" from this figure.

We are grateful to the reviewer for pointing out this omission in our explanation of the figure. We have substantially expanded the explanation of the figure in the text, and also added an explanation for the discrepancy between the two lines (p.16):

"Importantly, low reliability tends to cause an underestimation of the true correlation values. This is in line with the well described attenuation effect first put forward by Spearman[70,109,110], in which the mean value of the observed correlation is lower than the true underlying correlation, as our data in Figure 3a-b demonstrates. The left panels of the figure show the distributions of observed correlations between the two measures across all subsamples. The correlation between the full dataset of both tasks is shown by the red line. Note that when observing high correlation values ($|r|>0.4$), this correlation is unlikely to be spurious (highly overestimated). However, when the true correlation is very low, as in Figure 3c, observed

correlations on a single "snapshot" of an experiment (i.e., one iteration of the split-halves analysis) can both underestimate or overestimate the true correlations.

The right panels show the same data, with each point depicting the mean of the histogram on the left for the given reliability level, and error bars denoting 1 SD of the distribution (blue lines). The red line plots the correlation predicted by Spearman's attenuation prediction formula for different reliability levels, applied to the correlation value between the full datasets of both tasks (denoted by the red line in the plots on the left). Even though the full dataset is more reliable than the highest level of subsampling we reach, it is a single measure, and as such also appears to underestimate the true correlation when that correlation is high, accounting for the lower values of the red line in panels a and b but not in panel c."

Derivations:

- Sample notation (e.g., y_bar) and population expectations (e.g., E(y)) are used interchangeably, to confusing effect.

We thank the reviewer for pointing this out; we have gone over the derivation and changed all notations to be consistent.

- The derivations make no contact with psychometric theory, and they are so hard to get through for that reason. The whole business of ordering by proficiency is unnecessary to get to the endpoint of the Spearman-Brown formula. The result is that C seems like some uninterpretable quantity. If you start from classical test theory (e.g., see Lord and Novick, 1968), the quantity that is called B is just error variance (for a single trial), and the quantity that is called A is true-score variance, and the "consistency coefficient" is just error variance over true score variance. It's a totally interpretable quantity, but it is made to become completely mysterious.

We thank the reviewer for this comment and the extremely relevant reference. We have added the names of coefficients A and B to the derivations as well as to the main text to make the article more accessible and understandable to people from psychometric circles and to increase clarity.

- "since the outcomes of the trials for a participant are independent … var(x_i) = L*var(d_i)". The only way for outcomes of trials for a participant to be independent is if the trials are not measuring anything stable. This definition appears to assume that there are no stable individual differences, at least until p comes in. I think this is another example of confusion arising unnecessarily as a result of trying to define reliability without having a conception of measurement error.

We appreciate the reviewer's comment and would like to provide further clarification. The trial outcomes in our study are independent, much like individual coin flips, where each flip does not influence the next and is not influenced by previous outcomes. However, this independence does not imply a lack of stability in measurement. A loaded coin, for instance, can still yield independent flips or trials but consistently exhibit a mean which is different from the expected value of 0.5. Similarly, in experiments, independent trials converge towards a

certain value (proficiency), reflecting the inherent "unfairness" of the coin. This convergence arises because the measured variables are not random and exhibit stability over repeated trials.

Reviewer #2:

None

We are pleased that this reviewer was fully satisfied with our earlier revisions.

23rd May 24

Dear Michal,

Your manuscript titled "Putting cognitive tasks on trial: A measure of reliability convergence" has now been seen by our reviewers, whose comments appear below. In light of their advice I am delighted to say that we are happy, in principle, to publish a suitably revised version in Communications Psychology under the open access CC BY license (Creative Commons Attribution v4.0 International License).

We therefore invite you to revise your paper one last time to address the remaining concerns of our reviewers and a list of editorial requests. At the same time we ask that you edit your manuscript to comply with our format requirements and to maximise the accessibility and therefore the impact of your work.

EDITORIAL REQUESTS:

Please review our specific editorial comments and requests regarding your manuscript in the attached "Editorial Requests Table". Please outline your response to each request in the right hand column. Please upload the completed table with your manuscript files as a Related Manuscript file.

If you have any questions or concerns about any of our requests, please do not hesitate to contact me.

SUBMISSION INFORMATION:

In order to accept your paper, we require the files listed at the end of the Editorial Requests Table; the list of required files is also available at https://www.nature.com/documents/commsj-file-checklist.pdf .

OPEN ACCESS:

Communications Psychology is a fully open access journal. Articles are made freely accessible on publication under a CC BY license (Creative Commons Attribution 4.0 International License). This

license allows maximum dissemination and re-use of open access materials and is preferred by many research funding bodies.

For further information about article processing charges, open access funding, and advice and support from Nature Research, please visit https://www.nature.com/commspsychol/article-processing-charges

At acceptance, you will be provided with instructions for completing this CC BY license on behalf of all authors. This grants us the necessary permissions to publish your paper. Additionally, you will be asked to declare that all required third party permissions have been obtained, and to provide billing information in order to pay the article-processing charge (APC).

* TRANSPARENT PEER REVIEW: Communications Psychology uses a transparent peer review system. On author request, confidential information and data can be removed from the published reviewer reports and rebuttal letters prior to publication. If you are concerned about the release of confidential data, please let us know specifically what information you would like to have removed. Please note that we cannot incorporate redactions for any other reasons.

* CODE AVAILABILITY: All Communications Psychology manuscripts must include a section titled "Code Availability" at the end of the methods section. We require that the custom analysis code supporting your conclusions is made available in a publicly accessible repository at this stage; please choose a repository that generates a digital object identifier (DOI) for the code; the link to the repository and the DOI must be included in the Code Availability statement. Publication as Supplementary Information will not suffice.

* DATA AVAILABILITY:

All Communications Psychology manuscripts must include a section titled "Data Availability" at the end of the Methods section. More information on this policy, is available in the Editorial Requests Table and at http://www.nature.com/authors/policies/data/data-availability-statements-data-citations.pdf.

Please use the following link to submit the above items:

[link redacted]

** This url links to your confidential home page and associated information about manuscripts you may have submitted or be reviewing for us. If you wish to forward this email to co-authors, please delete the link to your homepage first **

We hope to hear from you within two weeks; please let us know if you need more time.

Best regards,

Marike

Marike Schiffer, PhD

Chief Editor

Communications Psychology

REVIEWERS' COMMENTS:

Reviewer #2 (Remarks to the Author):

In my last review of the manuscript, I was satisfied with the authors' response to the reviews. Reviewer 1 however had a number of new concerns that had arose following the revision that, upon reading them, I thought would be useful to readers to have addressed.

I have now reviewed the exchange between Reviewer 1 and the authors with some interest and as a potential user. Retest-reliability (more so than internal consistency) seems to be having a moment. So there is an audience for these discussions. While there remain some differences between

Reviewer 1 and the authors, I believe the process so far has honed the document and the remaining differences do not seem to be prohibitive to its value or the value of the tool. I am therefore satisfied that the concerns have been addressed.

I would again request the forgiveness of the authors and editor for any misunderstandings or factual errors I may have made in reading this manuscript, and I wish the authors the best as they move forward in this project. As I usually do, I have again signed this review that it may be included with my comments,

Angus MacDonald, III

University of Minnesota

Reviewer #1:

Reviewer #2:

In my last review of the manuscript, I was satisfied with the authors' response to the reviews. Reviewer 1 however had a number of new concerns that had arose following the revision that, upon reading them, I thought would be useful to readers to have addressed.

I have now reviewed the exchange between Reviewer 1 and the authors with some interest and as a potential user. Retest-reliability (more so than internal consistency) seems to be having a moment. So there is an audience for these discussions. While there remain some differences between Reviewer 1 and the authors, I believe the process so far has honed the document and the remaining differences do not seem to be prohibitive to its value or the value of the tool. I am therefore satisfied that the concerns have been addressed.

I would again request the forgiveness of the authors and editor for any misunderstandings or factual errors I may have made in reading this manuscript, and I wish the authors the best as they move forward in this project. As I usually do, I have again signed this review that it may be included with my comments,

Angus MacDonald, III

University of Minnesota

We thank the reviewer for their comments. We are pleased that the reviewer was fully satisfied with our earlier revisions as well as with our response to Reviewer 1.
